# The dynamics of cooperation, power, and inequality in a group-structured society

**DOI:** 10.1038/s41598-021-97863-7

**Published:** 2021-09-21

**Authors:** Denis Tverskoi, Athmanathan Senthilnathan, Sergey Gavrilets

**Affiliations:** 1 National Institute for Mathematical and Biological Synthesis, University of Tennessee, Knoxville, TN 37996 USA; 2 Center for the Dynamics of Social Complexity, University of Tennessee, Knoxville, TN 37996 USA; 3 Department of Ecology and Evolutionary Biology, University of Tennessee, Knoxville, TN 37996 USA; 4 Department of Mathematics, University of Tennessee, Knoxville, TN 37996 USA

**Keywords:** Applied mathematics, Social evolution

## Abstract

Most human societies are characterized by the presence of different identity groups which cooperate but also compete for resources and power. To deepen our understanding of the underlying social dynamics, we model a society subdivided into groups with constant sizes and dynamically changing powers. Both individuals within groups and groups themselves participate in collective actions. The groups are also engaged in political contests over power which determines how jointly produced resources are divided. Using analytical approximations and agent-based simulations, we show that the model exhibits rich behavior characterized by multiple stable equilibria and, under some conditions, non-equilibrium dynamics. We demonstrate that societies in which individuals act independently are more stable than those in which actions of individuals are completely synchronized. We show that mechanisms preventing politically powerful groups from bending the rules of competition in their favor play a key role in promoting between-group cooperation and reducing inequality between groups. We also show that small groups can be more successful in competition than large groups if the jointly-produced goods are rivalrous and the potential benefit of cooperation is relatively small. Otherwise large groups dominate. Overall our model contributes towards a better understanding of the causes of variation between societies in terms of the economic and political inequality within them.

## Introduction

Throughout our evolutionary history, humans have lived and interacted in groups. Group living implies cooperation but also competition and conflicts between groupmates as well as conflicts between individual and group interests, i.e. social dilemmas^1–4^. Such processes underlying group living are present at all levels of biological organization^5^. In our close relatives chimpanzees, males within a band compete for mating opportunities but cooperate in border patrols aiming to reduce the strength of a neighboring band^6^. Similarly, human groups are engaged in cooperation but also in various types of conflicts including power struggles aiming to shape between-group interactions and social institutions regulating them to their own advantage. As Aristotle put it, “man is by nature a political animal”^7^. Examples of groups engaged both in cooperation and power conflicts are common in modern human societies. These include social classes, political parties, and different ethnic, religious, or regional groups.

Power struggles often lead to power inequality which then translates into economic inequality and other types of inequality. Horizontal inequality, which is inequality between different identity groups in modern societies, is an important topic of study in economics, sociology, social anthropology, and political science^8–11^. Horizontal inequality negatively affects economic efficiency^12^, the production of public goods^13^ and government efficiency^14^, and it often leads to social instability and conflicts^15^. Inequality also negatively affect the well-being of citizens in different ways especially when it becomes institutionalized (e.g., as studied in the Social Dominance Theory^16^). Between-group inequality affected the historical development and survival of many tribes, chiefdoms, states, and empires^17–19^. To better understand these processes, we need to consider the dynamics of collective action^4,20–27^ in cooperation and conflict at multiple levels^5^.

In the fields of biological and cultural evolution, there is now an extensive theory of “multilevel selection” describing both within-group cooperation and between-group competition. The former is usually modeled by linear public goods games (PGG). The latter is usually described by models of differential group survival adapted from population genetics in which between-group interactions are indirect^28–30^ but some models consider direct conflicts as well^31–33^. Usually competition at the group level happens globally, i.e. each group competes with all other groups with equal intensity^34–36^, but see, e.g., a recent model^37^ in which groups interact locally.

There are a variety of models in economics that describe between-group contests. In these models, cooperative groups secure a higher share of contested resource or have higher probabilities to win the contest^38,39^. Most of these models implicitly equate the power of the group with its effort in the contest which controls the share of the resource it secures. However there are also models of between-group conflict with a broader interpretation of power. For example, Refs.^40–42^ modeled contests for power between two or three factions in the society (e.g. the elite, middle class, and commoners or the authoritarian government and the military or two political groups), the winner of which determines the economic and political outcomes (e.g., democratic or despotic). Ref.^43^ studied how the equilibrium contributions to conflict depend on the indices of inequality, fractionalization, and polarization^44^ in the society. These studies highlight the political aspects of human societies which play an important role in their dynamics.

Previous work based on non-cooperative game theory has largely ignored the possibility of between-group cooperation. In a rare exception, Refs.^45,46^ studied a multilevel game in which individuals are engaged in a cascade of different hierarchical PGGs. However there was no between-group competition in their models. There is also a diversity of models from cooperative game theory focusing on coalition formation^47^. In these models, the power of individual factions is constant and determined endogenously, while economic factors are usually disregarded.

Recently Ref.^48^ introduced a novel approach for modeling cooperation and conflict in a society composed by multiple factions engaged in economic and political interactions. In their model, which follows the general approach of Ref.^49^, factions are engaged in an economic game and a separate political game about power. Specifically, at each time step the factions first cooperate or defect in an economic collective goods game played according to the current state of a dynamic set of rules. Then they participate in a contest for the power to change the rules of the economic game to be played at the next time step, in terms of how the collective goods are divided among the factions. This model was an extension of a model^50^ describing non-equilibrium dynamics of resources and power in a society engaged in the redistribution of a fixed amount of resource. In Ref.^48^ model there are three possible outcomes: complete loss of cooperation, stable hierarchy (where one faction persists on top of the hierarchy with some fluctuations in the power of other factions), and continuous turnover (where cycles of cooperation and defection are coupled with cycles in power and inequality). However their model as well as that of Ref.^50^ described the processes of societal evolution only at a meso-scale^51^ and did not consider individuals explicitly. Therefore, the model neglects the collective action problem at the within-faction level and the effects of the group size^20^.

Here we seek to remove these limitations. Specifically, we investigate the joint dynamics of three important processes: within-group cooperation in production of public goods, between-group cooperation in production of collective club goods (i.e., collective goods which are excluded from non-cooperating groups), and between-group contest for the shares of jointly produced collective goods. In our framework, the collective action problem is present at both within- and between-group levels. We assume that both individuals and groups are bounded rational: they use myopic best response (with errors) to make their strategic decisions. We explicitly model the dynamics of power focusing on the effects of a parameter measuring the strength of mechanisms preventing politically powerful factions from bending the rules of competition in their own favor^40,41,52,53^. We investigate the effects of the degree of rivalry of the goods produced and allow for groups to have different sizes^54^. The latter feature let us study Olson’s group-size paradox^20,55–60^. We aim to shed light on the following questions: when and why cooperation emerge in group-structured societies? What are the causes of variation between societies in terms of economic and political inequality within them? What are the effects of checks-and-balances preventing politically powerful factions from bending the rules of competition in their own favor on cooperation and inequality? How do the group size and within-group interactions affect cooperation and inequality at the between-group level?

## The model

**Figure 1:**
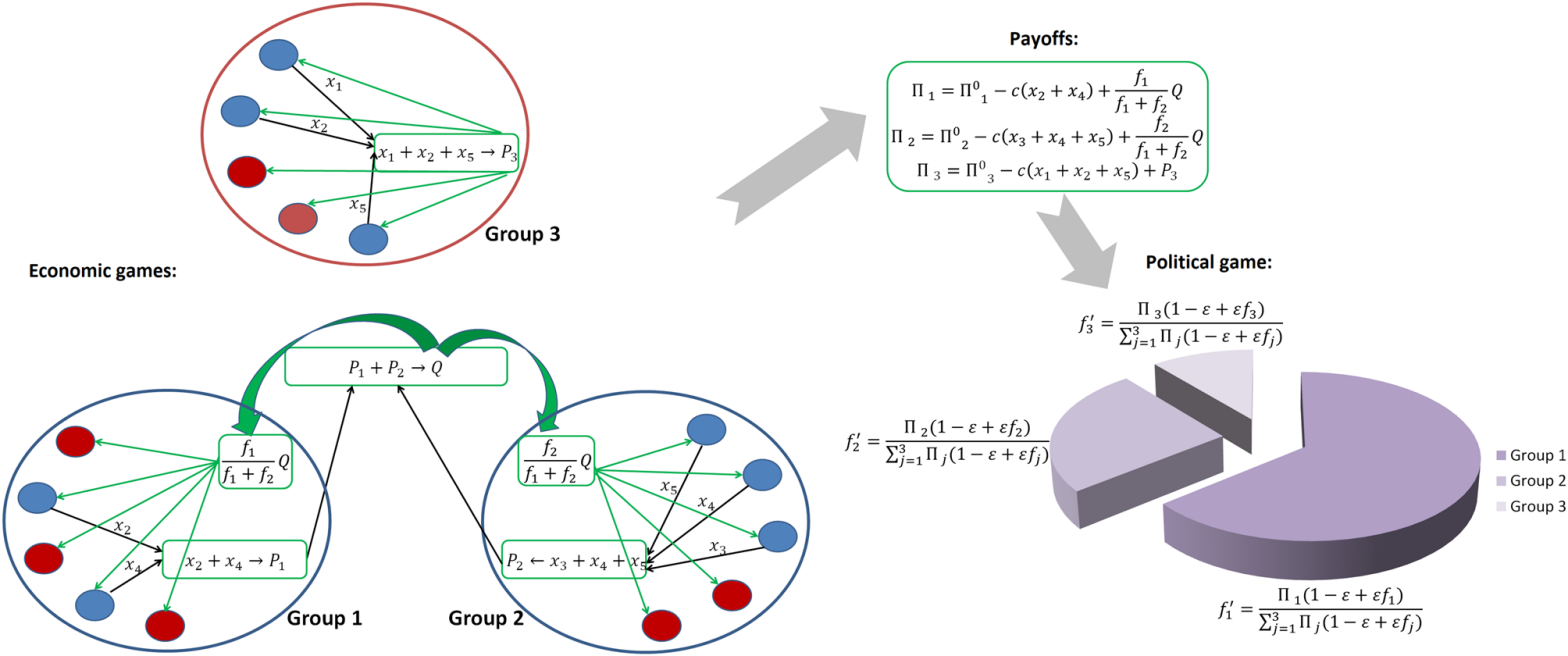
The model structure. Shown is an example of a society with three groups each with 5 individuals. First, individuals and groups are engaged in economic games. Individuals cooperating in within-group game (in blue) contribute to group production $P_i$; defecting individuals are shown in red. Groups 1 and 2 contribute their production $P_1$ and $P_2$ to cooperate in the between-group game and produce resource *Q* to be divided according to their relative power. Group 3 defects and just keeps the resource $P_3$ it produced. After that groups contribute the effort $\Pi _i (1-\varepsilon +\varepsilon f_i)$ to a political game the result of which modifies their political power from $f_i$ to $f_i'$.

We consider a society composed by *G* groups which interact repeatedly in time. Time is discrete. Let $n_j$ and $f_j$ be the size and political power of group *j* ($0 \le f_j \le 1$, $\sum f_j=1$). Individuals within each group are engaged in an economic game leading to the production of certain resources. Group members can divide these resources among themselves equally or invest into another economic game at the level of groups. Groups also participate in a separate political game about power to obtain a share of the jointly produced resources which then are divided equally within each group. For example, from about 100 to 700 CE some societies in the Moche Valley, Peru, were organized as a collection of interacting villages differing in power which depended on the role in religious rituals. The villages cooperated in building irrigation systems but also competed over the extent of control over them^61^. One can also think of any modern country where different states politically compete for shares of national resources or jointly produced domestic products. Figure 1 illustrates the structure of our model.

### Within-group economic game

At the beginning of each time step, each individual *i* in a group *j* has a baseline amount of resource $\pi ^0>0$. First, each individual makes a decision to contribute ($x_{ij}=1$) or not ($x_{ij}=0$) to the group effort $X_j=\sum _i x_{ij}$. The cost of contribution is *c* ($0<c\le \pi ^0$). The resource $P_j$ produced by the group as a result of within-group cooperation is an increasing function of the combined effort $X_j$ of group members^4^:

1 $$\begin{aligned} P_j=B_1 \frac{X_j}{X_j+X_0}. \end{aligned}$$

Here $B_1$ is the maximum possible benefit of the within-groups cooperation and $X_0$ is a half-effort parameter (which specifies the level of group effort at which $P_j=B_1/2$). Equation (1) describes a public good game with non-linear production function and diminishing marginal return of group productivity^62,63^. Parameters $B_1$ and $X_0$ can reflect the quality of the environment experienced by groups.

### Between-group economic game

After completion of within-group games, each group (or its representative) decides on whether to keep the produced benefit ($\theta _j=0$) or invest it in between-group cooperation ($\theta _j=1$). Cooperating groups can be viewed as a coalition of elites; defecting groups can be viewed as counter-elites^64^. We assume that groups with $X_j=0$ are never part of the elites.

Let *C* be the size of the coalition, i.e. the number of groups that have decided to participate in the between-group game. Let $Z=\sum P_j$ be their combined contribution. We postulate that the resource *Q* produced as a result of between-group cooperation is an increasing function of *Z*^4^:

2 $$\begin{aligned} Q=B_2 \frac{Z}{Z+Z_0}, \end{aligned}$$

where $B_2$ is the maximum possible benefit and $Z_0$ is a half-effort parameter at the group level. Parameters $B_2$ and $Z_0$ can reflect the quality of the environment experienced by the society. Note that both games utilize a nonlinear production function used in earlier studies of “us vs. nature” and “us vs. them” games^4, 65–67^. This function is more realistic than a standard linear production function in both capturing diminishing marginal return of productivity and allowing for partial participation (i.e., a situation where cooperators and defectors coexist) expected in many real-world situations.

A cooperating group *j* gets a share $v_j$ of the produced resource which is equal to its relative power within the coalition:

3 $$\begin{aligned} v_j=\frac{f_j}{\sum _k f_k}, \end{aligned}$$

where the sum is over the set of all cooperating factions. In this model of “club goods”^68^, only the coalition of elites share the amount of goods *Q* dividing them according to their power, whereas the counter-elites just keep their own production $P_j$.

The total material payoff obtained by group *j* is thus

4 $$\begin{aligned} \Pi _j= \Pi _j^0-cX_j + {\left\{ \begin{array}{ll} P_j,\ &{}\text {if the group defects}\\ v_j Q\, &{} \text {if the group cooperates}. \end{array}\right. } \end{aligned}$$

where $\Pi _j^0=n_j \pi ^0$ is the total baseline resource of group *j*.

After completion of the games, the resource obtained by each individual in group *j* represents a $1/n^{\alpha }_j$-share of its group resource. Here parameter $0 \le \alpha \le 1$ characterizes the degree of rivalrousness of the goods^55,56^. For example, if $\alpha =1$, the goods are fully rival. In contrast, if $\alpha =0$, the goods are pure public. For $\alpha >0$ increasing the size of the group decreases the individual’s share/value, while if $\alpha =0$, the individual’s share/value does not depend on the group size. Ref.^69,70^ show that many publicly provided goods exhibit a high degree of rivalry (i.e., $\alpha$ is high).

Correspondingly, the payoff to each individual is

5 $$\begin{aligned} \pi _{ij}= \pi ^0-cx_j + {\left\{ \begin{array}{ll} P_j/n^{\alpha }_j,\ &{}\text {if the group defects}\\ v_jQ/n^{\alpha }_j\, &{} \text {if the group cooperates}. \end{array}\right. } \end{aligned}$$

Below in illustrating our results, we will also use the normalized parameters

6 $$\begin{aligned} b_{1,j}=B_1/n^{\alpha }_j,\ b_2=B_2/\sum _{j}n_j^{\alpha }. \end{aligned}$$

The former is the maximum benefit of within-group cooperation per individual. The latter is the maximum benefit of between-group cooperation per individual while assuming an equal division of the jointly produced reward.

### Between-group political game

After completion of economics games, all groups are engaged in a political game that results in a modification of political powers. Specifically, we define the effective effort of group *j* in the political game as

7 $$\begin{aligned} y_j=\Pi _j(1-\varepsilon +\varepsilon f_j) \end{aligned}$$

and postulate that the group *j* power at the next time step is given by the Tullock contest success function^38^:

8 $$\begin{aligned} f_j'= {\left\{ \begin{array}{ll} \frac{y_j}{\sum y_k}, &{}\text { if } \sum y_k>0 \\ \frac{1}{G}, &{}\text { otherwise.} \end{array}\right. } \end{aligned}$$

The incumbency effect parameter $\varepsilon$ controls the strength of dependence of $y_j$ on power $f_j$ ($0 \le \varepsilon \le 1$). If $\varepsilon =0$, then $y_j = \Pi _j$ and only the amount of the faction’s material resource $\Pi _j$ matters; if $\varepsilon =1$, then $y_j = \Pi _j f_j$, so that the material resource and power combine multiplicatively in defining $y_j$. The smaller this parameter is, the stronger are the forces in the society (such as the rule of law, checks and balances, and democratic institutions) preventing politically powerful factions from bending the rules of competition in their own favor^71,72^. With larger values of $\varepsilon$, politically powerful factions manage to increase disproportionately their shares of resources.

### Strategy revision and decision-making

Each individual updates their strategy in the within-group economic game randomly and independently with a fixed probability $\mu _1$. Each group updates its strategy in the between-group economic game randomly and independently with a fixed probability $\mu _2$. Both individuals and groups use myopic best response subject to random errors to maximize their material payoff. Specifically, when making decisions, each updating individual always compares the expected payoffs of two actions ($x=0$ and $x=1$) and chooses the action which gives the higher payoff (with precision $\lambda$ as specified in the Quantum Response Equilibrium approach^73^). Similarly, each updating group chooses the action, $\theta =0$ or $\theta =1$, which gives the higher payoff. The decisions are made synchronously for all updating individuals first, then for all updating groups. After that the power of groups is updated. In the main text, we focus on the case of infinite precision $\lambda =\infty$.

All model parameters are assumed to be time-independent. Table S1 in the Supplementary Materials (SM) summarizes the variables, functions, and constant parameters of our model.

## Results

Our model exhibits a very rich behavior: it can have multiple simultaneously stable equilibria and also show non-equilibrium dynamics. “Methods” section summarizes our analytical results on some symmetric equilibria. Here we discuss more complex dynamics observed in numerical simulations. In our simulations, for generating the initial distribution of power we use a “broken stick distribution”^74,75^, if groups have equal size; and assume that initially groups have equal power, if they have different sizes. We assume that initially, each individual and each group cooperate randomly and independently with probability 0.5. To estimate characteristics of long-term dynamics, the model was run 100 (or 200) times for 4000 time steps and the statistics were computed over the last 1000 time steps.

**Figure 2:**
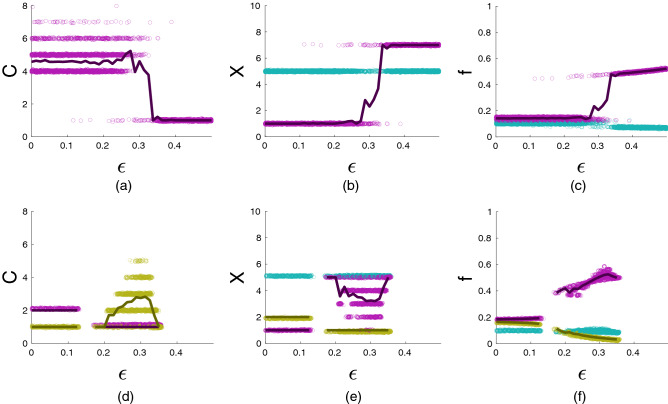
Effects of the incumbency parameter $\varepsilon$ on the number of cooperating groups *C* (**a**,**d**), the number of cooperating individuals per group *X* (**b**,**e**) and group power *f* (**c**,**f**). First row of graphs: equilibria with just one type of cooperating groups. Defecting groups are shown in blue symbols. Second row of graphs: equilibria with dominant (violet symbols) and subordinate (golden symbols) cooperating groups. Curves show the average values of corresponding characteristics. The equilibria illustrated in the top and the bottom rows are simultaneously stable. Eight groups of the same size $n=10$. Other parameters: $b_1=20$, $b_2=10$, $\alpha =1$, $c=1$, $\pi ^0=1$, $X_0=5$, $Z_0=50$, $\mu _1=\mu _2=0.25$. The results shown are based on 200 runs with 4000 time steps for each parameter combination. The outcomes for each run are averages of the last 1000 time steps.

### Groups with identical sizes

We start by assuming that groups have equal size *n*. In this case, two possible types of dynamics are observed: equilibrium and non-equilibrium. We will describe them separately focusing on the effects of parameters. Throughout we will assume that the ratio of the maximum group benefit $B_1$ to the group cost $cX_0$ at half-effort $R_1=B_1/(c X_0)$ is sufficiently large so that each group has at least one cooperating member. Here we discuss numerical results for the case of groups with $n=10$ individuals. Our analytical results and additional numerical simulations (Figs. S10–S12 in the SM) show that groups of other sizes have similar behavior.

#### Equilibria

Convergence to an equilibrium is the most common type of dynamics. The structure of equilibria is very complex: there are many of them and they can be locally stable simultaneously (see section 5.1 in the SM and Fig.  9). These equilibria share some properties. Specifically, at equilibrium, all non-cooperating groups (i.e. counter-elites) have the same number of contributing individuals and have the same power. Among cooperating groups (i.e, elites), all groups can have the same number of cooperators and same power (equal elites) or there can be just two types of groups, which we will call dominant and subordinate (non-equal elites). All dominant groups have the same number of cooperating individuals and the same power. All subordinate groups also have the same number of cooperating individuals and the same power which however is smaller than that of dominant groups.

For small values of the incumbency parameter $\varepsilon$, there can be several stable equilibria with equal (Fig. 2a) and non-equal elites (Fig. 2d). For intermediates values of $\varepsilon$, the number of stable equilibria can increase (Fig. 2a,d). The number of cooperators *X* in a cooperating group can be either smaller or larger than that in a defecting group (Fig. 2b,e). The number of cooperators *X* in subordinate cooperating groups can be either larger or smaller than that in dominant groups (Fig. 2e). In general, non-cooperating groups are less powerful than dominant groups (Fig. 2f). They are less powerful than subordinate groups, if the incumbency parameter $\varepsilon$ and the benefit per individual $b_2$ are small; and more powerful than subordinate groups, if the incumbency parameter is relatively large (Fig. 2f). Increasing the incumbency parameter $\varepsilon$ increases power of cooperating groups relative to that of non-cooperating groups in the equilibria of the first type (Fig. 2c). For additional examples see Figs. S5–S9 in the SM. The resource *Q* produced in an equilibrium with $C=G$ equal cooperating groups is large compared to other equilibria. However, the maximum amount of *Q* is observed when there is one dominant group and $G-1$ subordinate groups (see Fig. S13 in the SM).

Figure 2f shows that for relatively high values of $\varepsilon$, subordinate groups (marked by golden color) do not switch to defection in spite of the fact that defecting groups (marked by blue color) have higher power. Because such subordinate groups have a very low number of contributors (see Fig. 2e) simply defecting will only decrease their power. To make defection pay, they would also need to increase the number of contributors. Planning two-steps ahead however is not allowed within myopic best response updating we use here.

**Figure 3:**
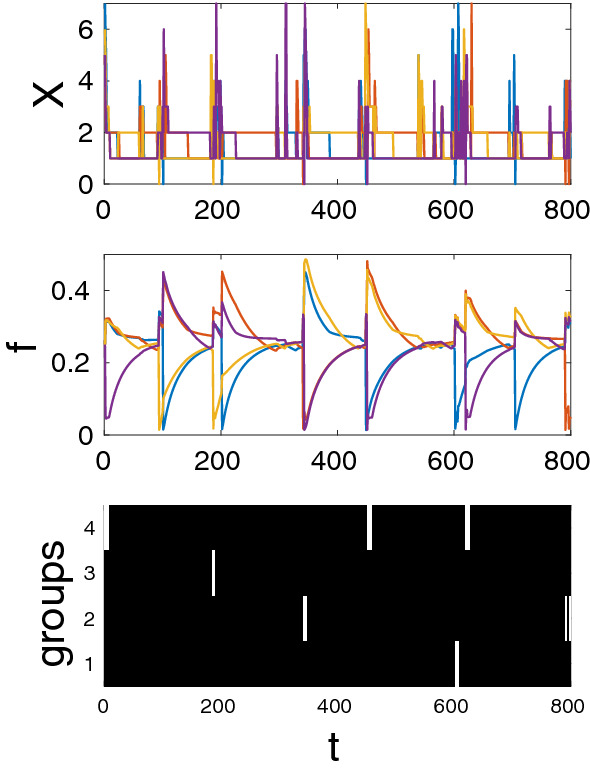
An example of non-equilibrium dynamics. Top: the number of contributing individuals in each group. Middle: faction powers. Bottom: groups cooperating at time *t* (i.e., those with $\theta _j=1$) are shown as black pixels, while defecting groups (i.e., those with $\theta _j=0$) are shown as white pixels. Four groups of size $n=10$ each. Other parameters: $b_1=10$, $b_2=26$, $\varepsilon =0.1$, $c=1$, $\pi ^0=1$, $X_0=5$, $Z_0=50$, $\mu _1=\mu _2=0.25$.

#### Non-equilibrium dynamics

Non-equilibrium dynamics mostly happen when the incumbency parameter $\varepsilon$ is small and only within certain ranges of other parameters (for details see Figs. S14, S15 in the SM). In this regime the cooperating coalition typically includes all groups but the number of cooperating individuals within groups fluctuates. These fluctuations are coupled with fluctuations in power, which, in turn, lead to a turnover of dominant groups. Below variable $\theta _j=1$ if group *j* cooperates in the coalition of “elites” and $\theta _j=0$ if not. An example of non-equilibrium dynamics shown in Fig. 3.

#### Effects of parameters

We will focus on the number of cooperating groups *C*, the Gini index of inequality in power among them *I*, and the standard deviation $\sigma$ of cooperating group efforts. The Gini index is mathematically equivalent to half of the relative mean absolute difference.

**Figure 4:**
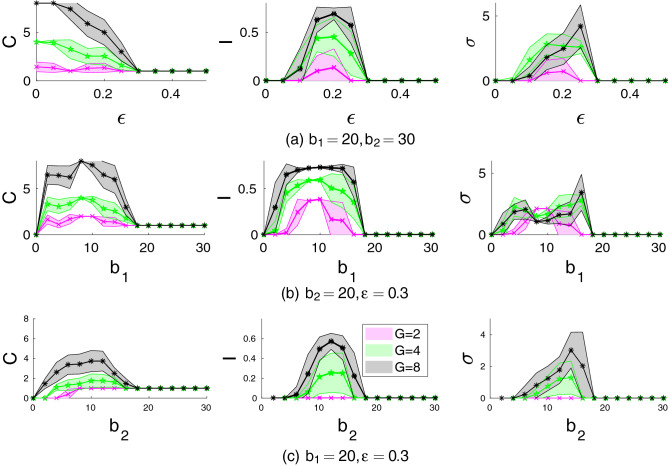
Effects of (**a**) the incumbency parameter $\varepsilon$; (**b**) the benefit parameter $b_1$; and (**c**) the benefit parameter $b_2$ on the number of cooperating groups *C*, the Gini index of inequality *I*, and standard deviation of efforts $\sigma$ among them for different number of groups *G*. Groups of the same size $n=10$ are considered. The shaded areas shows the corresponding confidence intervals. Other parameters: $\alpha =1$, $c=1$, $\pi ^0=1$, $X_0=5, Z_0=50$, $\mu _1=\mu _2=0.25$. The figures show the averages and confidence intervals based on 100 runs each of 4000 time steps for each parameter combination. Results in each run are averages based on the last 1000 of time steps.

##### Incumbency parameter $\varepsilon$

Increasing $\varepsilon$ decreases the size *C* of the cooperative coalition (Fig. 4a) as low-power groups do not receive large enough share of the jointly produced resource and leave the coalition. With sufficiently large $\varepsilon$, only one group remains engaged in the between-group economic game. The inequality in power and group efforts (Fig. 4a) among cooperating groups exhibit a hump-shaped dependence on $\varepsilon$. For more details see Fig. S16 in the SM.

##### Benefit of within-group cooperation $b_1$

When $b_1$ is small, increasing it increases between-group cooperation. However, when $b_1$ is high enough, the benefit of within-group cooperation exceeds that of between-group cooperation leading to a decline in cooperation (Fig. 4b). This process accelerates for higher $\varepsilon$ (for more details see Figs. S17–S19 from the SM). The inequality in power (Fig. 4b) and group efforts (Fig. 4b) among cooperating groups exhibit a hump-shaped dependence on $b_1$.

##### Benefit of between-group cooperation $b_2$

Effects of $b_2$ depend on the incumbency parameter $\varepsilon$ (see Figs. S20, S21 in the SM). If $\varepsilon$ is small, increasing $b_2$ increases the number of cooperating groups *C*. There is no inequality between cooperating groups for small $b_2$ but then it starts slowly increasing once $b_2$ is sufficiently large. For intermediate values of $\varepsilon$, increasing $b_2$ first leads to an increase in the coalition size which then shrinks to just one group as $b_2$ becomes large enough (Fig. 4c). The inequality in power (Fig. 4c) and group efforts (Fig. 4c) among cooperating groups exhibit a hump-shaped dependence on $b_2$. With large $\varepsilon$, the coalition is never large as one of its members quickly increases in power which causes all other groups to defect.

Effects of parameters $Z_0, \lambda$ and *G* are discussed in the SM.

### Groups with different sizes

**Figure 5:**
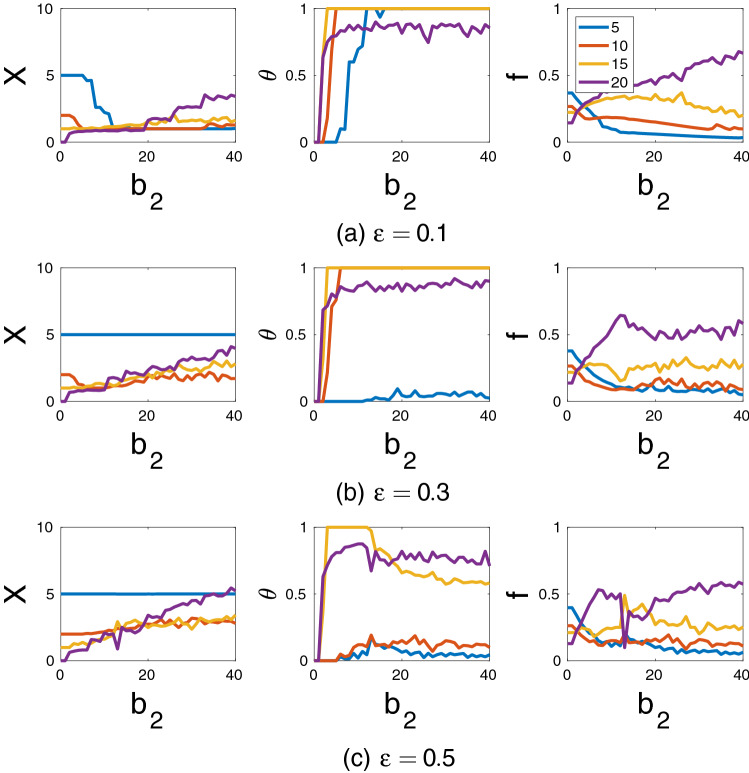
Effects of the benefit parameter $b_2$ and the incumbency parameter $\varepsilon$ on the average number of contributing individuals *X*, the average cooperation status $\theta$ and average power *f* of each group. Groups with 5, 10, 15 and 20 individuals are considered. Baseline parameters: $B_1=100$, $\alpha =1$, $c=1$, $\pi ^0=1$, $X_0=5$, $Z_0=50$, $\mu _1=\mu _2=0.25$. The figures show the averages based on 200 runs each of 4000 time steps for each parameter combination. Results in each run are averages based on the last 1000 time steps.

Differences in group sizes have three structural effects. Increasing the group size *n*: (1) increases the group’s total baseline amount of resource $\Pi ^0$ making it more powerful, (2) decreases shares of the resources $1/n^{\alpha }$ of each group member making cooperation more difficult (if $\alpha \ne 0$); and (3) increases the maximum possible group effort *X*. Because in our models within-group cooperation is typically low, the last effect is weak. The trade-off between the first two effects drives the dynamics of the model. Below first we analyze the average effects of various parameters while keeping the sizes of groups constant. Then we consider the effects of changing the size of one group. At the end, we discuss non-equilibrium dynamics in more details.

#### Effects of parameters

 We will consider four groups with 5, 10, 15 and 20 individuals, respectively. We assume that all other parameters are identical between different groups and set groups’ initial power to 1/4.

If the benefit of between-group cooperation per individual $b_2$ is small so that no between-group cooperation is observed, smaller groups make larger efforts and have higher power in the case of rivalrous goods ($\alpha =1$; see Fig. 5). This is well in line with Olson’s group-size paradox^20^. In contrast, with non-rivalrous goods ($\alpha =0$), efforts of smaller groups do not exceed efforts of larger groups and they obtain lower material payoffs and power than larger groups (see Figs. S30, S31 in the SM). Increasing $b_2$ brings more potential benefits of between-groups cooperation. As a result, some groups switch to cooperation with the largest group typically being the first to do so (see Fig. 5).

If $\varepsilon$ is small, then all groups, one by one will switch to cooperation as $b_2$ grows (see Fig. 5a) independently of the degree of rivalrousness $\alpha$ (see Figs. S27–S31 in the SM). For intermediate and large values of $\varepsilon$, smaller groups usually remain as defectors (see Fig. 5b,c). Larger groups are more successful within the cooperative coalition (i.e., they have higher powers, obtain higher payoffs and are characterized by higher levels of a within-group cooperation compared to smaller groups from the coalition) regardless of $\alpha$ (see Figs. S27–S31 in the SM). It implies that high benefits of group interactions result in the disappearance of Olson’s group-size paradox independently of rivalrousness.

Overall, the effects of other parameters are similar to those in models with equal group sizes (for details see Fig. 5, Figs. S27–S31 from the SM). However, while in the former case which groups cooperate and which defect is mostly determined by initial conditions and chance, in the later case it strongly depends on the group size.

**Figure 6:**
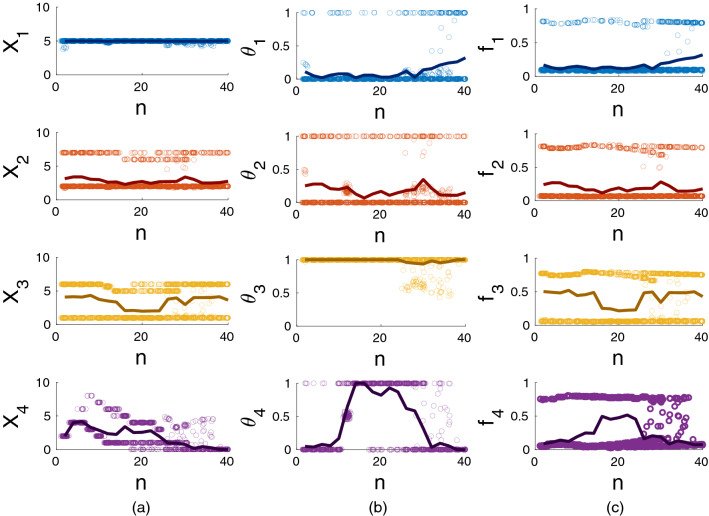
Effects of the group size *n* on the number of contributing individuals in a group *X* (**a**), the cooperation status of a group $\theta$ (**b**) and group power *f* (**c**). Each point corresponds to an outcome of a particular run. The values of $\theta$ larger than 0 but smaller than 1 indicate non-equilibrium dynamics. Characteristics of groups with 5, 10, 15 and *n* individuals are marked by blue, red, yellow and violet colors respectively. Curves show the average values of corresponding characteristics among all runs. Other parameters: $B_1=100$, $B_2=1500$, $\varepsilon =0.5$, $\alpha =1$, $c=1$, $\pi ^0=1$, $X_0=5$, $Z_0=300$, $\mu _1=\mu _2=0.25$. These results are based on 100 runs with 4000 time steps for each parameter combination. The outcomes of each run are averages of the last 1000 time steps.

#### Effect of changing the group size

 Here we assume that there are 3 groups of 5, 10 and 15 individuals, respectively, plus one additional focal group of size *n* which we will vary. For small and intermediate values of *n*, the likelihood of cooperation for the focal group increases with *n* (see Fig. 6b) independently of rivalrousness (see Figs. S32–S45 in the SM). Most often the largest group has the highest power within the cooperative coalition regardless of $\alpha$ (see Figs. S32–S45, right panels). However, the effects of further increases in *n* depend on the degree of rivalrousness. With non-rivalrous goods further increasing *n* promotes growth in the focal group power, which, in turn, stimulates other groups to defect. This process is slowing down by a decrease in the incumbency parameter. Eventually, for very high values of *n*, only the focal group remains engaged in the between-group economic game. For more details see Figs. S40–S45 in the SM.

**Figure 7:**
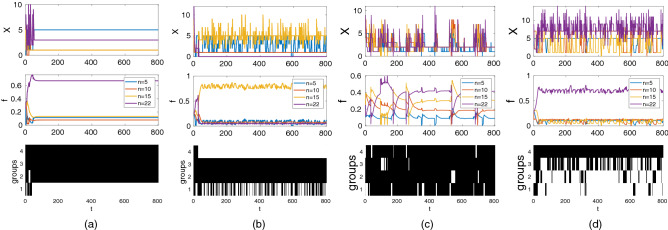
The dynamics of three groups of 5, 10, 15 individuals respectively and the focal group of *n* individuals are illustrated. Examples of two main types of dynamics for high values of *n* are shown: (**a**) the focal group cooperates and has the highest power among all groups; (**b**) there is no contributing individuals in the focal group. Baseline parameters: $n=22$, $B_1=100$, $B_2=1500$, $\alpha =1$, $\varepsilon =0.3$, $c=1$, $\pi ^0=1$, $X_0=5$, $Z_0=300$, $\mu _1=\mu _2=0.25$. Examples of non-equilibrium dynamics for (c) $\varepsilon =0$; and (d) $\varepsilon =0.3$. Baseline parameters: $n=20$, $B_1=300$, $\alpha =1$, $b_2=40$, $c=1$, $\pi ^0=1$, $X_0=5$, $Z_0=300$, $\mu _1=\mu _2=0.25$.

With rivalrous goods, further increases in *n* can lead to the loss of cooperation within the focal group (Figs. 6, 7b). Nevertheless, equilibria with the largest group characterized by the highest power within the coalition can still be observed (Figs. 6, 7a). Decreasing $\varepsilon$, $Z_0$ and increasing $B_2$ increase the value of the focal group size *n* for which the likelihood of the focal group cooperation starts to decline with further increase in *n*; and the value of the focal group size for which the focal group always defects with further increase in *n*. For more details see Figs. S32–S39 in the SM.

#### Non-equilibrium dynamics

 Here we illustrate two interesting types of non-equilibrium dynamics. The first type occurs when the system repeatedly transitions between equilibria with *C* and $C+1$ cooperative groups (see Fig. 7d). In this regime, which is observed if $\varepsilon$ is intermediate, a subordinate group in the coalition switches to defection once its power becomes low enough. This causes a decline in the resource obtained by the dominant group resulting in decreases in its power. Once it has become sufficiently low, the most powerful among the defecting groups returns to the coalition and the process repeats.

The second type of non-equilibrium dynamics is observed if $\varepsilon$ is small (see Fig. 7c) when all groups cooperate. In these dynamics, the cooperating coalition typically includes all groups but their powers as well as the number of cooperating individuals within each of them fluctuate. This regime is similar to that observed if groups have the same size. See Figs. S46–S49 in the SM for more details on the effects of various parameters.

## Discussion

An important feature of many human societies is the existence of different identity groups (e.g., ethnic, cultural, religious, political) which are engaged in economic cooperation but simultaneously are involved in competitive interactions juggling for political power. These dynamics often lead to the emergence of different types of (horizontal) inequalities between identity groups which often undermine economic developments and trigger conflicts within society and its instability^12–15^. Understanding these processes is complicated by the fact that individuals also interact at the within-group level. Our main goal here was to introduce a general theoretical framework for addressing a number of important questions including: when are societies composed of groups differing in power more (or less) stable? When is horizontal inequality high (or low)? When is cooperation and production at the society level high (or low)?

Our model has several realistic features which have been largely neglected in earlier work. Most importantly it considers the joint dynamics of cooperation and competition between different identity groups in the society while explicitly accounting for individual behavior. We allowed for differences between groups in their sizes and changing political power and explicitly focused on the effects of checks and balances mechanisms limiting the ability of powerful groups to grab more power. We considered the effects of inequality, environmental conditions, and rivalrousness of produced collective goods on cooperation and social dynamics.

Our models aim to capture important properties of historic and modern societies. For example, construction of some large irrigation canals in the Moche Valley, Peru, involved multiple villages^61^. Maintaining the irrigation system (e.g., doing regular cleaning) required a large collective effort^76^. Ref.^77^ suggested that villages competed for the control over irrigation systems and the lands that they watered, and this control was performed in a sophisticated power-based way via construction of temples. One can also think of any multi-ethnic modern country where different ethnic groups politically compete for shares of national resources or jointly produced domestic products. Examples include Mizrahi and Ashkenazi Jews ethno-national groups which cooperated to built the Israel state but also competed for real and symbolic resources^78^; and Muslim and Christian communities in Ghana which cooperate and collaborate for producing communal goods, but also compete with each other^79^.

In our model, the most common outcome of social dynamics is a stable society in which certain groups form a cooperating coalition with a certain distribution of power while other groups remain outside of it. Continuous cycles of cooperation and defection of groups, which were prevalent in Refs.^48,50^ (which neglected within-group collective action problem and dynamics) never arose in our model. Although overall the spectrum of possible dynamics observed in our model was broader than that in Refs.^48,50^, non-equilibrium dynamics were observed under a much narrower range of parameters (see below). Therefore we can conclude that within-group dynamics can stabilize the system’s behavior and that a society with individuals acting independently is more stable than a society for which actions of individuals within groups are completely synchronized.

In our model, economic inequality is present at both individual and group levels. Inequality between individuals is a result of differences in individual efforts (with free-riders obtaining higher benefits) and differences between the groups they belong to. The latter are caused by differences in baseline resources, group sizes, and their power. We have shown that inequality among groups can be mitigated by decreasing the incumbency parameter $\varepsilon$. In our model, a society consisting of equal cooperating groups can exist only if the incumbency parameter is relatively low and groups have the same size. Such a society would produce large but not the maximum possible amount of the resource *Q*. If groups are of equal size, the maximum amount of the resource is produced if all groups in the society cooperate, but one of them is dominant in power and makes a disproportional high effort. This effect of between-group differences is analogous to the effects of within-group heterogeneity on collective action^66^.

We observed two different types of non-equilibrium regimes. In the first regime, observed for small values of the incumbency parameter $\varepsilon$, the cooperating coalition typically includes all groups but the number of cooperating individuals within the groups fluctuate. These fluctuations are coupled with fluctuations in power, which, in turn, may lead to the turnover of dominant groups. Turnover of governing parties in democratic states can be viewed as an example of such dynamics. In the second regime, observed for intermediate values of the incumbency parameter $\varepsilon$, one group persists at the top of the coalition with some fluctuations in the identity of other coalition members. In this regime, the growing power of the dominant group forces subordinate groups to leave the coalition decreasing production. Declining production decreases the power of the dominant group which makes it beneficial for the most powerful among the defecting groups to return to the coalition thereby completing the cycle.

Overall, the incumbency parameter $\varepsilon$ plays a key role in our model. This parameter controls the extent to which the differences in economic resources between groups are translated in the differences in power. We have interpreted $\varepsilon$ as a measure of the strength of democratic checks-and-balances or “the rule of law” mechanisms working to prevent politically powerful factions from bending the rules of competition in their favor (smaller values of $\varepsilon$ implies stronger checks-and-balances mechanisms). We have found that reducing $\varepsilon$ promotes cooperation, reduces variation in power and, hence, mitigates between-group inequality. The effects of other parameters strongly depend on $\varepsilon$ as well. In particular, increasing potential benefits of between-group cooperation promotes it only if the incumbency parameter is low. For intermediate values of $\varepsilon$, the cooperative coalition size *C* first increases with the benefit of between-group cooperation but then shrinks to just one group as the benefits become very large (see Fig. S20 in the SM). As a result, the model predicts that promoting cooperation and reducing inequality via increased benefits of cooperation works properly only in societies with strong democratic checks-and-balances. These results are well in line with the empirical literature: political institutions play a key role in increasing economic efficiency and shaping economic growth^80–83^. Non-democratic societies with bad institutions (e.g., institutions that work mostly for the benefit of the ruling elite) often exhibit very low levels of economic growth coupled with deep economic crises and even civil wars^84^. However, some non-democratic regimes can be very successful in terms of economic development (such as former dictatorships of the East Asian “tigers”, such as Malaysia, Singapore, Taiwan, and South Korea). Their success arose partially due to strong nominally-democratic institutions aiming to ensure the cooperation between elites and counter-elites^81,84^.

Differences in group sizes and their effects are related to the so called group-size paradox, i.e. the observation that larger groups can be less successful than smaller groups in collective actions due to increased free-riding^20,55^. This paradox has been extensively studied for both within-group cooperation^85,86^ and between-group contests^87,88^. Here we considered the effects of group size on the group success in the context of more complex group interactions including both cooperation and competition. We have shown that differences in group sizes have two main structural effects. First, larger groups have more baseline resources making them potentially more powerful. Second, in larger groups individuals receive smaller shares of the collectively produced resources making cooperation more difficult because of increased free-riding. The strength of the latter effect declines with decreasing rivalrousness $\alpha$ of the goods. [When $\alpha$ is close to one, each individual’s share is inversely proportional to the group size. In contrast, if $\alpha$ is close to zero, the amount of resources received by each individual does not depend on its group’s size.] The interaction between the above two effects drives the dynamics of the model. For groups not involved in between-group cooperation, smaller size leads to more resources (and power) if the goods are rivalrous but less power and equal resources (compared to those produced by larger groups) if goods are non-rivalrous. Similar results were obtained for a related model in Ref.^56^. For groups involved in between-group cooperation, larger size most often leads to more resources and power for any degree of rivalrousness. Nevertheless with fully rivalrous goods, cooperation in very large groups breaks down and such group withdraw from between-group cooperation. This will also decrease their power.

There are a number of parallels between our model predictions and observations from real societies. Different stable states predicted in our model are similar to those found in some past and present societies. For example, some alliances of Middle East tribes^17^ and cooperative structures among Native American Nations^89^ can be viewed as examples of equilibria with a relatively egalitarian coalition. Conversely, confederations of Turco-Mongolin tribes with leading and subordinate tribes^17^ can be treated as an illustration of equilibria with high inequality between coalition members. The non-equilibrium dynamics (observed under some conditions in our model) has been a focus of recent research on historical societies^19,50^. The important role of social institutions, and checks and balances preserving cooperation (which we explicitly modeled here via parameter $\varepsilon$) is well appreciated in the social sciences. For example, Botswana has a very high per-capita growth rate compared to other African countries. This can be explained by strong institutions that existed in Tswana tribes in the pre-colonial period, aimed to maintain cooperation and resolve conflicts among them^18^. These institutions were preserved and reinforced during history because of a relatively small effect of colonialism on Tswana tribes, and due to the inclusive nature of these institutions with respect to other ethnic groups^90^. See also the example of the East Asia “tigers” briefly discussed above. Another interesting example related to our model are Pueblo societies, some of which were organized as a collection of interacting clans differing in power which depended on land-ownership and their role in religious rituals. The clans cooperated in the cultivation of the land but also competed for control over it^91^. Under good environmental conditions social relationships between clans were egalitarian and cooperative. However under bad conditions (e.g., rain or early frost) more powerful clans had food and stayed in the village while less powerful clans had to go out to hunt and gather. In our model, deterioration of environmental conditions can be captured by a decrease in the benefit parameter $B_2$. Decreasing $B_2$ decreases the size of the cooperative coalition if the incumbency parameter is relatively small, or the incumbency parameter and the benefit parameter $B_2$ are both intermediate (for more details see Figs. S20, S21 in the SM). This prediction is well in line with the anthropological observations mentioned above.

Our hope is that future work will allow for some predictions of our model to be tested using proxies and data collected from real societies. For example, the level of cooperation in the society can potentially be estimated using GDP-type measures. There are also a number of ways to measure horizontal inequality^12,92^, while the strength of checks and balances mechanisms can be estimated using data on the strength of democratic institutions and similar measures from the World Values Surveys^93^.

Our model has a number of limitations. First, because of within-group free-riding, the levels of within-group cooperation observed in our model are relatively low. In particular, only dominant groups can contain relatively high number of cooperating individuals. However there is a number of additional mechanisms potentially increasing within-group cooperation which we did not consider. These include reputation, punishment^21, 22,25,94,95^, between-individual differences^65^, and social norms^67,96,97^. Certain types of free-riding can be mitigated if individuals update their strategies by using imitation or foresight^25–27^ rather than the myopic best response we used here. We anticipate that some equilibria we observed would not occur with foresight. An example is a stable equilibrium where subordinate members of the coalition have lower power and payoffs than defecting groups. If a subordinate group takes into account future actions of its members, it may be beneficial for it to switch to defection. Although that would reduce its immediate payoff, the future payoffs will be higher. Second, here we dealt with well-defined and stable groups. However, the structure of social interactions can be much more complex. For example, interactions between individuals can be represented as a multilayer network^98, 99^, where each layer corresponds to a different type of interaction (e.g., social relationships, business collaborations, contacts via social networks). Third, groups can also face additional costs of building a coalition. These costs are implicitly embedded into our parameters (e.g., in the benefit parameter $B_2$). However, the model can be generalized to capture different forms of the coalition costs. Naturally, we anticipate that introducing additional costs will decrease the tendency of groups to cooperate. Fourth, here we assume that groups cooperate to produce some collective action as in “us versus nature” games^66^. However, many historic alliances were founded to win conflicts against other societies. Describing such interactions could be done within the framework of “us versus them” game^66^ at a new inter-society level. Fifth, the incumbency parameter by itself can be a multidimensional parameter rather than a scalar, or it can be formed endogenously as a response to changes in the focal social system. Finally, in our model individuals try to maximize their material payoffs. In real life situations, non-material factors (e.g., beliefs, conformity with group-members, injunctive norms or different rituals) can have a significant impact on individual decision-making^100,101^. It would be also good to find a way for scaling up group sizes perhaps using the mean field game theory methods^102^ as well as to add spatial structure which has been a focus of many studies of cooperation^103–105^. We leave these extensions and generalizations for future work.

Overall, our paper highlights the challenges of maintaining cooperation in complex societies and provides an additional argument towards the importance of social institutions aiming to prevent politically powerful groups from bending the rules of competition in their own favor.

## Methods

### A single group

Consider a single group of size *n*. Generalizing earlier results in Gavrilets and Shrestha^26^ we find that there will be a positive effort $X^*$ in the within-group game if $b_1>c(1+X_0),$ that is, if the maximum benefit of within-group cooperation $b_1=B_1/n^{\alpha }$ is sufficiently large relative to individual cost *c* and the group’s half-effort $X_0$. Let

9a $$\begin{aligned} R_1=B_1/(cX_0). \end{aligned}$$

be the ratio of the maximum group benefit $B_1$ to the group cost $cX_0$ at half-effort. Group effort $X^*$ at equilibrium increases with $R_1$ but naturally cannot exceed *n*. Specifically, $X^*$ can be approximated (see the SM) as

9b $$\begin{aligned} X^*= {\left\{ \begin{array}{ll} 0, \ \text {if}\ R_1 < n^{\alpha },\\ \min \bigg\{n, X_0 (\sqrt{\frac{R_1}{n^{\alpha }}}-1)\bigg\},\ \text {if}\ R_1 > n^{\alpha }. \end{array}\right. } \end{aligned}$$

In the SM we also show that if the goods produced are characterized by some rivalrousness (i.e., $\alpha >0$), then under some conditions smaller groups will make a larger effort than larger groups (Fig. S1). This is an example of Olson’s group-size paradox^20, 55–57^.

### Symmetric equilibria

Here we focus on the existence and stability to small perturbations in power of groups of symmetric equilibria with *C* cooperating groups which all are identical in their efforts and power (see section Some equilibria in the SM). Moreover, we assume that each group contains at least one cooperating individual. For equilibria with $C<G$, we will assume that $R_1/n^{\alpha }>1$ so that each non-cooperating group has individuals making a nonzero effort. In deriving our results we used an approximation which treats individual contributions $x_i$ as continuous. This approach is justified by results in Gavrilets and Shrestha^26^ as well as by the comparisons of our analytical with numerical results.

**Figure 8:**
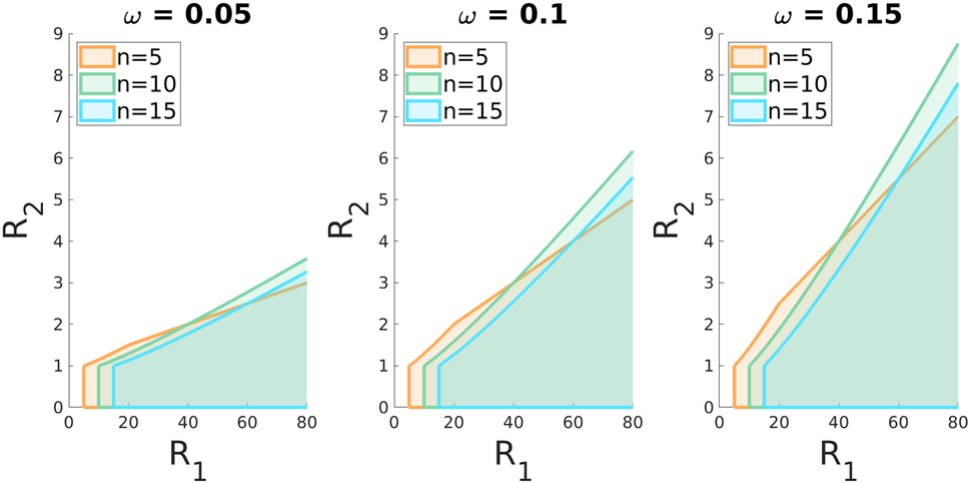
The equilibrium with no cooperating groups ($C=0$) exists and is locally stable when the benefit-to-cost ratios of the within-group game $R_1$ and between-group game $R_2$ lie in the corresponding shaded region for different group sizes *n* and parameter $\omega$. Other parameters: $X_0=5, \alpha =1$.

First, an equilibrium with no cooperating groups (with $C=0$) exists if each group would not be motivated to cooperate. This condition simplifies to

10a $$\begin{aligned} R_2<1+\omega R_1\left( 1-\sqrt{ \frac{n^{\alpha }}{R_1} }\right) , \end{aligned}$$

where

10b $$\begin{aligned} R_2=B_2/Z_0 \end{aligned}$$

is the benefit-to-cost ratio for the between-group game, and

10c $$\begin{aligned} \omega =\frac{cX_0}{Z_0} \end{aligned}$$

is the ratio of the costs of groups in within- and between-group games. Thus the state with no between-group cooperation exists only if the benefit-to-cost ratio $R_2$ of the between-group game is sufficiently small relative to that for the within-group game, $R_1$. The upper bound on $R_2$ declines with increasing the group size *n* and the degree of rivalrousness $\alpha$ but increases with parameters $\omega$ and $R_1$.

The above equilibrium is stable to small perturbations in groups’ power, if $\varepsilon <1$. For more details see the SM. Figure  8 illustrates the above results.

Second, consider an equilibrium with $C>0$ of identical cooperating groups. At this equilibrium, the effort of each cooperating group is

11 $$\begin{aligned} X_{c}^{*}= \frac{X_0}{\omega C R_1 +1}\left( \sqrt{\frac{R_1 R_2}{C n^{\alpha }}}-1\right) , \end{aligned}$$

which is positive, if $R_1R_2>C n^{\alpha }$. Thus $X_c^*$ decreases with increasing *n*, $\omega$, $\alpha$, $R_1$ and *C*. In contrast, $X_c^*$ increases with increasing $R_2$. The number of groups *G* has no effect on $X_c^*$.

**Figure 9:**
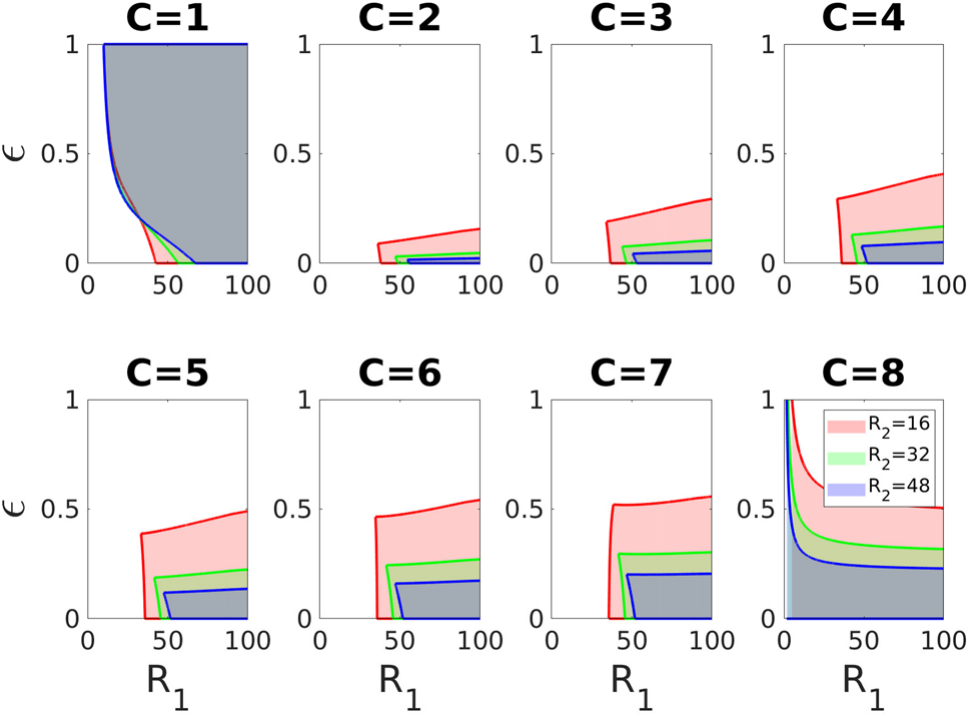
The equilibrium with $C\ (>0)$ equal cooperating groups exists and is locally stable when the benefit-to-cost ratio of the within-group game $R_1$ and the incumbency parameter $\varepsilon$ lie in the shaded regions for different values of $R_2$. Other parameters: $\omega =0.1$, $X_0=5$, $c=1$, $Z_0=50$, $\pi ^0=1$, $\alpha =1$, $G=8$ and $n=10$.

Consider a general case, when the total material payoff to a cooperating group is higher than the total material payoff to a defecting group (the opposite case is considered in Proposition 4 in the SM). An equilibrium with $C>0$ cooperating groups exists, if each cooperating group is not motivated to defect; and each non-cooperating group is not motivated to cooperate (if $C<G$). The former condition can be expanded to

$$\begin{aligned} R_1<n^{\alpha }\frac{CR_2}{4}\Bigg (1+\sqrt{1+\frac{4}{C^2 n^{\alpha } \omega }\Big (1-\frac{1}{R_2}\Big )}\Bigg )^2, \end{aligned}$$

i.e., a cooperating group will not be interested in withdrawing from cooperation, if the benefit-to-cost ratio $R_1$ for the within-group game is lower than a threshold which increases with increasing the benefit-to-cost ratio $R_2$ for the between-group game. For more details see section Some equilibria in the SM.

The latter condition can be written as

$$\begin{aligned} \varepsilon >\varepsilon _{min}, \end{aligned}$$

where the boundary $\varepsilon _{min}$ is defined implicitly by algebraic equations (for more details see section Some equilibria and Proposition 2 in the SM). The above equilibrium is stable to small perturbations in groups’ power, if

$$\begin{aligned} \varepsilon <\varepsilon _{max}, \end{aligned}$$

where the boundary $\varepsilon _{max}$ is defined implicitly by algebraic equations (for more details see section Some equilibria and Proposition 3 in the SM).

Overall, we conclude that an equilibrium with $C>0$ cooperating groups exists and is locally stable if the incumbency parameter is within a certain range:

$$\begin{aligned} \varepsilon \in {\left\{ \begin{array}{ll} (\varepsilon _{\min }, \varepsilon _{\max }), \text { if } C < G, \\ {[}0, \varepsilon _{\max }), \text { if } C=G. \end{array}\right. } \end{aligned}$$

Recall that the incumbency parameter $\varepsilon$ affects the level of inequality within the society (since for high values of the incumbency parameter $\varepsilon$ factions with higher power have more opportunities to bend the rules of competition in their favor). Intuitively, having $\varepsilon$ larger than a minimal value $\varepsilon _{min}$ prevents non-cooperating groups from joining the coalition (because they will have low power and thus will not receive enough of jointly produced resources). At the same time, having $\varepsilon$ smaller than a maximum value $\varepsilon _{max}$ prevents inequality growth within the coalition. Figure 9 illustrates these results (along with the results on the case when the total material payoff to a cooperating group is lower than the total material payoff to a defecting group).

In the case of complete between-group cooperation (i.e. with $C=G$)

12 $$\begin{aligned} \varepsilon _{\max }=\frac{A_c^*}{A_c^*+Q_G/G^2}, \end{aligned}$$

where $A_c^*=\Pi ^0-cX^*$ is the resource of each group before between-group game and $Q_G$ is the resource produced as a result of between-group cooperation. Thus increasing $Q_G$ or the group’s cost $cX^*$ decrease $\varepsilon _{\max }$ while increasing the number of groups *G* or group endowment $\pi ^0$ make $\varepsilon _{\max }$ larger. For more details see the SM.

## Supplementary information

Supplementary Information.

## Data Availability

Data and relevant code for this research work are stored in GitHub: dtverskoi/The-dynamics-of-cooperation-power-and-inequality-in-a-group-structured-society and have been archived within the Zenodo repository: https://doi.org/10.5281/zenodo.4247950.
