# Supplementary material for: The dynamics of cooperation, power, and inequality in a group-structured society
Source: Sci Rep. 2021 Sep 21;11:18670. doi: 10.1038/s41598-021-97863-7 (PMC8455579; doi:10.1038/s41598-021-97863-7)
Supplement: Supplementary file 1 — Supplementary Information. [file 41598_2021_97863_MOESM1_ESM.pdf]

Supplementary materials for:

The dynamics of cooperation, power, and inequality in a group-structured society

Denis Tverskoi, Athmanathan Senthilnathan and Sergey Gavrilets

- Table S1 lists model variables, functions, parameters and statistics.
- Part 1 provides more detailed analytical results on the existence and local stability of symmetric equilibria.
- Part 2 provides more results of numerical simulations underlying our conclusions presented in the main text. Figures are arranged in the order of subsections of the main text.

Contents

1 Part 1. Supplementary modeling **3**
1.1 Best response . . . 3
1.2 Some equilibria . . . 4
1.3 Propositions and proofs . . . 8

2 Part 2. Additional simulation results **15**
2.1 Groups with identical sizes . . . 15
2.1.1 Equilibria . . . 15
2.1.2 Non-equilibrium dynamics . . . 21
2.1.3 Effects of parameters . . . 22
2.2 Groups with different sizes . . . 33
2.2.1 Effects of parameters, rivalrous good . . . 33
2.2.2 Effects of parameters, non-rivalrous goods . . . 36
2.2.3 Effects of group size, rivalrous goods . . . 38
2.2.4 Effects of group size, non-rivalrous goods . . . 46
2.2.5 Non-equilibrium dynamics . . . 52

Table S1: Model variables, functions, parameters and statistics.

	Symbols	Their meaning
Variables	x_{ij}	effort of individual i in group j ($x_i = 0$ or $x_i = 1$)
	θ_j	group's cooperation status i.e., "elites" ($\theta_j = 1$) or "counter-elites" ($\theta_j = 0$)
	f_j	group power ($0 \leq f_j \leq 1, \sum_{j=1}^{G} f_j = 1$)
Functions	X_j	number of cooperating individuals in group j, $X_j = \sum_{i=1}^{n_j} x_{ij}$
	$P_j(X)$	production function at within-group level, $P_j = B_1 \frac{X_j}{X_j + X_0}$
	Z	total contribution of all cooperating groups, $Z = \sum_{j=1}^{G} \theta_j P_j$
	$Q(Z)$	production function at between-group level, $Q = B_2 \frac{Z}{Z + Z_0}$
	v_j	group's share of the collective good, $v_j = \frac{\theta_j f_j}{\sum_{k=1}^{G} \theta_k f_k}$
	Π_j^0	group's endowment, $\Pi_j^0 = \pi^0 n_j$
	Π_j	group payoff, $\Pi_j = \Pi_j^0 - cX_j + (1 - \theta_j) P_j + v_j Q$
	π_{ij}	individual payoff, $\pi_{ij} = \pi^0 - cx_{ij} + (1 - \theta_j) P_j / n_j^{\alpha} + v_j Q / n_j^{\alpha}$
	y_j	group effort in the political game, $y_j = \Pi_j (1 - \varepsilon + \varepsilon f_j)$
Parameters	n_j, G	number of individuals in group j and the number of groups
	π^0, c	individual endowment and the cost of contribution
	B_1	benefit parameter of within-group cooperation
	B_2	benefit parameter of between-group cooperation
	X_0, Z_0	half-effort parameters of functions $P_j(X)$ and $Q(Z)$, respectively
	ε	incumbency parameter
	α	degree of rivalrousness of the produced goods
	λ	precision parameter in the QRE approach [1]
	μ_1, μ_2	update probabilities of individuals and groups
Statistics	C	the number of cooperating groups
	I	Gini index of inequality among cooperating groups
	σ	standard deviation of efforts among cooperating groups
	k_{NE}	number of simulations with non-equilibrium dynamics

1 Part 1. Supplementary modeling

1.1 Best response

In our models, individuals decide whether or not to contribute an unit effort to the group production. However below in analyzing their best-response actions we will approximately treat their efforts as continuous. This approach is justified by the results of Gavrilets & Shrestha (2019) [2] who showed that the two approaches make similar predictions.

Best response for individuals from a single (or non-cooperating) group. Dropping the subscripts specifying the individual and its group, the payoff to an individual making effort x from a non-cooperating group is

$$\pi = \pi^0 - cx + \frac{1}{n^\alpha} P,$$

where $P = B_1 \frac{X}{X+X_0}$ and $X = \sum x$. The best response can be found by solving the first order condition $d\pi/dx = 0$. The resulting normalized group effort is

$$X^*/X_0 = \sqrt{R_1/n^\alpha} - 1, \tag{S1a}$$

where

$$R_1 = B_1/(cX_0) \tag{S1b}$$

is the benefit-to-cost ratio for the within-group game. Naturally this solution makes sense only if

$$R_1 > n^\alpha.$$

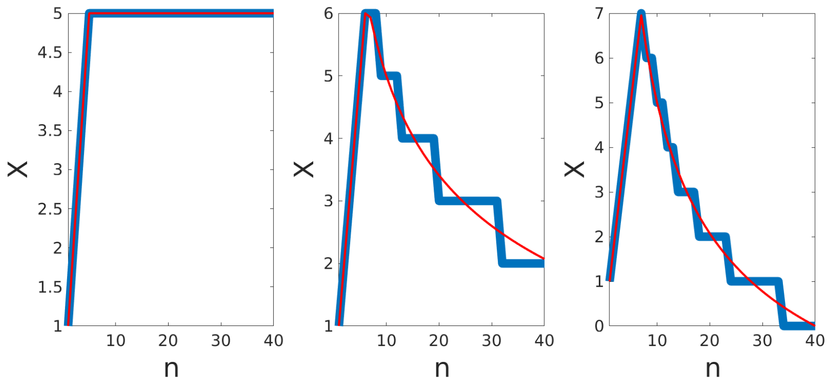

Figure S1: The best response effort X^* of a single group calculated from numerical simulations (blue lines), and analytical approximations (red lines). Parameter $\alpha = 0, 0.5$ and 1 in the left, middle and right panels, respectively. Baseline parameters: $c = 1$, $X_0 = 5$ and $B_1 = 20 \cdot 10^\alpha$. For this choice of parameter values and with small n, all group members cooperate so that the group effort increases linearly with n.

The right-hand side of the above inequality changes from 1 (for non-rivalrous games, $\alpha = 0$) to n (for rivalrous games, $\alpha = 1$). With individual efforts equal to just 0 and 1 the group effort X^* cannot exceed the group size. The dependence between X^* and the group size n is illustrated in Figure S1. The equilibrium production P^* can be found from the above equations in a straightforward way.

Note that in the case of non-rivalrous games (i.e. if $\alpha = 0$), the group effort X^* does not depend on the group size n provided the predicted value of X^* is smaller than n. In contrast, in similar games where individual strategies are defined as probabilities p of contributing to a public goods production, the corresponding Nash equilibrium p_N can exhibit a nonlinear dependence on n (see

the SM in [2] and also [3].)

Best response for individuals from a cooperating group. The payoff to an individual making effort x from a cooperating group is

$$\pi = \pi^0 - cx + \frac{1}{n^\alpha}\, v\, Q,$$

where $Q = B_2 \frac{Z}{Z+Z_0}$, $Z = \sum P$, and we drop the subscripts specifying the individual and its group. The best response function can be found by solving the first order condition $d\pi/dx = 0$.

Best response for a group. The focal group will contribute to the between-group cooperation if it expects that this would result in a higher payoff than if defecting, i.e. if

$$vQ > P.$$

The dynamics of power. The group j power at the next time step is given by

$$f_j' = \begin{cases} \frac{\Pi_j(1-\varepsilon+\varepsilon f_j)}{\sum \Pi_k(1-\varepsilon+\varepsilon f_k)}, & \text{if } \sum \Pi_k(1-\varepsilon+\varepsilon f_k) > 0 \\ \frac{1}{G}, & \text{otherwise,} \end{cases} \tag{S2}$$

where Π_j is the total material payoff obtained by group j.

1.2 Some equilibria

Here we summarize analytical results on the existence and stability of several equilibria characterized by a high degree of symmetry. In particular, we will consider equilibria with C groups contributing to between-group cooperation and $G - C$ groups defecting. Within each of these two classes, individual groups will be identical with respect to their size, effort, payoff, and power. Note that our numerical results discussed in the main text show that there are also other types of equilibria.

An equilibrium with no cooperating groups ($C = 0$). Consider a symmetric state where each group has an equal power $f = 1/G$ and makes a positive effort X^* which leads to production $P^* = B_1 \frac{X^*}{X^*+X_0}$. (This requires $R_1 > n^\alpha$, see eq. S1a.) Let none of them participate in the between-group game. If a group were to change its strategy and contribute P^* to the between-group game, its expected payoff would be $Q = B_2 P^*/(P^* + Z_0)$. The group would not be motivated to do it if

$$P^* > Q. \tag{S3a}$$

The last conditions simplifies to

$$R_2 < 1 + \omega R_1 \left(1 - \sqrt{\frac{n^\alpha}{R_1}}\right), \tag{S3b}$$

where

$$R_2 = B_2/Z_0 \tag{S3c}$$

is the benefit-to-cost ratio for the between-group game, and

$$\omega = \frac{cX_0}{Z_0} \tag{S3d}$$

is the ratio of the costs of groups in within- and between-group games. We conclude that the feasibility of the equilibrium with $C = 0$ requires that the benefit-to-cost ratio R_2 of the between-group game is sufficiently small relative to that for the within-group game. The critical value of R_2 declines with increasing the group size n and the degree of rivalrousness α but increases with parameters ω and R_1.

We also need to consider whether the equilibrium is stable to perturbations in groups' power. The dynamics of power are described by the system (S2) of G difference equations of which only $G-1$ are independent. Directly computing the derivatives, we find that the corresponding Jacobian matrix is diagonal and each element on the diagonal is equal to

$$\frac{\varepsilon}{G(1-\varepsilon)+\varepsilon},$$

which is always smaller than 1 if $\varepsilon < 1$. Therefore, small perturbations of power will decay, if

$$\varepsilon < 1. \tag{S4}$$

Figure 1 in the Main text illustrates these results.

Equilibria with $1 \leq C \leq G$ **cooperating groups**. Consider a symmetric equilibrium state where $C > 0$ groups participate in between-group game and $G - C$ groups do not participate in it. Let X_c^*, P_c^* and f_c^* be the best response effort, the corresponding production, and power of each of the cooperating groups. Using some algebra one shows that the best response effort X_c^* of a cooperating group is

$$X_c^*/X_0 = \frac{1}{\omega C R_1 + 1}\left(\sqrt{\frac{R_1 R_2}{C n^\alpha}} - 1\right). \tag{S5}$$

Naturally this solution makes sense only if $R_1 R_2 > C n^\alpha$. Also, with individual efforts equal to just 0 and 1 the group effort X_c^* cannot exceed the group size. The corresponding equilibrium production P_c^* can be found from Equation S5 and Equation 1 in the Main text in a straightforward way. One concludes that X_c^* decreases with increasing n, ω, α, R_1 and C. In contrast X_c^* increases with increasing R_2. The number of groups G has no effect on X_c^*. Figure S2 illustrates these results.

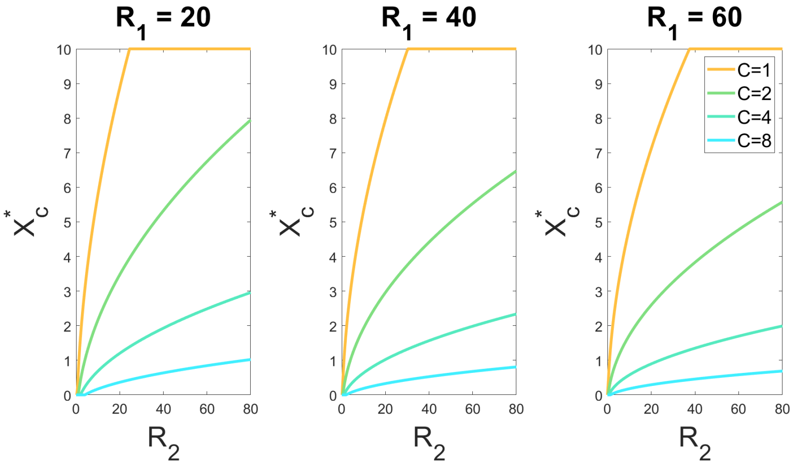

Figure S2: The best response effort X_c^* of a cooperating group in a society with $C > 0$ cooperating groups. Baseline parameters: $\omega = 0.1$, $X_0 = 5$, $n = 10$ and $\alpha = 1$.

Three conditions must be satisfied for this state to be locally stable. First, cooperating groups

should not be motivated to defect. Second, defecting groups should not be motivated to start cooperating (naturally, this condition is irrelevant if $C = G$, i.e. if all groups cooperate). Third, the state should be stable to small fluctuations in the power of groups. We will consider these three conditions separately. Later we provide the results for the general case where a cooperative group is more successful than a non-cooperative one (i.e., $\Pi_c^* \geq \Pi_d^*$). For a detailed description of the case where $\Pi_c^* < \Pi_d^*$ see Proposition 4.

1) The production resulting from cooperation of C groups is

$$Q_C = B_2 \frac{CP_c^*}{CP_c^* + Z_0}.$$

A cooperating group will not withdraw from cooperation if

$$P_c^* < \frac{1}{C} Q_C. \tag{S6}$$

The above inequality can be expanded to (see Proposition 1)

$$R_1 < n^{\alpha} \frac{CR_2}{4} \left(1 + \sqrt{1 + \frac{4}{C^2 n^{\alpha} \omega} \Big(1 - \frac{1}{R_2}\Big)} \right)^2,$$

i.e., a cooperating group will not be interested in withdrawing from cooperation, if the benefit-to-cost ratio R_1 for the within-group game is lower than a threshold which increases with increasing the benefit-to-cost ratio R_2 for the between-group game (i.e., benefits from the between group game are high enough compared to benefits from the within group game to motivate a cooperating group to cooperate). The above threshold decreases with the ratio of costs ω growth and with a decrease in n^{α} (if $\alpha \neq 0$). The effect of the number of cooperating groups is more complicated: the threshold decreases with an increase in C if $R_2(4 - 3C^2\omega n^{\alpha}) \leq 4$; and increases with an increase in C otherwise.

2) If a defecting group with production P_d^* and power f_d^* were to join a coalition of C cooperating groups, the joint production would become

$$Q_{C+1} = B_2 \; \frac{P_d^* + CP_c^*}{P_d^* + CP_c^* + Z_0}.$$

The share of the production going to this group would be

$$v = \frac{f_d^*}{f_d^* + Cf_c^*}.$$

Then a non-cooperating group will not start cooperating if

$$P_d^* > vQ_{C+1}. \tag{S7a}$$

Since $Cf_c^* + (G - C)f_d^* = 1$, we can rewrite inequality (S7a) as

$$f_c^* > \frac{1}{C} \frac{(Q_{C+1} - P_d^*)}{(Q_{C+1} - P_d^*) + (G - C)P_c^*}, \tag{S7b}$$

i.e., the power of each cooperating group should be sufficiently high. High power of cooperating groups entails low power of a defecting group which in turn implies that if the latter were to

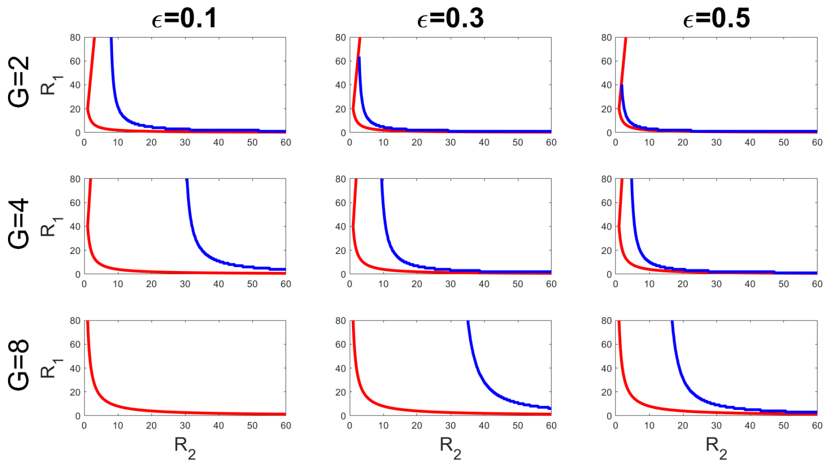

Figure S3: Existence and local stability of equilibria with $C = G$ equal cooperating groups. The blue curves are described by equation (S11). The red curves are described by equations (S12). The equilibrium exists and is locally stable for parameter values between the red and blue curves. Baseline parameters: $\omega = 0.1$, $X_0 = 5$, $c = 1$, $\pi^0 = 1$, $\alpha = 1$ and $n = 10$.

cooperate, it will obtain very small share of the resource produced as a result of between-group cooperation. From the dynamic equations for power (S2), we know that at equilibrium

$$\frac{f_d^*}{f_c^*} = \frac{\Pi_c^*}{\Pi_d^*}\,\frac{1-\varepsilon+\varepsilon f_d^*}{1-\varepsilon+\varepsilon f_c^*}.$$

where Π_c^* and Π_d^* are the equilibrium payoffs to cooperating and defecting groups. Eliminating f_d^*, we find that f_c^* satisfies to a quadratic equation

$$\varepsilon C (f_c^*)^2 \Delta + [\Delta(C - C\varepsilon - \varepsilon) + G(1-\varepsilon)\Pi_d^*] f_c^* - \Pi_c^*(1-\varepsilon) = 0, \tag{S8}$$

where $\Delta = \Pi_c^* - \Pi_d^*$ is the difference in payoffs between two types of groups at equilibrium. This quadratic has a single root between 0 and $1/C$ which depends on ε. Moreover, f_c^* is an increasing function of ε (see Proposition 2 below).

If $\varepsilon = 1$, the only relevant root is $f_c^* = 1/C$ and the condition (S7b) is satisfied. On the other hand if $\varepsilon = 0$, then $1/f_c^* = C + G\frac{\Pi_d^*}{\Pi_c^*}$. If $Q_{C+1} \leq P_d^*$, the condition (S7b) is satisfied. With $Q_{C+1} > P_d^*$, the condition (S7b) is satisfied if

$$\frac{1}{C}\,\frac{\Pi_d^*}{\Pi_c^*} \leq \frac{P_d^*}{Q_{C+1} - P_d^*}. \tag{S9}$$

Since f_c^* is an increasing function of ε, the condition (S7b) will be satisfied for each $\varepsilon \in [0, 1]$, if $Q_{C+1} > P_d^*$ and the condition (S9) holds; or if $Q_{C+1} \leq P_d^*$. Otherwise the condition (S7b) can be rewritten as

$$\varepsilon > \varepsilon_{min},$$

where ε_{min} is a positive threshold (see Proposition 2). This means that the existence of the equilibrium with $1 < C < G$ cooperating groups requires ε to be sufficiently high to create a barrier for non-cooperating groups from switching to cooperation. That is, democratic checks and balances must be weak enough so that they cannot prevent the monopolization of power by an existing cooperative coalition.

3) Finally we need to consider if the equilibrium is stable to small perturbations in groups' power. The dynamics of power are described by G difference equations (S2) of which only $G-1$ are independent. Directly computing the corresponding Jacobian matrix and its spectrum we end up with the following result (see Proposition 3). A perturbation in power will decay if

$$\varepsilon < \varepsilon_{\max}, \tag{S10}$$

where $\varepsilon_{\max}$ is the unique solution to the following algebraic equation:

$$\varepsilon_{\max} = \frac{A_c^*}{A_c^* + (Q_C/C) f_c^*(\varepsilon_{\max})},$$

where $A_c^* = \Pi^0 - cX_c^*$ is the resource of cooperating group before receiving the reward of production and $f_c^*(\varepsilon_{\max})$ is the power of a cooperating group (which depends on ε) Note that $0 < \varepsilon_{\max} < 1$. The condition (S10) implies that ε should be sufficiently small to prevent the growth of inequality within the cooperative coalition.

The condition (S10) can be used to explain why the above equilibria are not observed in the group-based model (GBM) in Gavrilets et al. (2021) [4]. GBM can be recovered from our model if one assumes that $X_0 = 0$ and fixes actions of all individuals in all groups at $x = 1$. For small values of the incumbency parameter, the GBM is characterized by a non-equilibrium continuous turnover of dominant groups, while our model mostly exhibits equilibrium dynamics with a number of locally stable equilibria characterized by non-zero numbers of groups choosing to cooperate. An explanation of this fact is drawn on condition (S10). In the GBM, the endowment of a group after the within-groups interaction A_c^* is zero which entails that equilibria with C groups ($C > 1$) contributing to between-group cooperation are not stable, whereas such the endowment in our model is positive and, hence, the above equilibria become locally stable.

In the case of complete cooperation ($C = G$), ε_{max} can be found explicitly:

$$\varepsilon_{\max} = \frac{A_c^*}{A_c^* + Q_G/G^2}. \tag{S11}$$

This condition generalizes that in Gavrilets et al. (2021) [4] for the case of an arbitrary number of groups. We conclude that incumbency parameter ε should be lower than a certain threshold (i.e., $\varepsilon < \varepsilon_{max}$) in order to prevent an emergence of inequality within the cooperating coalition.

Overall the stability of the equilibrium requires the incumbency parameter to be within certain bounds: ε should be high enough to make it unprofitable for outsiders to join the coalition and, at the same time, ε should be small enough to prevent the growth of inequality within the coalition to stop defection.

The conditions for the existence and stability for the equilibrium with $C = G$ are illustrated in Figures S3 - S4 and Figure 2 in the Main text. Figure S3 illustrates our results for the case of $C = G$ only while Figures 2 and S4 do the same for the case of $C < G$ as well.

1.3 Propositions and proofs

Proposition 1. Consider an equilibrium with $1 \leq C \leq G$ equal groups contributing to the between-group game. Let $0 < X_c^* < n$ be a group effort of a cooperating group, and $0 < X_d^* < n$ be a group effort of a defecting group (if $C < G$). X_c^* can be found from Equation S5, and X_d^* can be found from Equation S1a. Necessary conditions for the existence of the above equilibrium are:

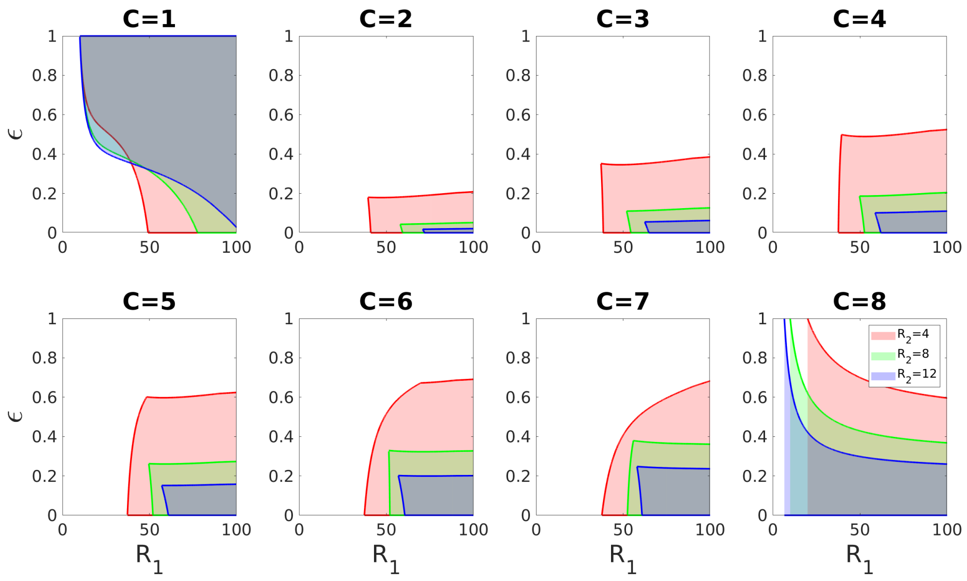

Figure S4: Existence and local stability of equilibria with C equal cooperating groups. The equilibrium with $C > 0$ equal cooperating groups exists and is locally stable when the benefit to cost ratio of the within-group game R_1 and the incumbency parameter ε lies in the shaded regions. Baseline parameters: $\omega = 0.025$, $X_0 = 5$, $c = 1$, $Z_0 = 200$, $\pi^0 = 1$, $\alpha = 1$, $G = 8$ and $n = 10$.

$R_2 > 1$ and $R_1 \in (M_l(R_2, C), M_h(R_2, C))$, where

$$M_l(R_2, C) = \begin{cases} Cn^\alpha / R_2, \text{ if } R_2 \in (1, \xi(C)), \\ n^\alpha, \text{ if } R_2 \geq \xi(C), \end{cases} \tag{S12a}$$

$$M_h(R_2, C) = n^\alpha \frac{CR_2}{4}\left(1 + \sqrt{1 + \frac{4}{C^2 \omega n^\alpha}\left(1 - \frac{1}{R_2}\right)}\right)^2, \tag{S12b}$$

and

$$\xi(C) = \begin{cases} C, \text{ if } C < G, \\ \infty, \text{ otherwise.} \end{cases} \tag{S12c}$$

Proof. The above equilibrium exists if (1) $P_d^* > 0$ (for $C < G$); (2) $P_c^* > 0$; (3) a cooperating group has no incentives to change its decision, whereas all other groups adhere to their strategies; and (4) a defector has no incentives to change its decisions, while all other groups adhere to their strategies (for $C < G$). Here we will analyse only the first three conditions:

(1) $P_d^* > 0$ that entails $R_1 > n^\alpha$;

(2) $P_c^* > 0$ implies that $R_1 R_2 > Cn^\alpha$;

(3) According to Inequality S6 we deduce that $P_c^* < \frac{(R_2 - 1)Z_0}{C}$.

The last condition can be transformed into $C\omega R_1 - C\omega\sqrt{Cn^\alpha R_1 R_2} + 1 - R_2 < 0$. It means that $H_-(R_2) < R_1 < H_+(R_2)$ and $R_2 > \frac{4}{C^2 \omega n^\alpha + 4}$, where

$$H_+(R_2) = n^\alpha \frac{CR_2}{4}\left(1 + \sqrt{1 + \frac{4}{C^2 \omega n^\alpha}\left(1 - \frac{1}{R_2}\right)}\right)^2, \text{ if } R_2 \geq \frac{4}{4 + C^2 \omega n^\alpha},$$

$$H_-(R_2) = \begin{cases} 0, \text{ if } \frac{4}{4+C^2\omega n^\alpha} \leq R_2 < 1, \\ n^\alpha \frac{CR_2}{4}\left(1 - \sqrt{1 + \frac{4}{C^2\omega n^\alpha}\left(1 - \frac{1}{R_2}\right)}\right)^2, \text{ otherwise.} \end{cases}$$

The function $H_-(R_2)$ is monotonically decreasing on the set $\left[\frac{4}{4+C^2\omega n^\alpha}, 1\right]$, whereas the function $H_+(R_2)$ is monotonically increasing on its domain. Let us define a function $H_0(R_2) = Cn^\alpha/R_2$, which is monotonically decreasing on $R_2 \in (0, \infty)$. According to monotonicity it's graph has a unique intersection point $(1, Cn^\alpha)$ with the graph of the function $H_+(R_2)$. As far as $H_-(1) = 0$, we conclude that $H_-(R_2) < H_0(R_2)$ for all $R_2 \geq 1$. Consequently, conditions (2) and (3) can be rewritten in the following way: $R_2 > 1$ and $R_1 \in (Cn^\alpha/R_2, H_+(R_2))$. If $C = G$, the proposition is proved. Otherwise, we need to consider condition (1) as well. Taking into account condition (1) we end up with the following result for each $C \in \{1, .., G-1\}$: $R_1 \in (Cn^\alpha/R_2, H_+(R_2))$, if $R_2 \in (1, C)$; and $R_1 \in (n^\alpha, H_+(R_2))$, if $R_2 \geq C$. Folding together the results for $C = G$ and $C < G$ we obtain that $R_2 > 1$ and $R_1 \in (M_l(R_2, C), M_h(R_2, C))$. The proposition is proved.

Proposition 2. Consider an equilibrium with $1 \leq C < G$ equal groups contributing to the between-group game. Assume also that each group produce a non-zero effort in the within-group game. Let $Q_{C+1} > P_d^*$, the condition (S9) does not hold and $\Pi_c^* \geq \Pi_d^*$, where Π_c^* and Π_d^* are payoffs to cooperating and defecting groups. Then, a necessary condition for the existence of the above equilibrium is

$$\varepsilon > \varepsilon_{min},$$

where $\varepsilon_{min} > 0$ is defined below.

Proof. Assume that the above equilibrium exists. Let X_c^*, P_c^*, Π_c^*, f_c^* and X_d^*, P_d^*, Π_d^* be equilibrium characteristics of cooperating and non-cooperating groups respectively. Then, a non-cooperating group will not be interested in starting to cooperate, which implies that the condition (S7a) holds. The above condition can be rewritten as inequality (S7a). Note that the right side of the above inequality is less than $1/C$.

We claim that f_c^* is a continuous and monotonically increasing function of the parameter $\varepsilon \in [0, 1]$ and it's image is the set $Im(F_c^*) = \left[\frac{\Pi_c^*}{C\Pi_c^* + (G-C)\Pi_d^*}, 1/C\right]$. Moreover, since the condition (S9) does not hold, we deduce that

$$\varepsilon > f_c^{*-1}\Big(\frac{1}{C}\frac{(Q_{C+1} - P_d^*)}{(Q_{C+1} - P_d^*) + (G-C)P_d^*}\Big) > 0, \tag{S13}$$

Recalling $\varepsilon_{min} = f_c^{*-1}\Big(\frac{1}{C}\frac{(Q_{C+1} - P_d^*)}{(Q_{C+1} - P_d^*) + (G-C)P_d^*}\Big)$ we end up with the statement of the proposition. Therefore to finish the proof we need to show that f_c^* is a monotonically increasing function of ε and $Im(f_c^*) = \left[\frac{\Pi_c^*}{C\Pi_c^* + (G-C)\Pi_d^*}, 1/C\right]$.

As it has been shown before, f_c^* satisfies a quadratic equation (S8). It is not a straightforward task to prove monotonicity of f_c^* by solving the above equation and calculating the derivative of the resulting expression explicitly. Therefore we suggest the following implicit arguments.

Consider a family of functions parametrized by ε: $\Big\{L_\varepsilon\Big\}_{\varepsilon \in [0,1]}$, where $L_\varepsilon = \varepsilon C f_c^2 \Delta + (\Delta(C - C\varepsilon - \varepsilon) + G(1-\varepsilon)\Pi_d^*)f_c - \Pi_c^*(1-\varepsilon)$ and $\Delta = \Pi_c^* - \Pi_d^*$. For each $\varepsilon \in [0, 1]$, $f_c^*(\varepsilon)$ can be found as the unique zero of L_ε on the set $(0, 1]$.

First, we claim that there exists the unique intersection point $(f_c^l, L(f_c^l))$ of functions L_{ε_1} and L_{ε_2}, $\varepsilon_1 \neq \varepsilon_2$ such that $f_c^l \in [0, 1]$. Moreover, we claim that this point is the same for all pairs

of functions, i.e., each function from $\left\{L_\varepsilon\right\}_{\varepsilon\in[0,1]}$ passes through the point $(f_c^l, L(f_c^l))$. Finally, we assert that $f_c^l \in (0, 1/C)$. Indeed, let's show that $0 < f_c^l < 1/C$. To do this note that f_c^l is the unique solution to the equation $L^0(f_c) = 0$, where

$$L^0(f_c) = Cf_c^2\Delta - ((C+1)\Delta + G\Pi_d^*)f_c + \Pi_c^*. \tag{S14}$$

Since $L^0(1/C) = -\frac{(G-C)\Pi_d^*}{C} < 0$ and $L^0(0) = \Pi_c^* > 0$, applying Intermediate value theorem we deduce that $0 < f_c^l < 1/C$.

Second, we assert that $L(f_c^l) < 0$ which entails that $\forall\varepsilon \in [0,1]$: $f_c^*(\varepsilon) \in (f_c^l, 1]$. To show this, one can calculate $L(f_c^l)$ directly: $L(f_c^l) = (C\Delta + G\Pi_d^*)f_c^l - \Pi_c^* = Cf_c^l\Delta(f_c^l - 1/C)$. As far as $f_c^l < 1/C$, one conclude that $L(f_c^l) < 0$. Since for each $\varepsilon \in [0,1]$: $L_\varepsilon(f_c^l) < 0$, $L_\varepsilon(1) \geq 0$ and $f_c^*(\varepsilon)$ is the unique solution to the equation $L_\varepsilon(f_c) = 0$ on $(0,1]$, one deduce that $\forall\varepsilon \in [0,1]$: $f_c^*(\varepsilon) \in (f_c^l, 1]$ (according to Intermediate value theorem).

Consider $\varepsilon_1, \varepsilon_2 \in [0,1]$ and assume that $\varepsilon_1 < \varepsilon_2$. We introduce a function $\delta(f_c) = L_{\varepsilon_2}(f_c) - L_{\varepsilon_1}(f_c)$. We claim that $\forall f_c \in (f_c^l, 1]$: $\delta(f_c) < 0$. Indeed, suppose that there exists $f_c' \in (f_c^l, 1]$ such that $\delta(f_c') \geq 0$. If $\delta(f_c') = 0$ we get a contradiction immediately. Let $\delta(f_c') > 0$. Since $\delta(1) = (G-1)\Pi_d^*(\varepsilon_1 - \varepsilon_2) < 0$, employing Intermediate value theorem we conclude that $\exists f_c'' \in (f_c^l, 1)$: $\delta(f_c'') = 0$, which in turn means that we get a contradiction.

Suppose by way of contradiction that $f_c^*(\varepsilon_1) \geq f_c^*(\varepsilon_2)$. In particular, it implies that $\delta(f_c^*(\varepsilon_2)) \geq 0$, which leads to a contradiction. Hence, f_c^* is a monotonically increasing function of ε as was to be shown. Moreover, f_c^* is a continuous function of ε, which entails $Im(f_c^*) = [f_c^*(0), f_c^*(1)]$, i.e. $Im(f_c^*) = \left[\frac{\Pi_c^*}{C\Pi_c^* + (G-C)\Pi_d^*}, 1/C\right]$. Proposition is proved.

Proposition 3. Assume that in equilibrium $1 < C \leq G$ equal groups contribute to the between-group game. Assume also that each group produces a non-zero effort in the within-group game, $\Pi_c^* \geq \Pi_d^*$ and $\pi^0 \geq c$. Then, a necessary condition for the above equilibrium to be locally stable is:

$$\varepsilon \leq \varepsilon_{max}, \tag{S15a}$$

where $0 < \varepsilon_{max} < 1$ can be found from the equation

$$\varepsilon_{max} = \frac{A_c^*}{A_c^* + f_c^* Q_C/C}, \tag{S15b}$$

and X_c^*, P_c^*, f_c^*, $A_c^* = \Pi^0 - cX_c^*$, and X_d^*, P_d^*, $A_d^* = \Pi^0 - cX_d^* + P_d^*$ are equilibrium characteristics of cooperating and defecting groups respectively.

Proof. The dynamics of power are described by G difference equations (S2) of which only $G-1$ are independent. Without loss off generality presume that among this $G-1$ groups first $C-1$ groups cooperate and other $G-C$ defect. Directly computing the derivatives, we find out that the corresponding Jacobian matrix calculated at the equilibrium point is

$$J = \begin{pmatrix} \nu I_{C-1} & fE \\ O & \mathfrak{D} \end{pmatrix}, \tag{S16}$$

where I_{C-1} is the identity matrix of size $(C-1)$, E is $(C-1)\times(G-C)$ matrix of ones; O is $(G-C)\times(C-1)$ zero matrix; and $\mathfrak{D}$ is $(G-C)\times(G-C)$ matrix; $\nu = \frac{\varepsilon A_c^* f_c^* + (1-\varepsilon)Q_C/C + 2\varepsilon Q_C f_c^*/C}{(A_c^* + Q_C/C)(1-\varepsilon+\varepsilon f_c^*)}$ and f are real numbers. Then the characteristic polynomial of J can be found as:

$$det(J - \lambda I_{G-1}) = det(\nu I_{C-1} - \lambda I_{C-1}) \cdot \det(\mathfrak{D} - \lambda I_{G-C}) = 0,$$

Immediately we conclude that J has an eigenvalue ν of multiplicity $C-1$. To find the rest of the spectrum (in the case of $C<G$) consider matrix $\mathfrak{D}$ in details. Using simple algebra one shows that the above matrix has the following form:

$$\mathfrak{D}_{j,m\in\{1,..,G-C\}} = \delta_{jm}\psi + \phi, \tag{S17}$$

where δ_{jm} is the Kronecker delta, $\psi = \frac{\varepsilon A_d^* f_c^*}{(A_c^*+Q_C/C)(1-\varepsilon+\varepsilon f_c^*)}$ and $\phi = -\frac{\varepsilon f_c^*(1-Cf_c^*)(A_d^*-A_c^*-Q_C/C)}{(G-C)(A_c^*+Q_C/C)(1-\varepsilon+\varepsilon f_c^*)}$. The characteristic polynomial of the above matrix satisfies the following difference equation:

$$det(\mathfrak{D}-\lambda I_{G-C}) = (\psi-\lambda)det(\mathfrak{D}-\lambda I_{G-C-1}) + \phi(\psi-\lambda)^{G-C-1}. \tag{S18}$$

The solution of the above equation has a form:

$$det(\mathfrak{D}-\lambda I_{G-C}) = (\psi-\lambda)^{G-C} + \phi(G-C)(\psi-\lambda)^{G-C-1}. \tag{S19}$$

We end up with two distinct eigenvalues of the matrix $\mathfrak{D}$ (the former one has the multiplicity equals to $(G-C-1)$ whereas the latter one represents a simple eigenvalue), if $C<G-1$:

$$\lambda_1 = \psi, \lambda_2 = \psi + \phi(G-C). \tag{S20}$$

and one eigenvalue λ_2, if $C=G-1$. As a result we derive that the matrix J has:

- three distinct eigenvalues

$$\lambda_1 = \psi, \lambda_2 = \psi + \phi(G-C), \lambda_3 = \nu, \tag{S21}$$

  with multiplicities $G-C-1$, 1 and $C-1$ respectively, if $2 \le C \le G-2$;

- two distinct eigenvalues

$$\lambda_2 = \psi + \phi(G-C), \lambda_3 = \nu, \tag{S22}$$

  with multiplicities 1 and $C-1$ respectively, if $2 \le C = G-1$;

- one distinct eigenvalue

$$\lambda_3 = \nu, \tag{S23}$$

  with multiplicity $C-1$, if $2 \le C = G$;

i.e. we have described all cases.

Since the above eigenvalues are non-negative, we need to compare the eigenvalues with 1 to check the above equilibrium for local stability. Since $\Pi_c^* \ge \Pi_d^*$, one can show that $\lambda_1, \lambda_2 < 1$. The condition $\lambda_3 \le 1$ can be represented in the following way:

$$\frac{\varepsilon f_c^* Q_C}{C} + \varepsilon A_c^* \le A_c^*. \tag{S24}$$

According to Proposition 2, F_c^* is an increasing function of ε, which entails $\varepsilon \le \varepsilon_{max}$, where ε_{max} can be found from Equation S15b.

To show that $0 < \varepsilon_{max} < 1$, consider inequality (S24) again. For $\varepsilon = 0$ we get $0 < A_c^*$ which is true for $r_0 > c$. For $\varepsilon = 1$ we obtain $(Q_C/C)f_c^*(1) < 0$, which is not true. As a result (since $f_c^*(\varepsilon)$ is continuous and following Intermediate value theorem), we prove that $\varepsilon_{max} \in (0,1)$.

The proposition is proved.

Remark. Note that in the case of $C = 1$ one obtains $J = D$. Therefore J has at most two distinct eigenvalues $\lambda_1 = \psi$ and $\lambda_2 = \psi + (G - C)\phi$. As a result, one concludes that $0 < \lambda_1, \lambda_2 < 1$ and the above equilibrium is locally stable.

Proposition 4. Consider an equilibrium with $1 \leq C < G$ cooperating groups. Let $\Pi_c^* < \Pi_d^*$. Assume also that each group produces a non-zero effort in the within-group game. Necessary conditions for the existence and local stability of the above equilibrium are:

(I) $R_2 > 1$ and $R \in (M_l(R_2, C), M_h(R_2, C))$, where $M_l(R_2, C)$ and $M_h(R_2, C)$ are defined according to formulas S12;

(II) for $C < G$: $Q_{C+1} \leq P_d^*$; or $Q_{C+1} > P_d^*$, and condition S9 holds, and $\varepsilon < \varepsilon^1_{max}$, where $0 \geq \varepsilon^1_{max} < 1$ is defined below;

(III)

$$\varepsilon f_c^*(\varepsilon) \leq \begin{cases} (1-\varepsilon)\frac{\Pi_c^*}{\Pi_d^* - \Pi_c}\frac{1}{f_c^*(\varepsilon)}, \text{ if } C = 1, G = 2; \\ (1-\varepsilon)\frac{\Pi_c^*}{\Pi_d^* - \Pi_c}, \text{ if } C = 1, G \geq 3; \\ (1-\varepsilon)\min\left\{\frac{\Pi_c^*}{\Pi_d^* - \Pi_c^*}, \frac{CA_c^*}{Q_C}\right\}, \text{ if } 2 \leq C \leq G-2 \\ (1-\varepsilon)\min\left\{\frac{\Pi_c^*}{\Pi_d^* - \Pi_c^*}\frac{1}{(G-1)f_c^*(\varepsilon)}, \frac{CA_c^*}{Q_C}\right\}, \text{ if } 2 \leq C = G-1 \end{cases}$$

where X_c^*, P_c^*, f_c^*, $A_c^* = \Pi^0 - cX_c^*$, and X_d^*, P_d^*, $A_d^* = \Pi^0 - cX_d^* + P_d^*$ are equilibrium characteristics of cooperating and defecting groups respectively.

Proof. Necessary conditions for the existence and local stability of the above equilibrium are: (1a) $P_d^* > 0$ (for $C < G$); (1b) $P_c^* > 0$; (1c) cooperating groups will not defect; (2) defecting groups will not start cooperating (for $C < G$); and (3) the state should be stable to small fluctuations in the power of groups.

The conditions (1a)-(1c) are equivalent to the condition (I) according to Proposition 1.

The condition (2) is equivalent to Inequality S7b. If $Q_{C+1} \leq P_d^*$, Inequality S7b is satisfied. Assume that $Q_{C+1} > P_d^*$ holds. Similarly to Proposition 2 one can show that $f_c^*(\varepsilon)$ is a **decreasing** function of ε on the interval $[0, 1]$. If condition S9 does not hold, $f_c^*(0) < \frac{1}{C}\frac{(Q_{C+1} - P_d^*)}{(Q_{C+1} - P_d^*) + (G-C)P_c^*}$ and, hence, for each $\varepsilon \in [0, 1]$ Inequality S7b is not satisfied. Otherwise, if condition S9 holds, Inequality S7b can be rewritten as $\varepsilon < \varepsilon^1_{max}$, where $\varepsilon^1_{max} = (f_c^*)^{-1}\left(\frac{1}{C}\frac{(Q_{C+1} - P_d^*)}{(Q_{C+1} - P_d^*) + (G-C)P_c^*}\right)$.

Finally, according to Proposition 3 the corresponding Jacobian matrix J have one, two or three distinct eigenvalues

$$\lambda_1 = \psi, \lambda_2 = \psi + \phi(G - C), \lambda_3 = \nu, \tag{S25}$$

depending on values of C and G (for more details see Proposition 3). Inequality $\lambda_1 \leq 1$ is equivalent to $\varepsilon f_c^*(\varepsilon) \leq (1-\varepsilon)\frac{\Pi_c^*}{\Pi_d^* - \Pi_c^*}$; inequality $\lambda_2 \leq 1$ is equivalent to $\varepsilon f_c^*(\varepsilon) \leq (1-\varepsilon)\frac{\Pi_c^*}{\Pi_d^* - \Pi_c^*}\frac{1}{Cf_c^*(\varepsilon)}$; and inequality $\lambda_3 \leq 1$ is equivalent to $\varepsilon f_c^*(\varepsilon) \leq (1-\varepsilon)\frac{CA_c^*}{Q_C}$. Since $\frac{1}{Cf_c^*(\varepsilon)} > 1$ we conclude that the condition (3) is equivalent to the condition (III).

References

[1] Goeree, J., Holt, C. & Palfrey, T. *Quantal Response Equilibrium: A Stochastic Theory of Games* (Princeton University Press, Princeton, NJ, 2016).

[2] Gavrilets, S. & Shrestha, M. Evolving institutions for collective action by selective imitation and self-interested design. *SocArXiv* (2019).

[3] Nöldeke, G. & Peña, J. Group size and collective action in a binary contribution game. *Journal of Mathematical Economics* **88**, 42–51 (2020).

[4] Houle, C., Ruck, D., Bentley, R. & Gavrilets, S. Horizontal inequality and instability. *Unpublished* (2021).

2 Part 2. Additional simulation results

2.1 Groups with identical sizes

2.1.1 Equilibria

Figures S5-S9 show the effects of the incumbency parameter ε on the number of cooperating groups C (a,d), the number of cooperating individuals per group X (b,e) and group power f (c,f) for different values of the benefit parameter b_2 and the cost parameter Z_0. Groups of the fixed size $n = 10$ are considered. First row of graphs: equilibria with just one type of cooperating groups. Defecting groups are shown in blue symbols. Second row of graphs: equilibria with dominant (violet symbols) and subordinate (golden symbols) cooperating groups. Curves show the average values of corresponding characteristics. The equilibria illustrated in the top and the bottom graphs are simultaneously stable. Baseline parameters: $b_1 = 20$, $\alpha = 1$, $c = 1$, $\pi^0 = 1$, $X_0 = 5$, $G = 8$, $\mu_1 = \mu_2 = 0.25$. Initially, each individual and group cooperate randomly with probability 0.5 while group powers were chosen from a broken stick distribution. The results shown are based on 200 runs with 4,000 time steps for each parameter combination. The outcomes for each run are averages of the last 1000 time steps.

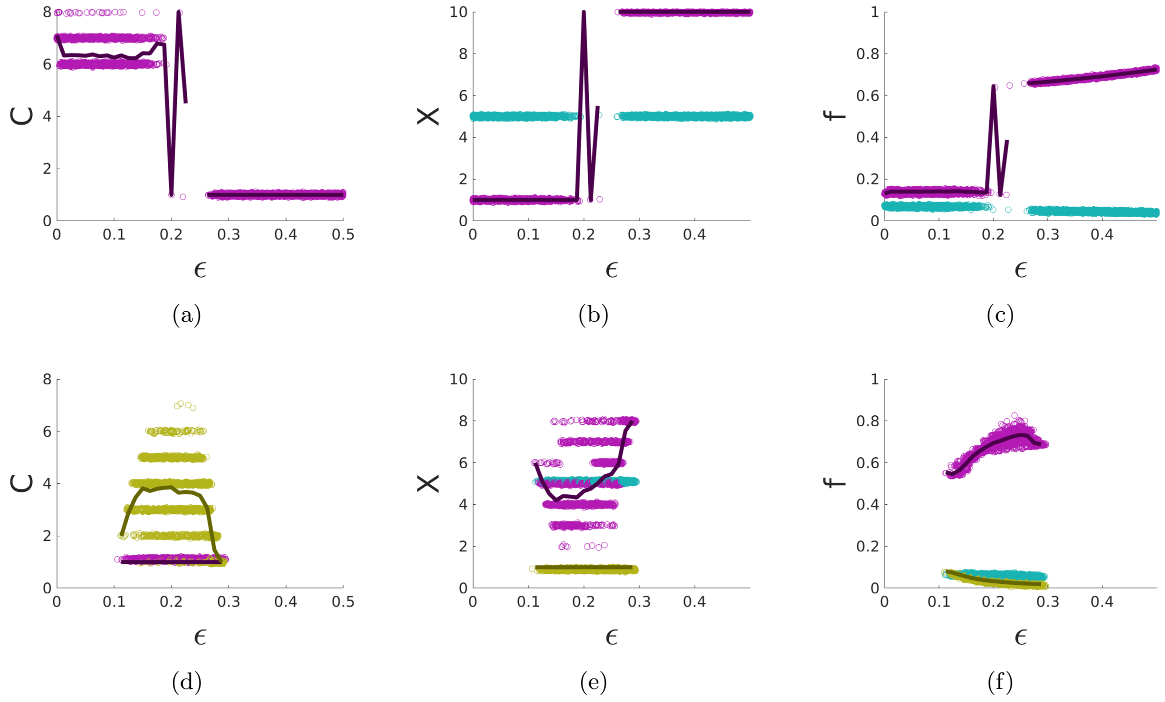

Figure S5: $b_2 = 20$, $Z0 = 50$

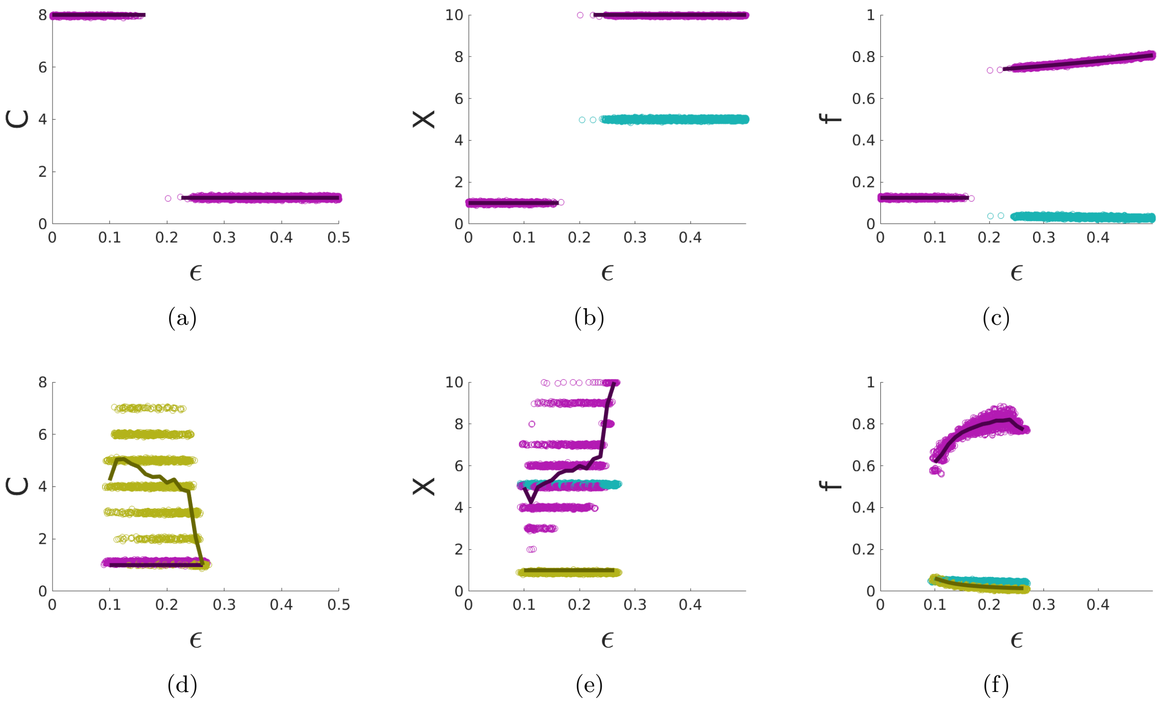

Figure S6: $b_2 = 30,\ Z0 = 50$

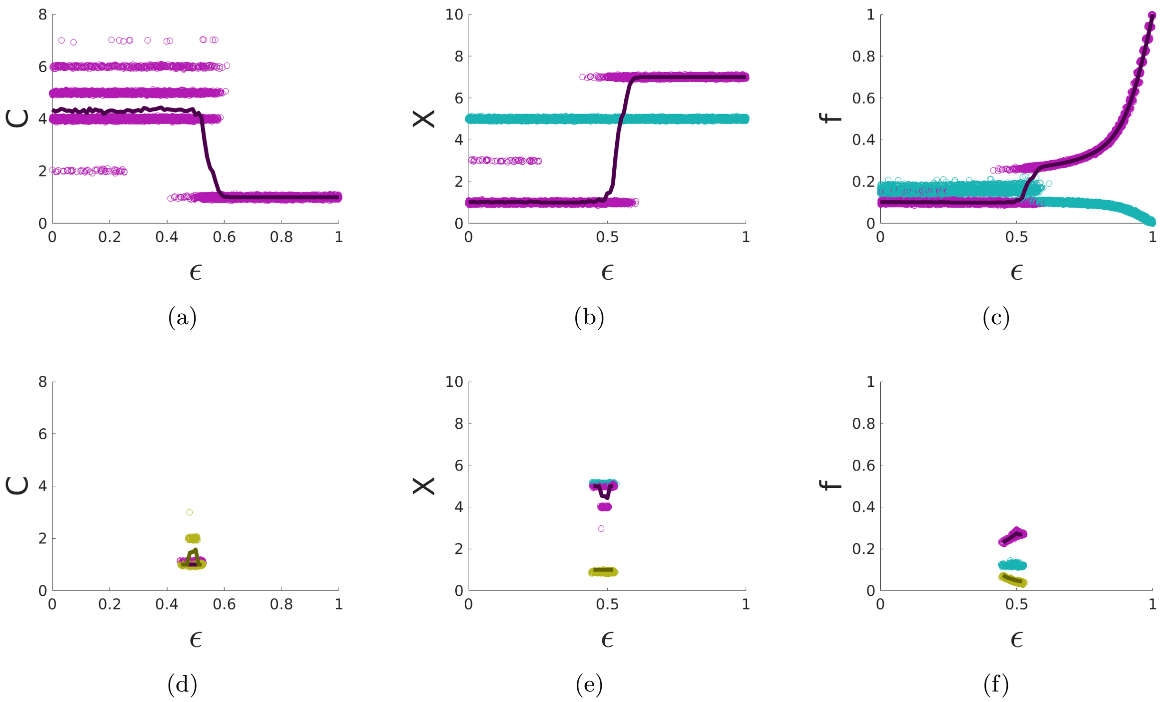

Figure S7: $b_2 = 10,\ Z0 = 300$

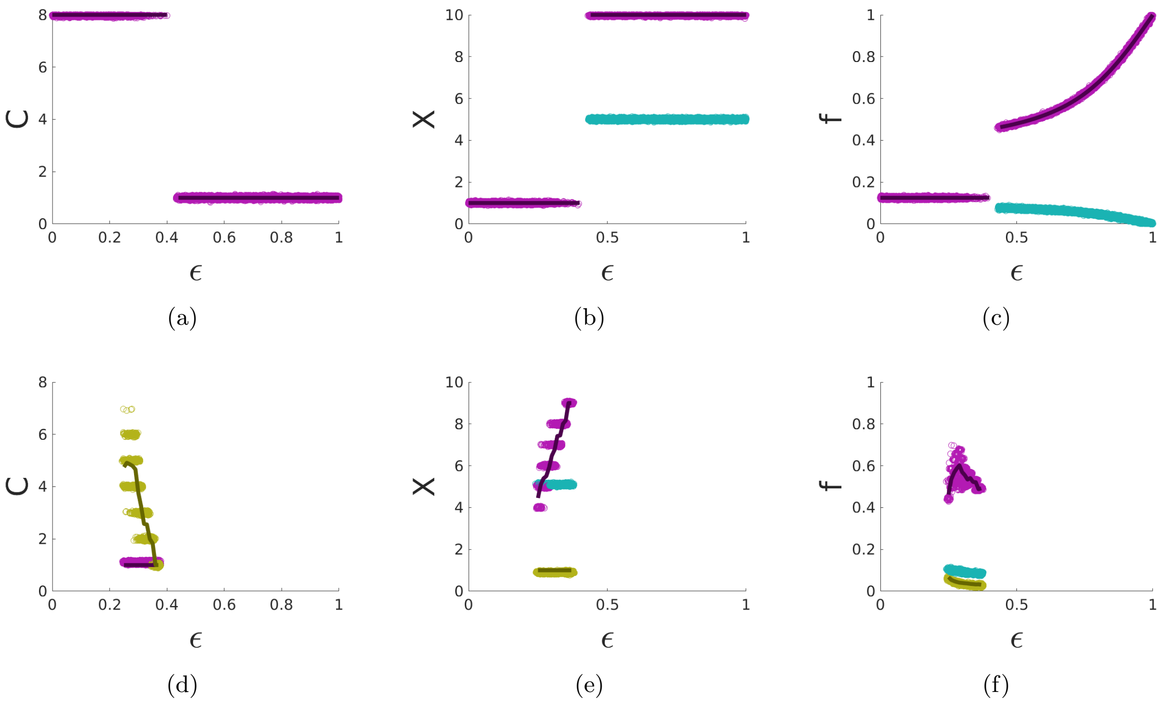

Figure S8: $b_2 = 20,\ Z0 = 300$

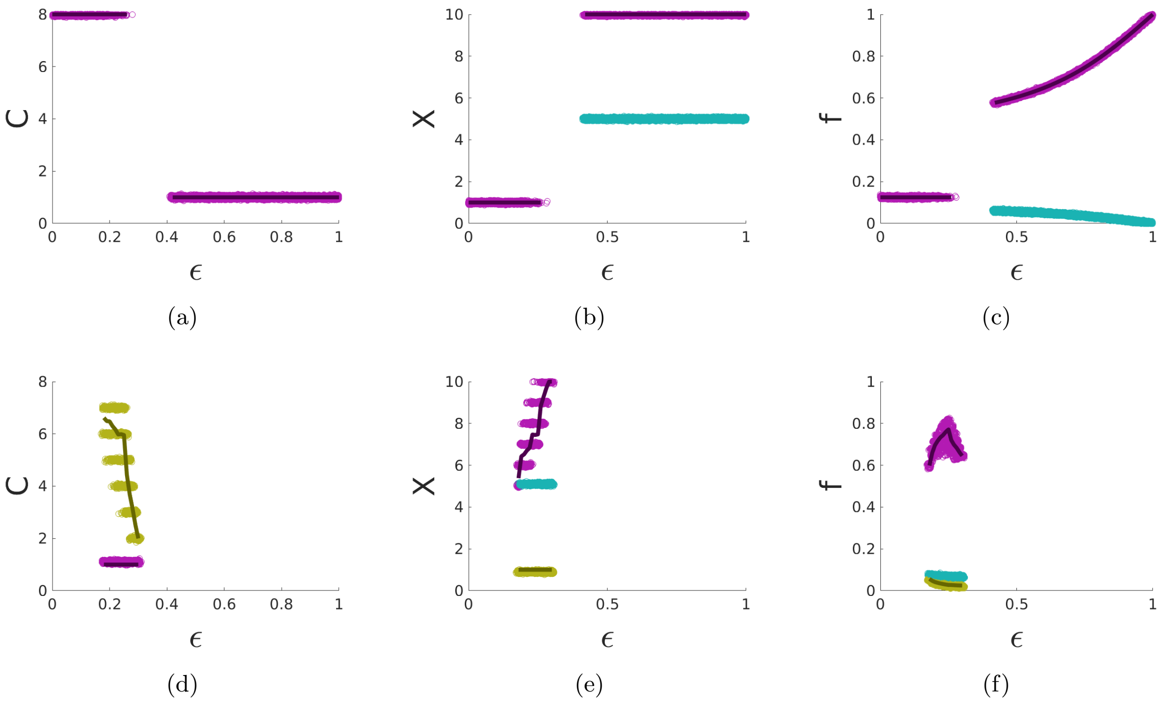

Figure S9: $b_2 = 30,\ Z0 = 300$

Figures S10-S12 show the effects of the incumbency parameter ε on the number of cooperating groups C (a,d), the number of cooperating individuals per group X (b,e) and group power f (c,f) for groups of the fixed size n. First row of graphs: equilibria with just one type of cooperating groups. Defecting groups are shown in blue symbols. Second row of graphs: equilibria with dominant (violet symbols) and subordinate (golden symbols) cooperating groups. Curves show the average values of corresponding characteristics. The equilibria illustrated in the top and the bottom graphs are simultaneously stable. Baseline parameters: $B_1 = 200$, $B_2 = 800$, $\alpha = 1$, $c = 1$, $\pi^0 = 1$, $X_0 = 5$, $Z_0 = 50$, $G = 8$, $\mu_1 = \mu_2 = 0.25$. Initially, each individual and group cooperate randomly with probability 0.5 while group powers were chosen from a broken stick distribution. The results shown are based on 100 runs with 4,000 time steps for each parameter combination. The outcomes for each run are averages of the last 1000 time steps.

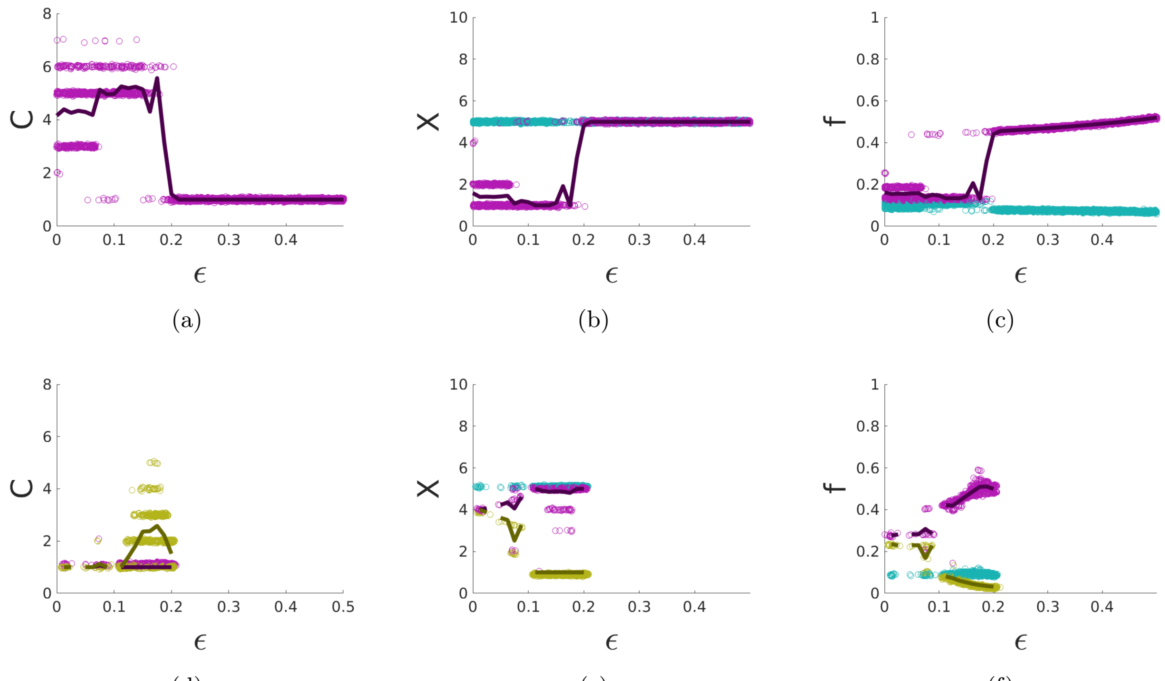

(a) (b) (c)

(d) (e) (f)

Figure S10: $n = 5$

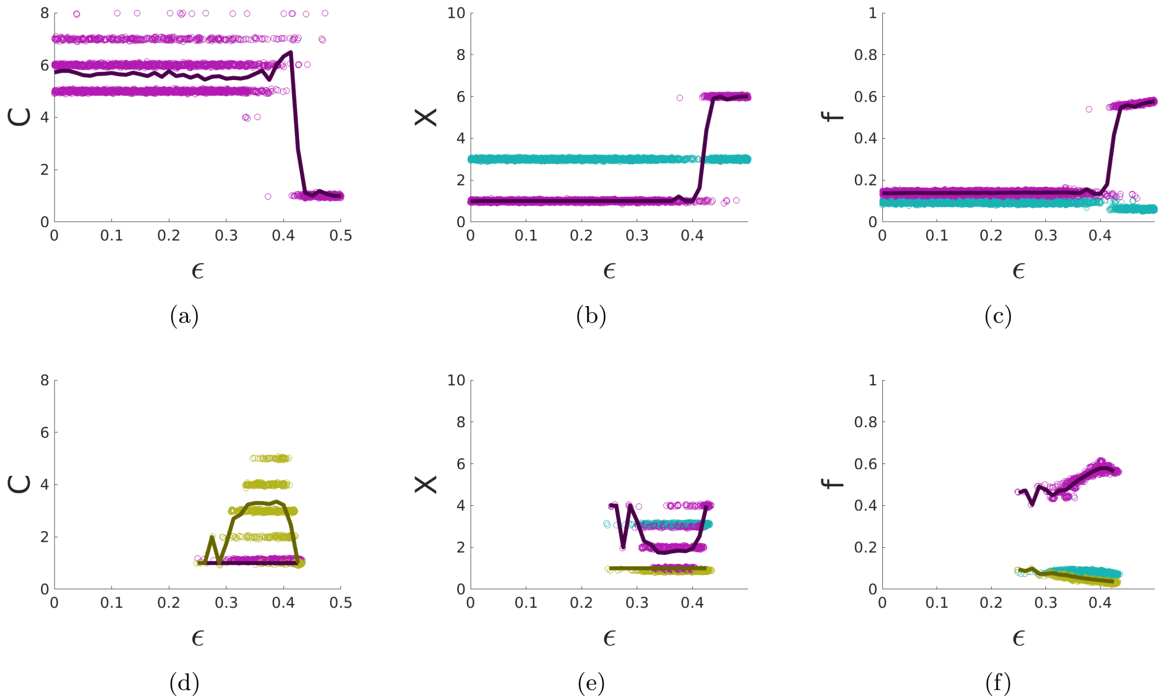

Figure S11: $n = 15$

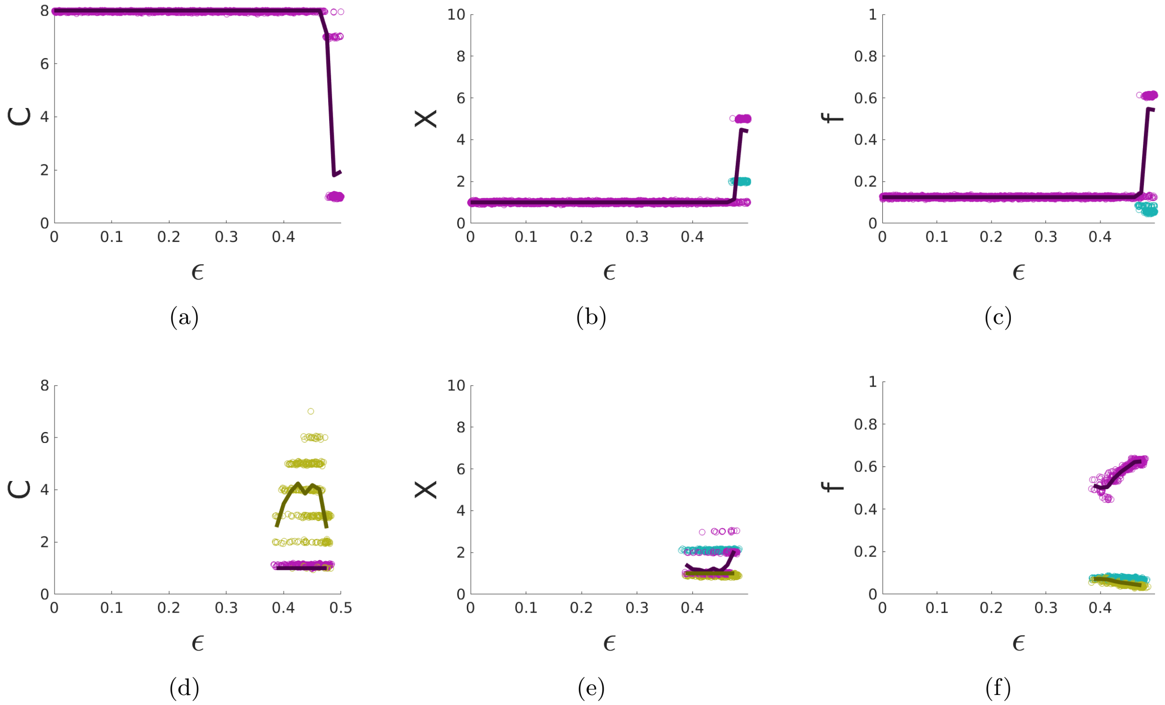

Figure S12: $n = 20$

Figure S13 shows effects of the incumbency parameter ε on the number of cooperating groups C and the normalized resource produced Q/N (where $N = nG$) for different values of the benefit parameter b_2 and the cost parameter Z_0. Groups of the fixed size $n = 10$ are considered. Each point corresponds to an outcome of a particular run. Equilibria with just one type of cooperating groups are colored in violet. Equilibria with dominant and subordinate cooperating groups are colored in gold. Baseline parameters: $b_1 = 20$, $\alpha = 1$, $c = 1$, $\pi^0 = 1$, $X_0 = 5$, $G = 8$, $\mu_1 = \mu_2 = 0.25$. Initially, each individual and group cooperate randomly with probability 0.5 while group powers were chosen from a broken stick distribution. The results shown are based on 200 runs with 4,000 time steps for each parameter combination. The outcomes for each run are averages of the last 1000 time steps.

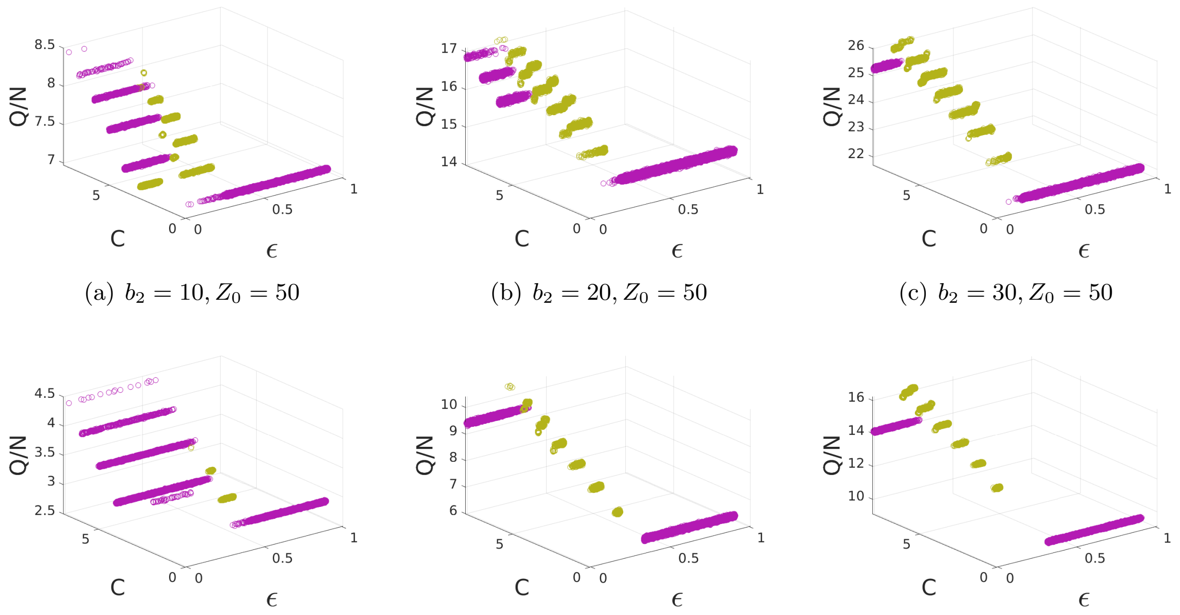

(a) $b_2 = 10, Z_0 = 50$ (b) $b_2 = 20, Z_0 = 50$ (c) $b_2 = 30, Z_0 = 50$

(d) $b_2 = 10, Z_0 = 300$ (e) $b_2 = 20, Z_0 = 300$ (f) $b_2 = 30, Z_0 = 300$

Figure S13: Effects of the incumbency parameter ε on the number of cooperating groups C and the normalized resource produced Q/N.

2.1.2 Non-equilibrium dynamics

Figures S14 and S15 show effects of incumbency parameter ε and the benefit parameter b_2 on the frequency of non-equilibrium dynamics. Groups of the same size $n = 10$ are considered. Baseline parameters: $b_1 = 10$, $\alpha = 1$, $c = 1$, $\pi^0 = 1$, $X_0 = 5$, $Z_0 = 50$, $\mu_1 = \mu_2 = 0.25$. Initially, each individual and group cooperate randomly with probability 0.5 while group powers were chosen from a broken stick distribution.

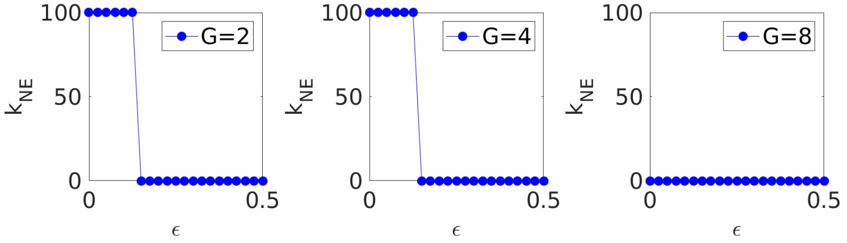

(a) $b_2 = 32$

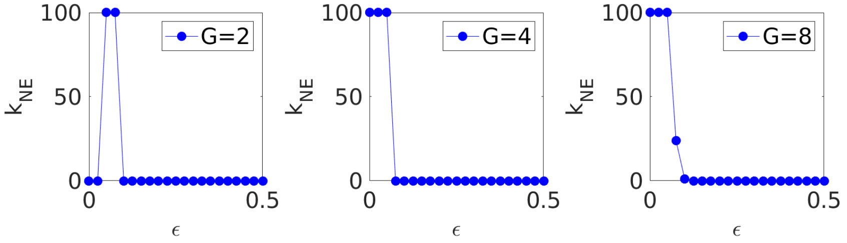

(b) $b_2 = 64$

Figure S14: Effects of incumbency parameter ε, the benefit parameter b_2 and the number of groups G on the frequency of runs with non-equilibrium dynamics. The results shown are based on 100 runs with 4,000 time steps for each parameter combination. The outcomes for each run are averages of the last 1000 time steps.

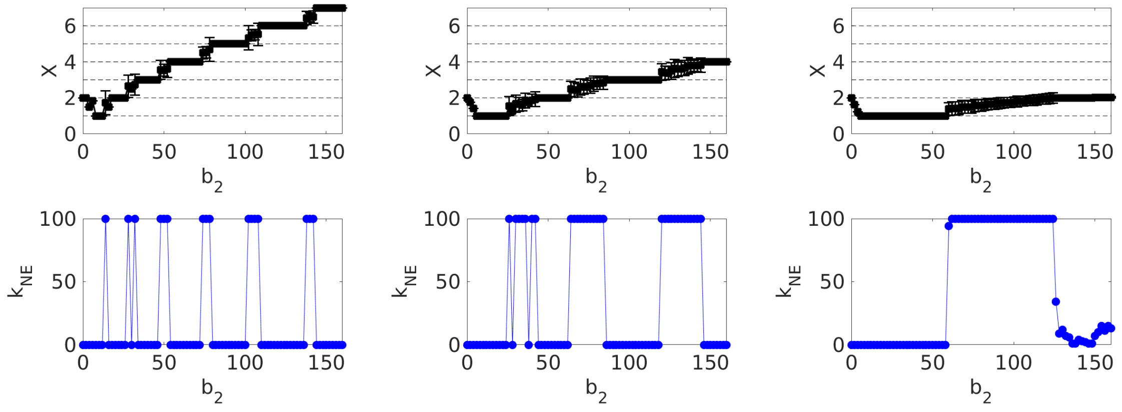

(a) $G = 2$ (b) $G = 4$ (c) $G = 8$

Figure S15: Effects of the benefit parameter b_2 and the number of groups G on the frequency of runs with non-equilibrium dynamics k_{NE}. Incumbency parameter is equal to zero.The results shown are based on 100 runs with 8,000 time steps for each parameter combination. The outcomes for each run are averages of the last 2000 time steps.

2.1.3 Effects of parameters

Here we will first discuss effect of parameters Z_0, λ and G on the model dynamics. We will then provide more results of numerical simulations underlying our conclusions about effects of various parameters.

The cost parameter Z_0*.* Effects of Z_0 depend on other parameters. If the benefit of between-group cooperation b_2 is low (relative to the benefit of within-group cooperation b_1), a group has little motivation to cooperate. As a result, increasing cost Z_0 decreases the number of cooperating groups C (see Fig. S18a). If b_2 is moderate or high and ε is relatively low an increase in Z_0 first leads to an increase in the coalition size, which then decreases as Z_0 becomes sufficiently high (Fig. S18b,c). An explanation is intuitive: increasing Z_0 encourages groups to cooperate in order to obtain high benefits associated with large b_2. However, extremely high costs Z_0 overcome benefits of cooperation associated with high values of b_2. With large ε all groups except one do not cooperate regardless of Z_0 (Fig. S18).

Precision parameter λ*.* All of the results presented so far (here and in the main text) assumed infinite precision ($\lambda = \infty$). Decreasing precision parameter λ decreases the number of coexisting equilibria and increases the frequency of non-equilibrium dynamics (Figs. S19 - S22). Nevertheless, if λ is not too small, all the average trends are similar to those obtained for infinite precision (Figs. S19 - S22). If $\lambda \approx 0$, then on average half of the groups and half of the individuals in each group cooperate, since all agents choose their strategies at random.

The number of groups G*.* All the effects of parameters discussed so far (here and in the main text) are more or less the same regardless of the number of groups G. However, increasing G increases the Gini index of inequality I among cooperating groups and the standard deviation σ of their efforts.

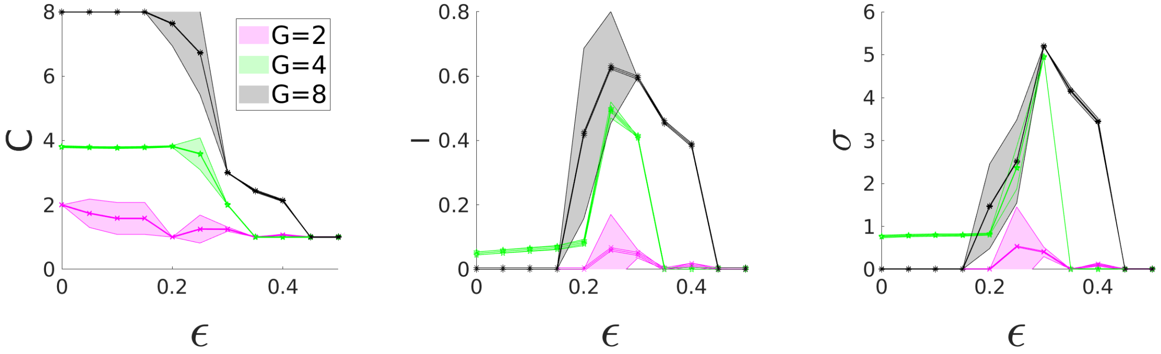

(a) (b) (c)

Figure S16: Effects of the incumbency parameter ε on the number of cooperating groups C, the Gini index of inequality among them I, and standard deviation of efforts σ among them for different number of groups G. Baseline parameters: $b_1 = 20$, $b_2 = 30$, $n = 10$, $\alpha = 1$, $c = 1$, $\pi^0 = 1$, $X_0 = 5, Z_0 = 300$, $\mu_1 = \mu_2 = 0.25$. Initially, each individual and group cooperate randomly with probability 0.5, and group powers were chosen from a broken stick distribution. The figures show the averages and confidence intervals based on 100 runs each of 4000 time steps for each parameter combination. Results in each run are averages based on the last 1000 of time steps.

Figures S17-S19 show effects of the benefit parameter b_1 on the number of cooperating groups C, the Gini index of inequality among them I, and standard deviation of efforts σ among them for different values of the incumbency parameter ε, the benefit parameter b_2, and the number of groups G. Baseline parameters: $n = 10$, $\alpha = 1$, $c = 1$, $\pi^0 = 1$, $X_0 = 5$, $Z_0 = 50$, $\mu_1 = \mu_2 = 0.25$. Initially, each individual and group cooperate randomly with probability 0.5, and group powers were chosen from a broken stick distribution. The figures show the averages and confidence intervals based on 100 runs each of 4000 time steps for each parameter combination. Results in each run are averages based on the last 1000 of time steps.

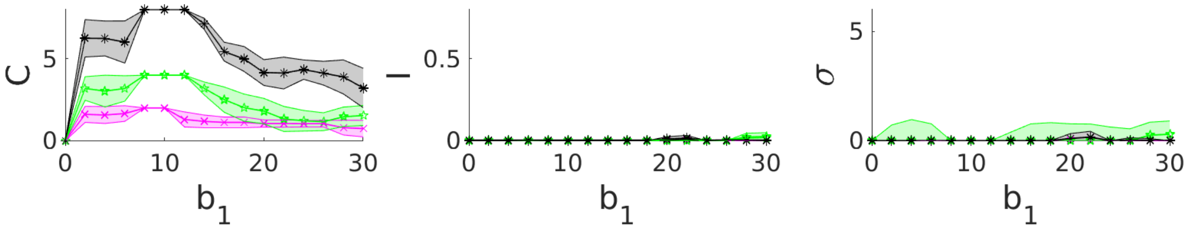

(a) $\varepsilon = 0.1$

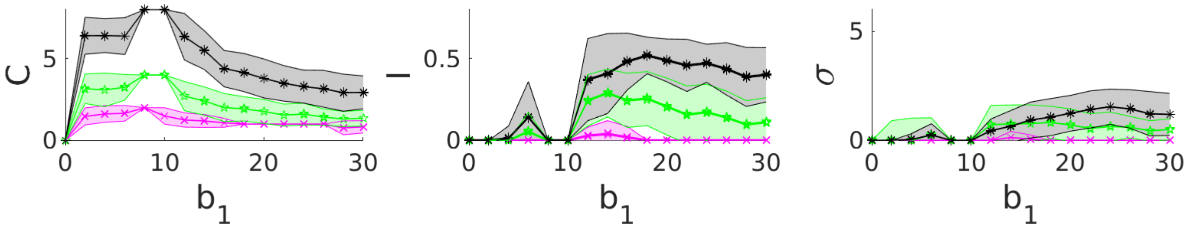

(b) $\varepsilon = 0.3$

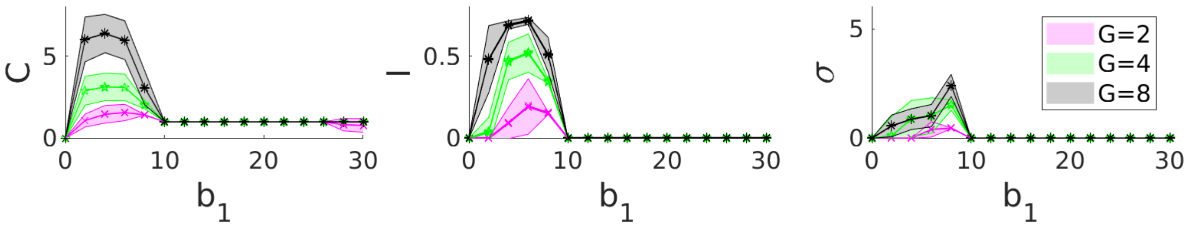

(c) $\varepsilon = 0.5$

Figure S17: $b_2 = 10$

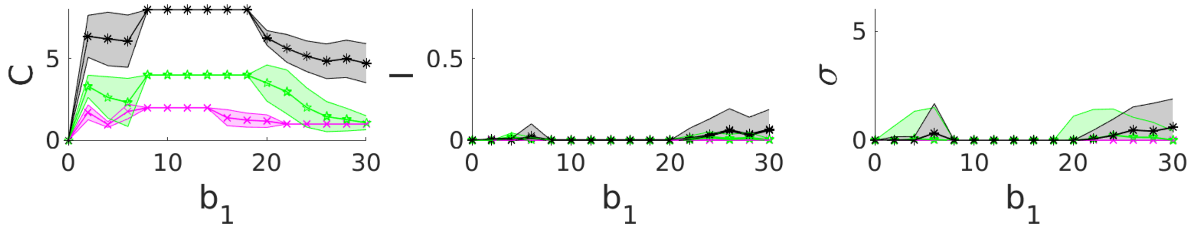

(a) $\varepsilon = 0.1$

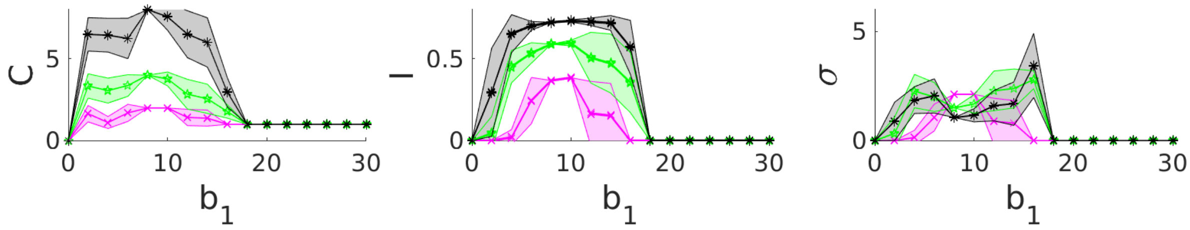

(b) $\varepsilon = 0.3$

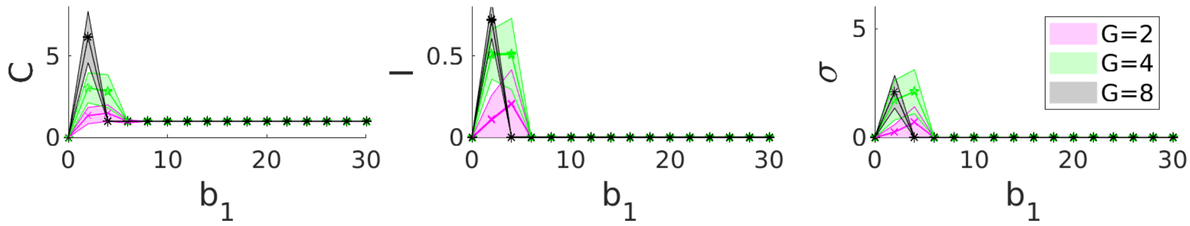

(c) $\varepsilon = 0.5$

Figure S18: $b_2 = 20$

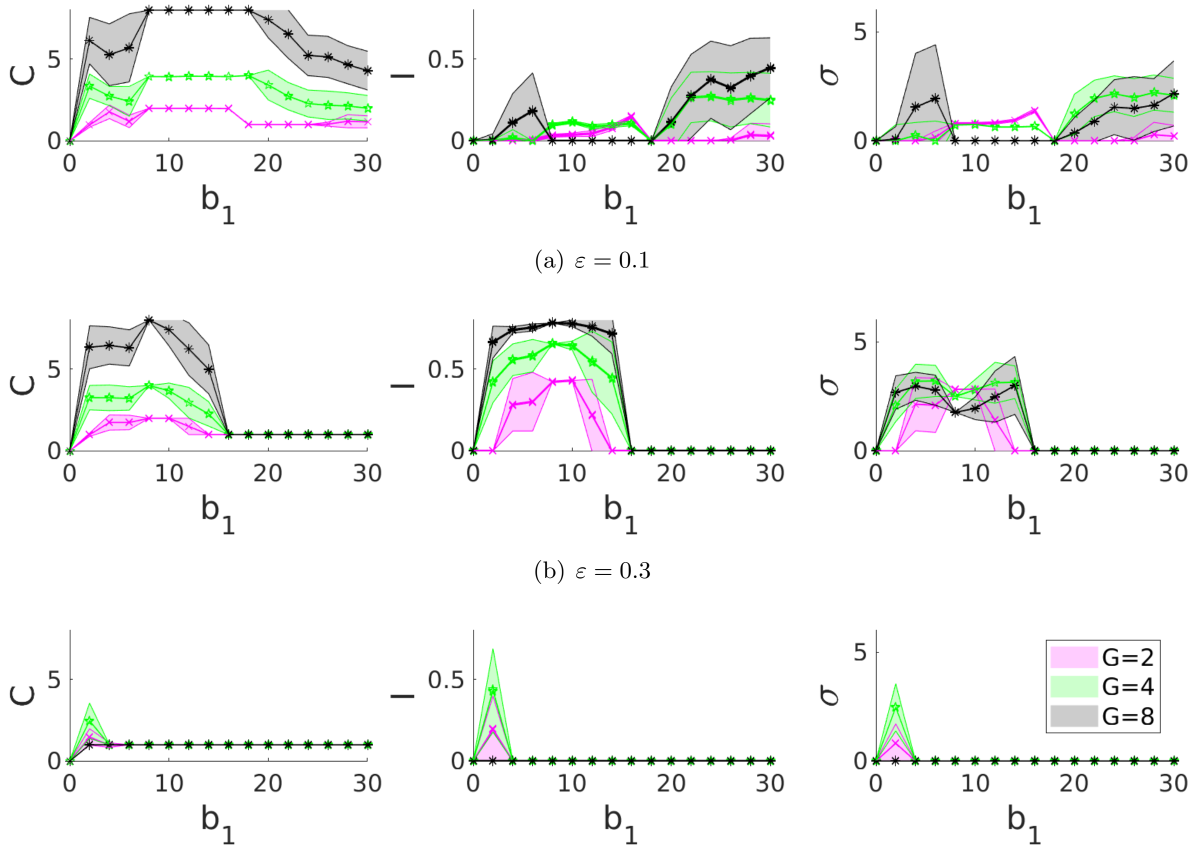

(a) $\varepsilon = 0.1$

(b) $\varepsilon = 0.3$

(c) $\varepsilon = 0.5$

Figure S19: $b_2 = 30$

Figures S20-S21 show effects of the benefit parameter b_2 on the number of cooperating groups C, the Gini index of inequality among them I, and standard deviation of efforts σ among them for different values of the incumbency parameter ε, the cost parameter Z_0, and the number of groups G. Baseline parameters: $b_1 = 20$, $n = 10$, $\alpha = 1$, $c = 1$, $\pi^0 = 1$, $X_0 = 5$, $\mu_1 = \mu_2 = 0.25$. Initially, each individual and group cooperate randomly with probability 0.5, and group powers were chosen from a broken stick distribution. The figures show the averages and confidence intervals based on 100 runs each of 4000 time steps for each parameter combination. Results in each run are averages based on the last 1000 of time steps.

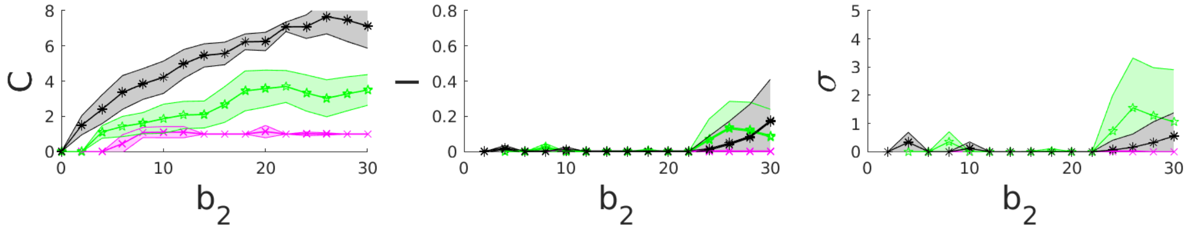

(a) $\varepsilon = 0.1$

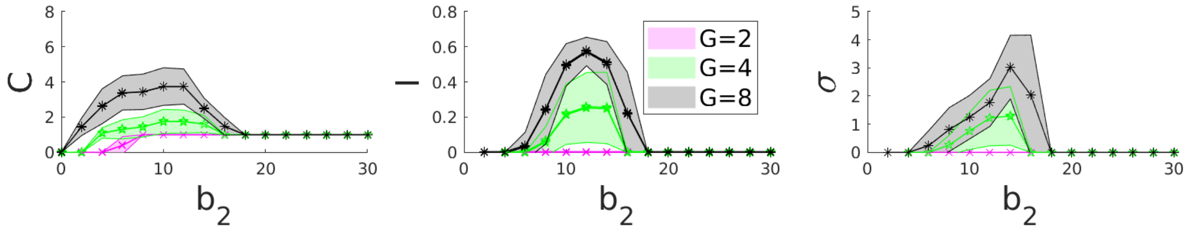

(b) $\varepsilon = 0.3$

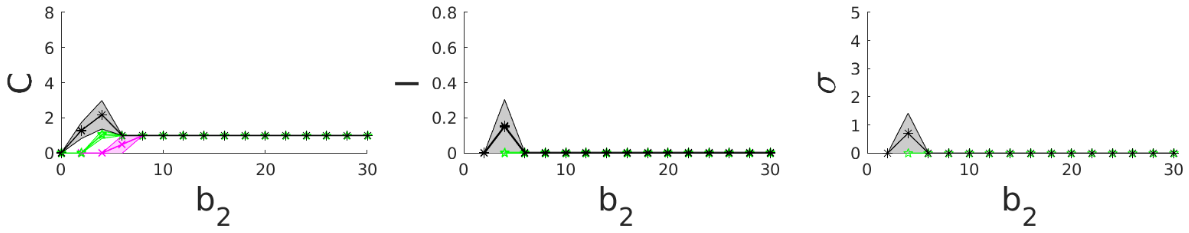

(c) $\varepsilon = 0.5$

Figure S20: $Z_0 = 50$

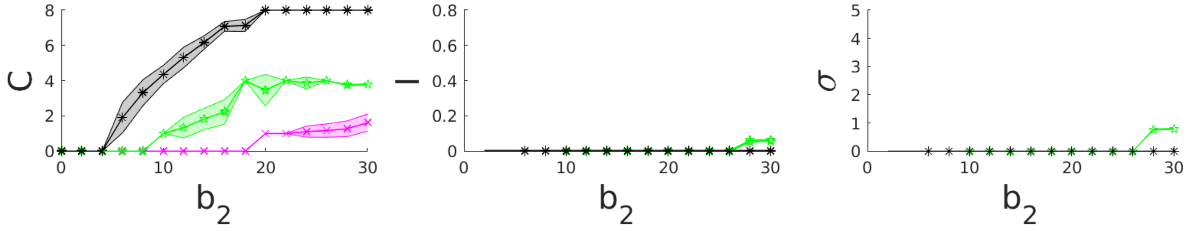

(a) $\varepsilon = 0.1$

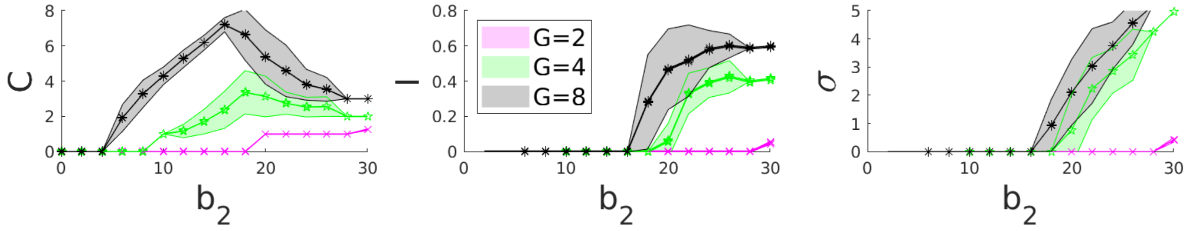

(b) $\varepsilon = 0.3$

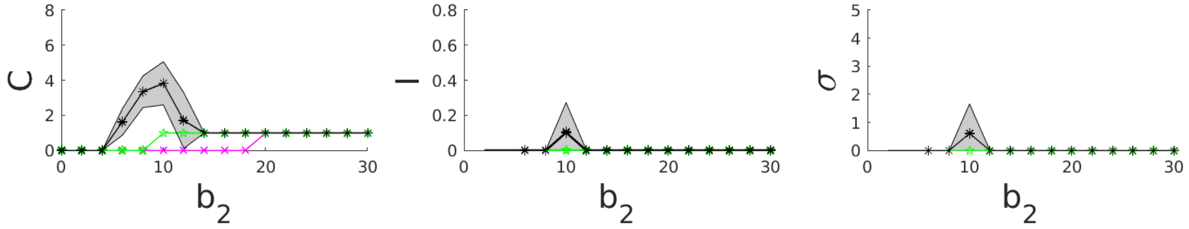

(c) $\varepsilon = 0.5$

Figure S21: $Z_0 = 300$

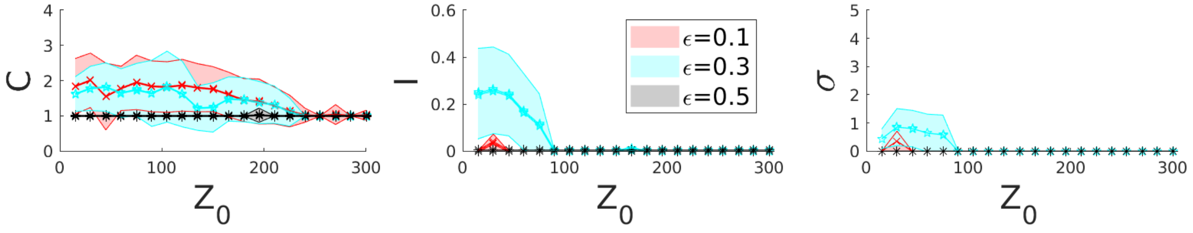

(a) $b_2 = 10$

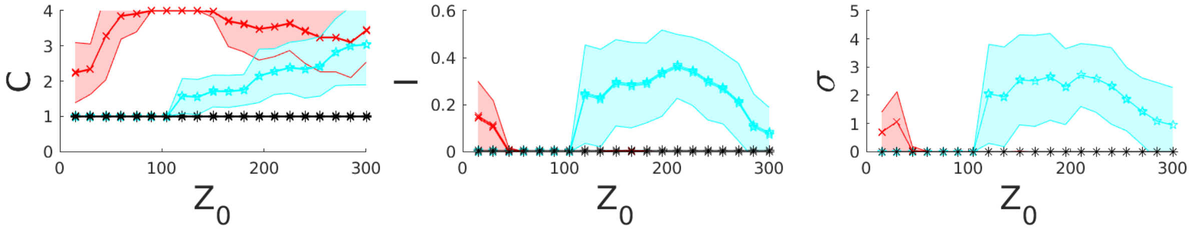

(b) $b_2 = 20$

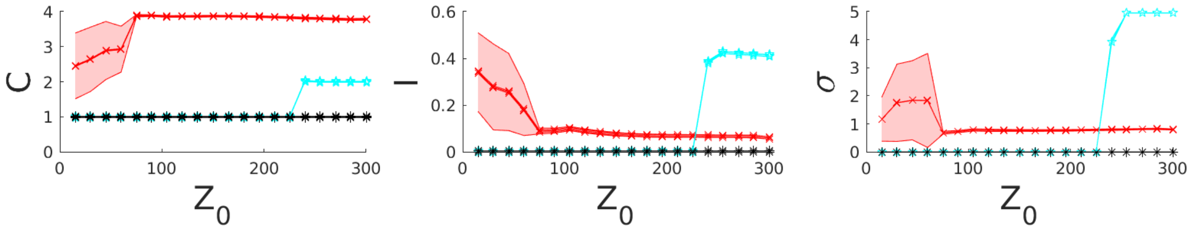

(c) $b_2 = 30$

Figure S22: Effects of the cost parameter Z_0 on the number of cooperating groups C, the Gini index of inequality I, and standard deviation of efforts σ among them for different values of incumbency parameter ε and the benefit parameter b_2. Baseline parameters: $b_1 = 20$, $n = 10$, $\alpha = 1$, $c = 1$, $\pi^0 = 1$, $X_0 = 5$, $G = 4$, $\mu_1 = \mu_2 = 0.25$. Initially, each individual and group cooperate randomly with probability 0.5, and group powers were chosen from a broken stick distribution. The figures show the averages and confidence intervals based on 100 runs each of 4000 time steps for each parameter combination. Results in each run are averages based on the last 1000 of time steps.

Figures S23-S26 show effects of different model's parameters on the number of cooperating groups C, the Gini index of inequality among them I, and standard deviation of efforts σ among them for different finite values of the precision parameter λ. Groups of the same size n are considered.

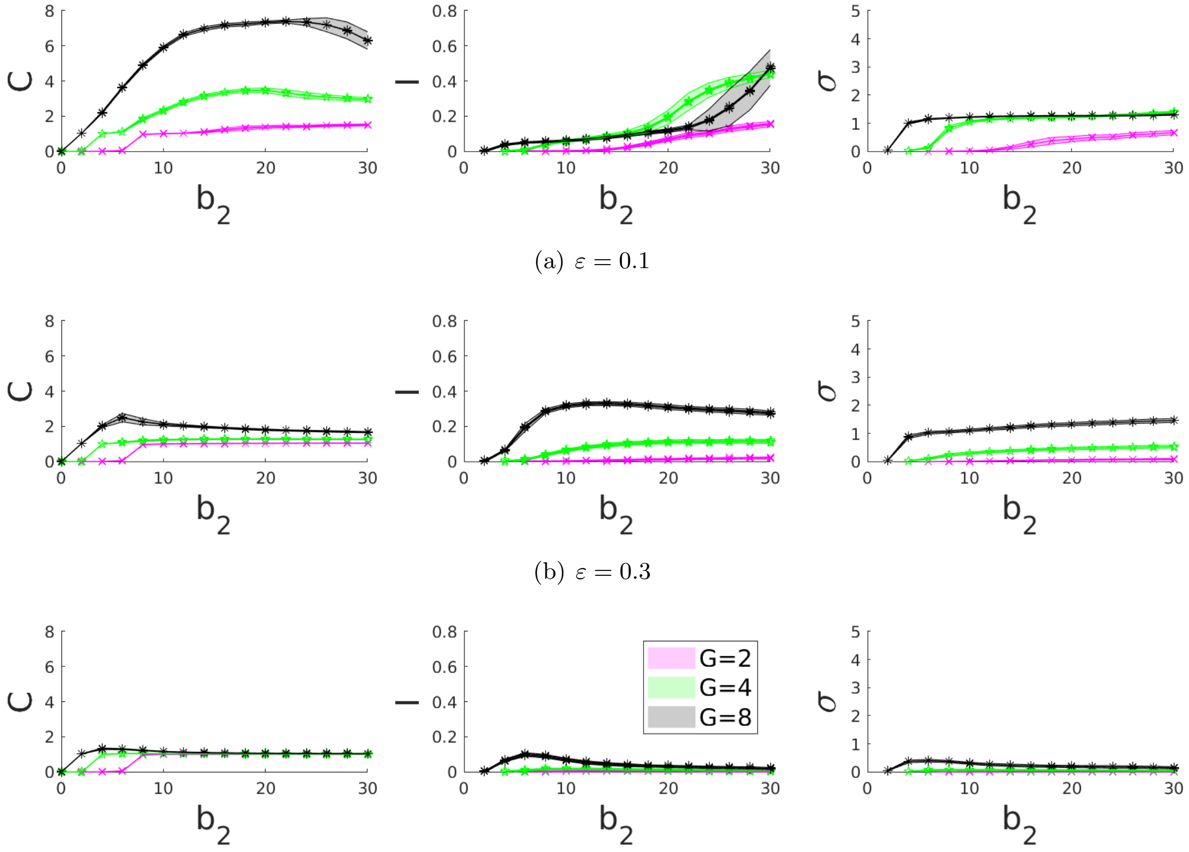

(a) $\varepsilon = 0.1$

(b) $\varepsilon = 0.3$

(c) $\varepsilon = 0.5$

Figure S23: Effects of the benefit parameter b_2 on the number of cooperating groups C, the Gini index of inequality I, and standard deviation of efforts σ among them for different values of incumbency parameter ε and the number of groups G. Precision parameter λ is equal to 1. Baseline parameters: $b_1 = 20$, $n = 10$, $\alpha = 1$, $c = 1$, $\pi^0 = 1$, $X_0 = 5$, $Z_0 = 50$, $\mu_1 = \mu_2 = 0.25$. Initially, each individual and group cooperate randomly with probability 0.5, and group powers were chosen from a broken stick distribution. The figures show the averages and confidence intervals based on 100 runs each of 4000 time steps for each parameter combination. Results in each run are averages based on the last 1000 of time steps.

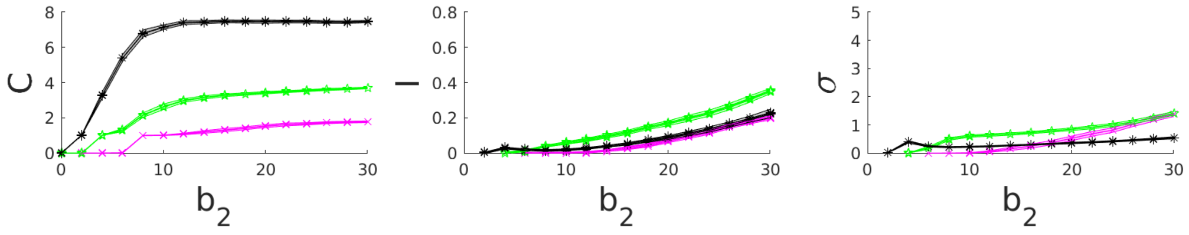

(a) $\varepsilon = 0.1$

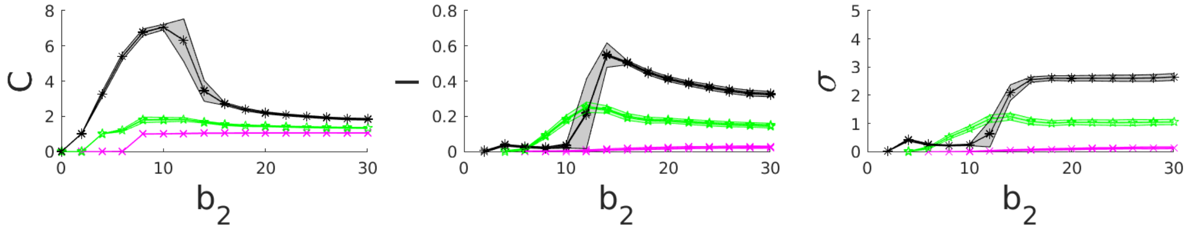

(b) $\varepsilon = 0.3$

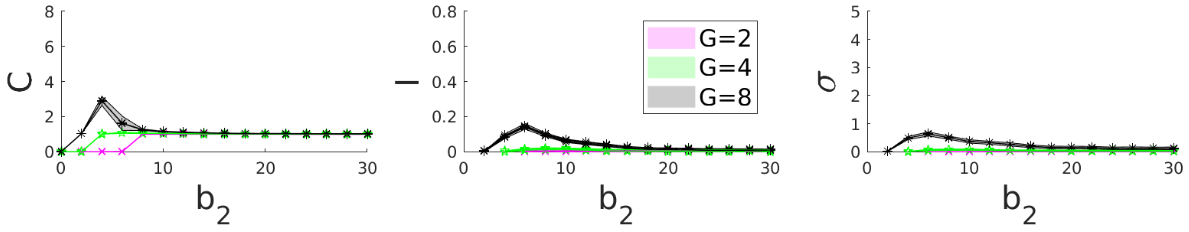

(c) $\varepsilon = 0.5$

Figure S24: Effects of the benefit parameter b_2 on the number of cooperating groups C, the Gini index of inequality I, and standard deviation of efforts σ among them for different values of incumbency parameter ε and the number of groups G. Precision parameter λ is equal to 5. Baseline parameters: $b_1 = 20$, $n = 10$, $\alpha = 1$, $c = 1$, $\pi^0 = 1$, $X_0 = 5$, $Z_0 = 50$, $\mu_1 = \mu_2 = 0.25$. Initially, each individual and group cooperate randomly with probability 0.5, and group powers were chosen from a broken stick distribution. The figures show the averages and confidence intervals based on 100 runs each of 4000 time steps for each parameter combination. Results in each run are averages based on the last 1000 of time steps.

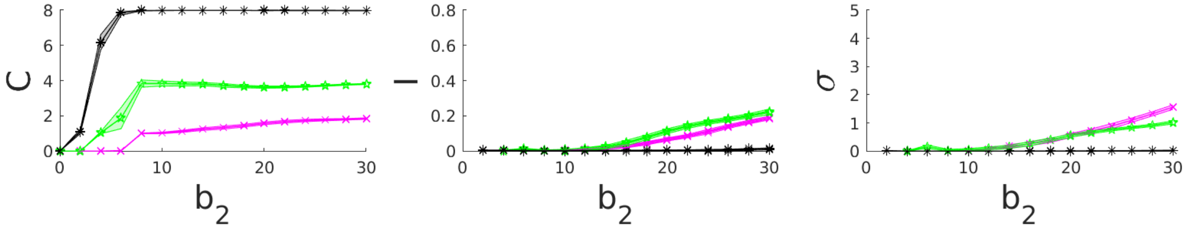

(a) $\varepsilon = 0.1$

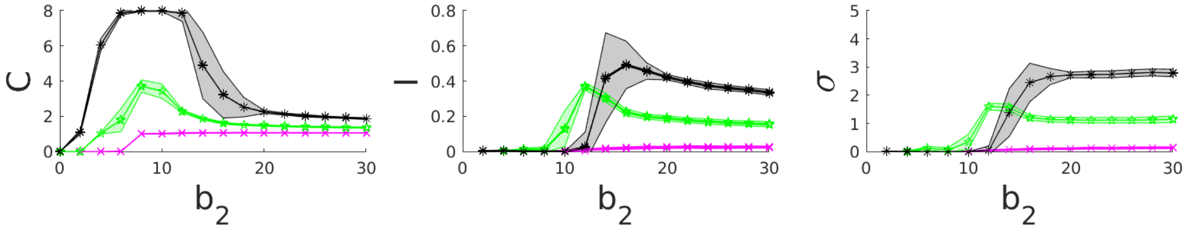

(b) $\varepsilon = 0.3$

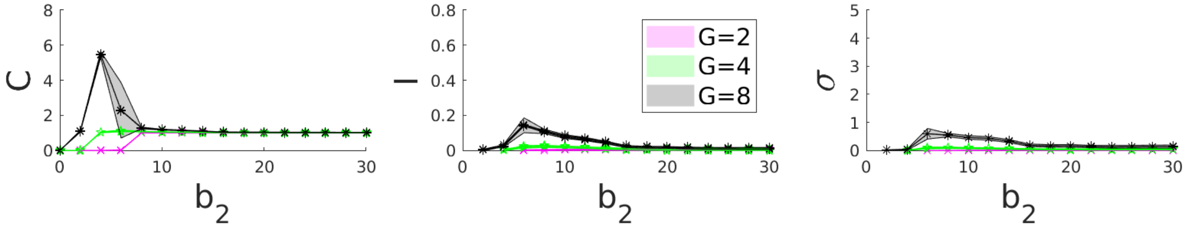

(c) $\varepsilon = 0.5$

Figure S25: Effects of the benefit parameter b_2 on the number of cooperating groups C, the Gini index of inequality I, and standard deviation of efforts σ among them for different values of incumbency parameter ε and the number of groups G. Precision parameter λ is equal to 10. Baseline parameters: $b_1 = 20$, $n = 10$, $\alpha = 1$, $c = 1$, $\pi^0 = 1$, $X_0 = 5$, $Z_0 = 50$, $\mu_1 = \mu_2 = 0.25$. Initially, each individual and group cooperate randomly with probability 0.5, and group powers were chosen from a broken stick distribution. The figures show the averages and confidence intervals based on 100 runs each of 4000 time steps for each parameter combination. Results in each run are averages based on the last 1000 of time steps.

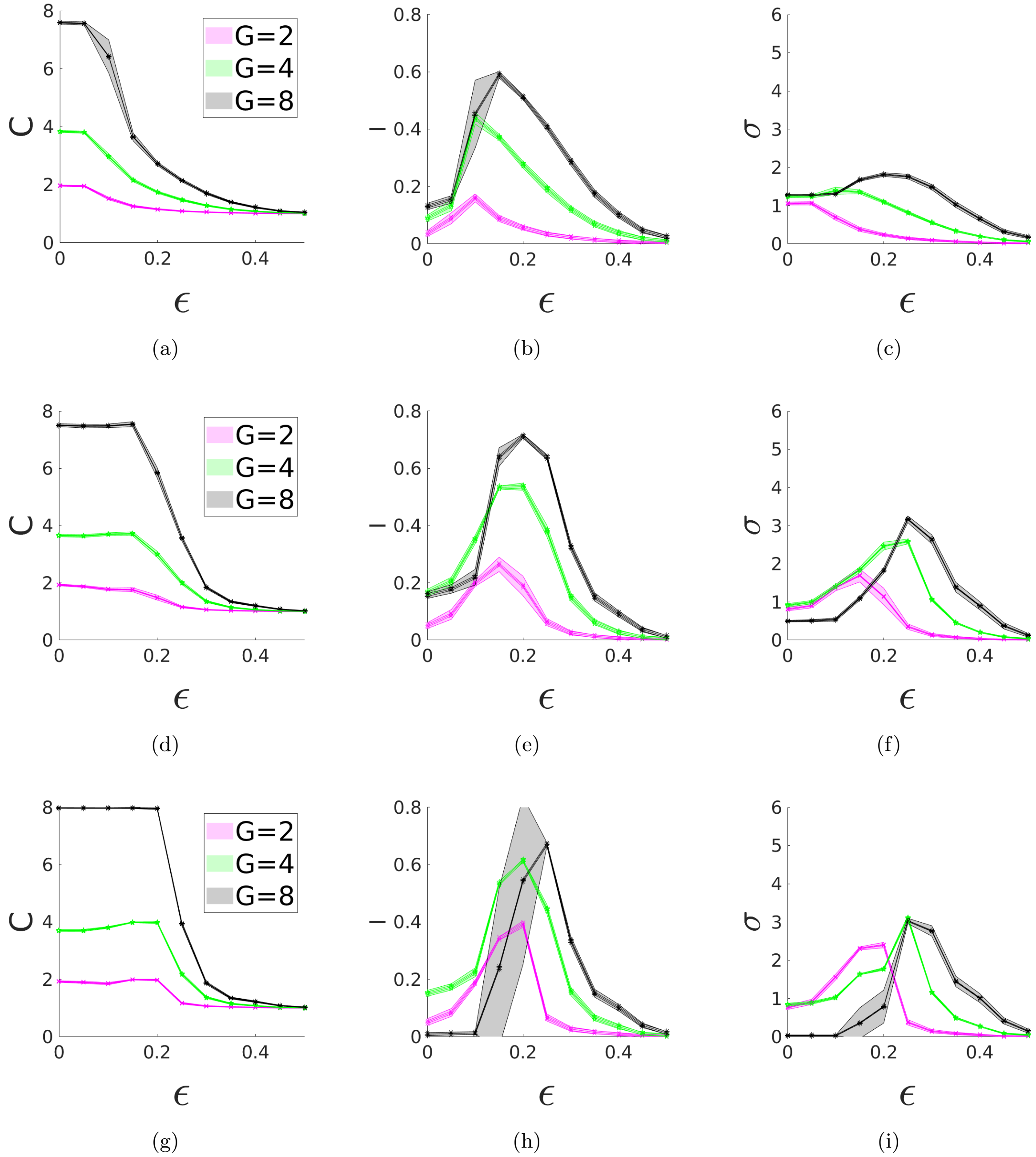

Figure S26: Effects of the incumbency parameter ε on the number of cooperating groups C, the Gini index of inequality I, and standard deviation of efforts σ among them for different number of groups G and different values of precision parameter. Precision $\lambda = 1, 5, 10$ for top, middle and bottom figures respectively. Baseline parameters: $b_1 = 20$, $b_2 = 30$, $n = 10$, $\alpha = 1$, $c = 1$, $\pi^0 = 1$, $X_0 = 5, Z_0 = 300$, $\mu_1 = \mu_2 = 0.25$. Initially, each individual and group cooperate randomly with probability 0.5, and group powers were chosen from a broken stick distribution. The figures show the averages and confidence intervals based on 100 runs each of 4000 time steps for each parameter combination. Results in each run are averages based on the last 1000 of time steps.

2.2 Groups with different sizes

2.2.1 Effects of parameters, rivalrous good

Figures S27-S29 show effects of the benefit parameter b_2 on the average number of contributing individuals X, the average cooperation status θ and average power f of each group for different values of the incumbency parameter ε, the benefit parameter B_1 and the costs parameter Z_0. A society of 4 groups containing 5, 10, 15 and 20 individuals respectively is considered. Baseline parameters: $\alpha = 1$, $c = 1$, $\pi^0 = 1$, $X_0 = 5$, $\mu_1 = \mu_2 = 0.25$. Initially, each individual and group cooperate randomly with probability 0.5, and equal power of groups. The figures show the averages based on 200 runs each of 4000 time steps for each parameter combination. Results in each run are averages based on the last 1000 time steps.

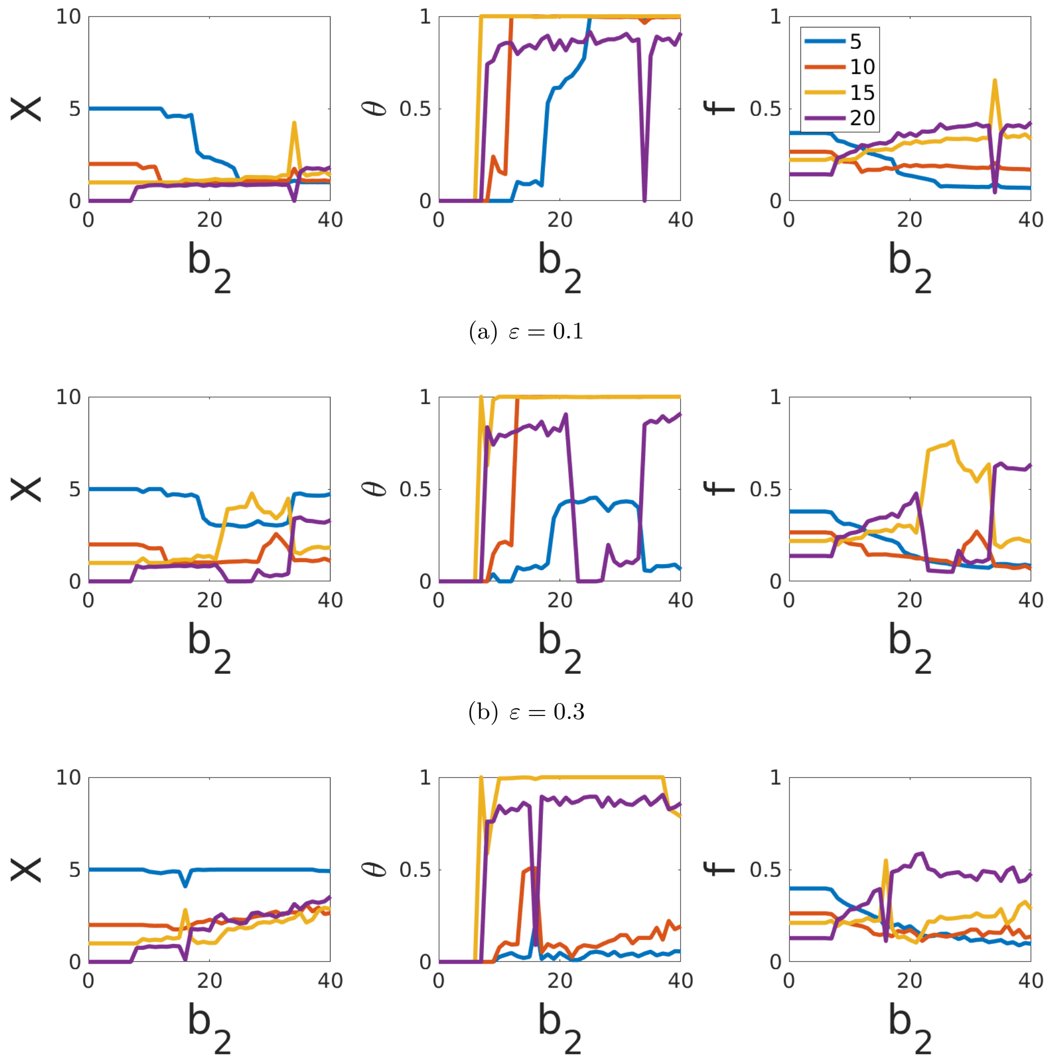

(a) $\varepsilon = 0.1$

(b) $\varepsilon = 0.3$

(c) $\varepsilon = 0.5$

Figure S27: $B_1 = 100$, $Z_0 = 300$

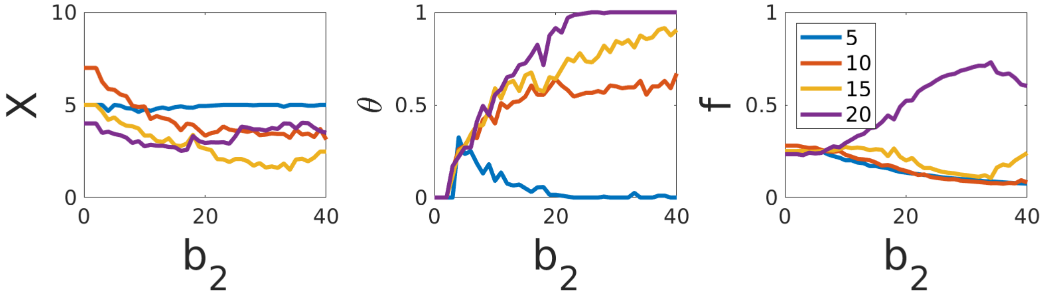

(a) $\varepsilon = 0.1$

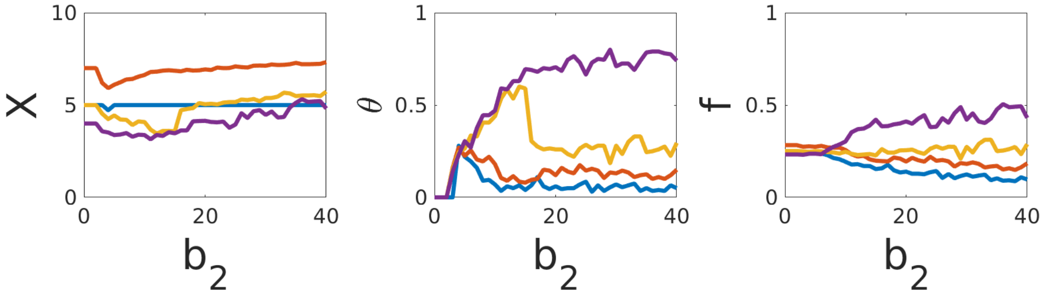

(b) $\varepsilon = 0.3$

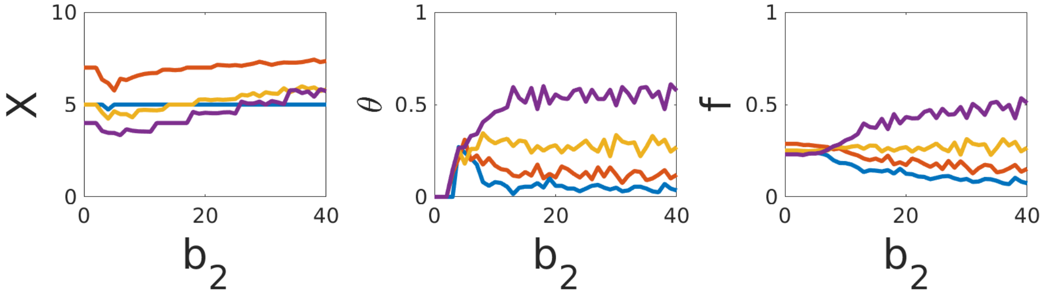

(c) $\varepsilon = 0.5$

Figure S28: $B_1 = 300$, $Z_0 = 50$

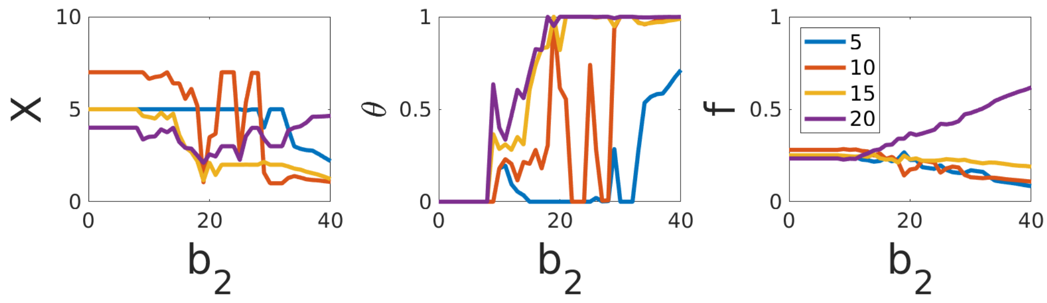

(a) $\varepsilon = 0.1$

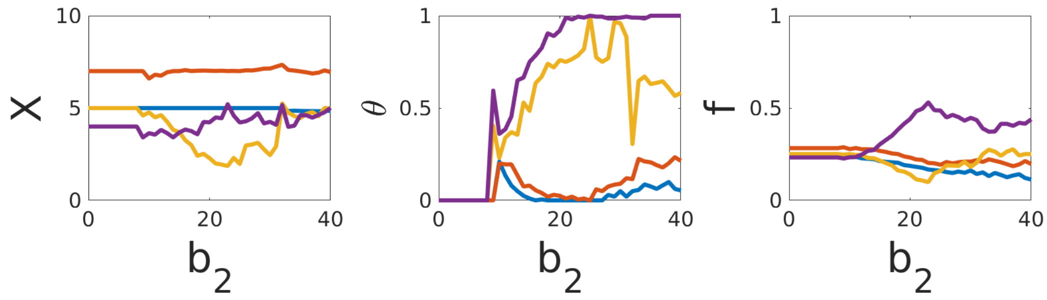

(b) $\varepsilon = 0.3$

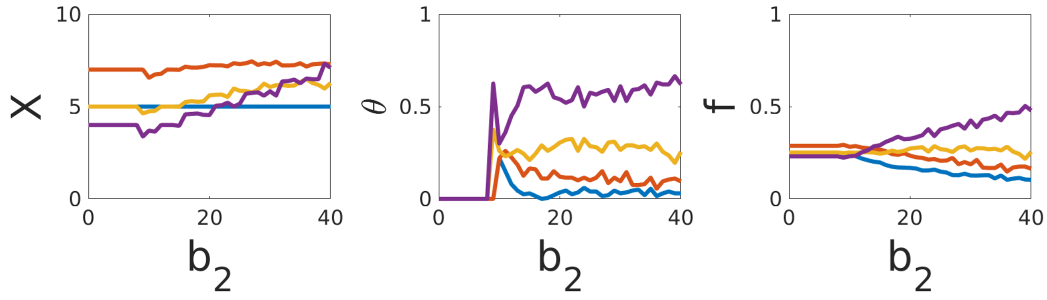

(c) $\varepsilon = 0.5$

Figure S29: $B_1 = 300$, $Z_0 = 300$

2.2.2 Effects of parameters, non-rivalrous goods

Figures S30-S31 show effects of the benefit parameter b_2 on the average number of contributing individuals X, the average cooperation status θ and average power f of each group for different values of the incumbency parameter ε and the benefit parameter B_1. A society of 4 groups containing 5, 10, 15 and 20 individuals respectively is considered. Baseline parameters: $\alpha = 0$, $c = 1$, $\pi^0 = 1$, $X_0 = 5$, $Z_0 = 20$, $\mu_1 = \mu_2 = 0.25$. Initially, each individual and group cooperate randomly with probability 0.5, and equal power of groups. The figures show the averages based on 100 runs each of 4000 time steps for each parameter combination. Results in each run are averages based on the last 1000 time steps.

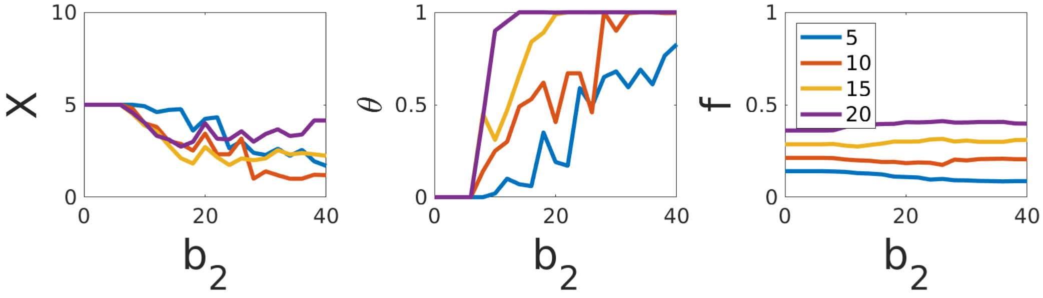

(a) $\varepsilon = 0.1$

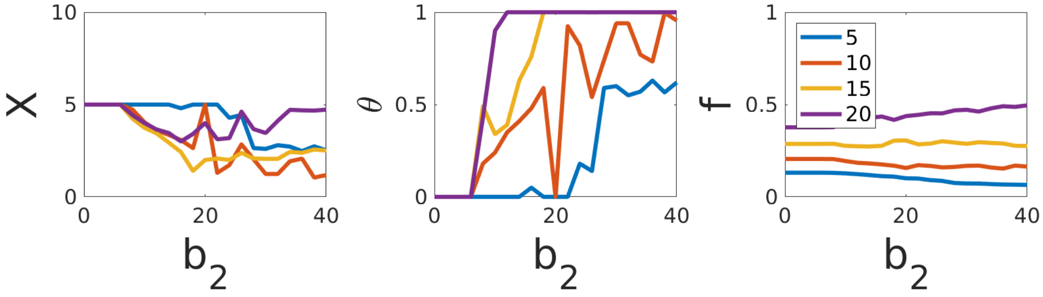

(b) $\varepsilon = 0.4$

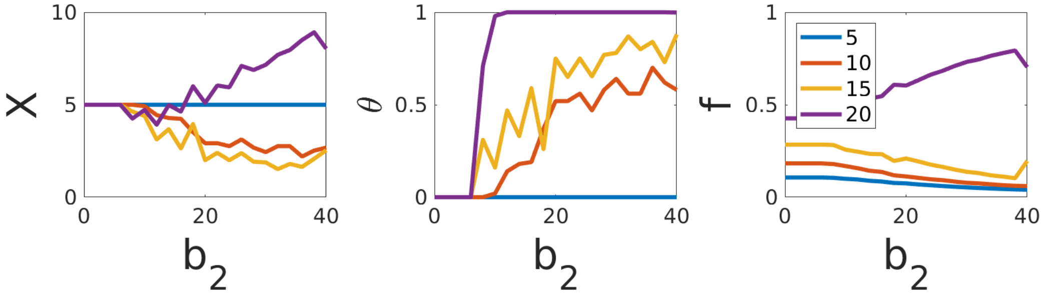

(c) $\varepsilon = 0.7$

Figure S30: $B_1 = 20$

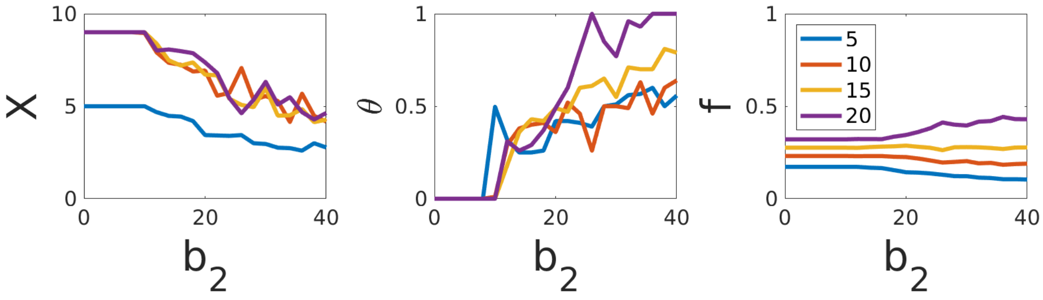

(a) $\varepsilon = 0.1$

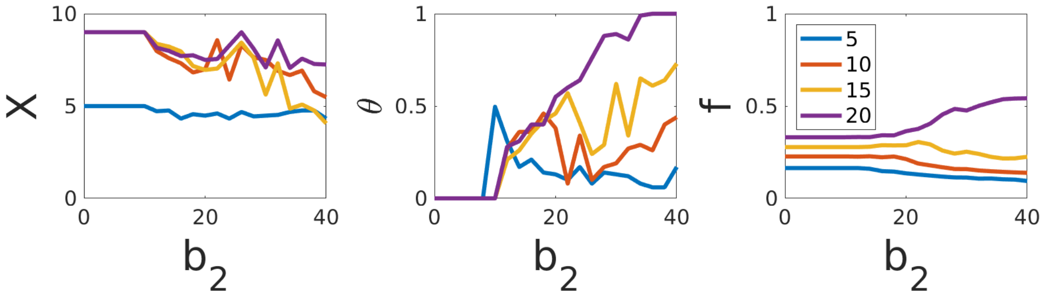

(b) $\varepsilon = 0.4$

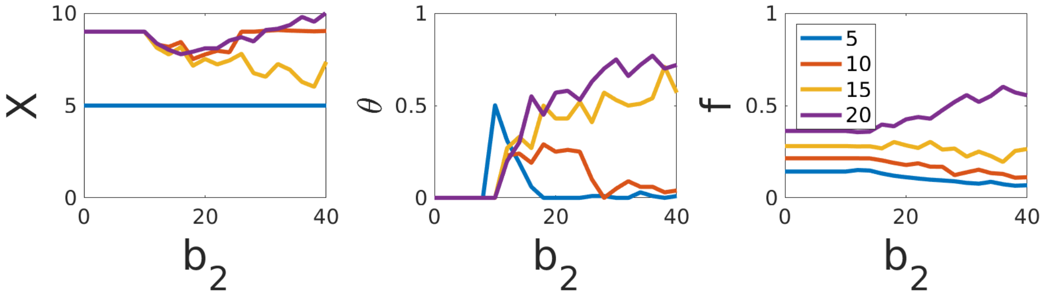

(c) $\varepsilon = 0.7$

Figure S31: $B_1 = 40$

2.2.3 Effects of group size, rivalrous goods

Figures S32-S37 show effects of the group size n on the number of contributing individuals in a group X, the average cooperation status θ of a group and group power f for different values of the incumbency parameter ε, the benefit parameter B_2 and the costs parameter Z_0. Characteristics of groups with 5, 10, 15 and n individuals are marked by blue, red, yellow and violet colors respectively. Baseline parameters: $B_1 = 100$, $\alpha = 1$, $c = 1$, $\pi^0 = 1$, $X_0 = 5$, $\mu_1 = \mu_2 = 0.25$. Initially, each individual and group cooperate randomly with probability 0.5, and equal power of groups. The figures show the averages based on 100 runs each of 4000 time steps for each parameter combination. Results in each run are averages based on the last 1000 time steps.

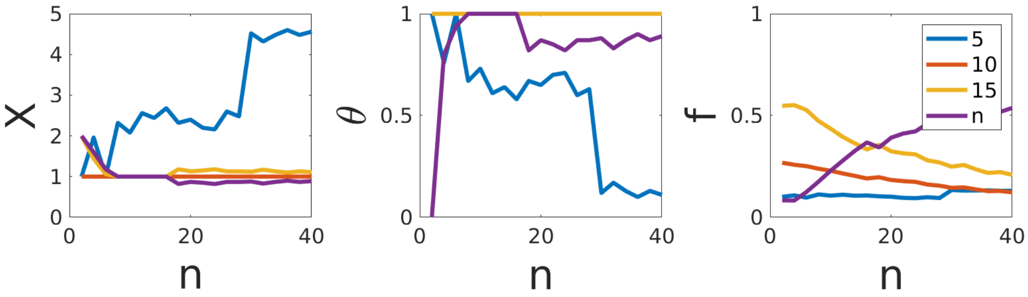

(a) $\varepsilon = 0.1$

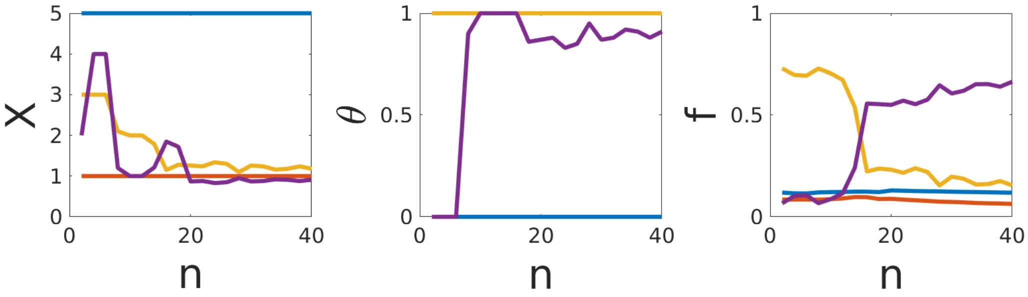

(b) $\varepsilon = 0.3$

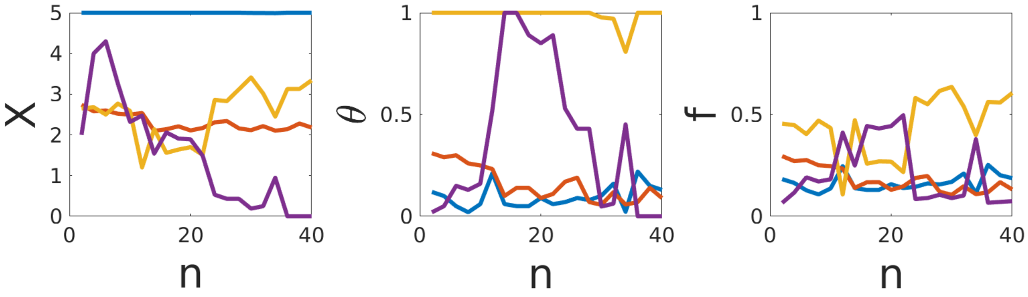

(c) $\varepsilon = 0.5$

Figure S32: $B_2 = 500$, $Z_0 = 50$

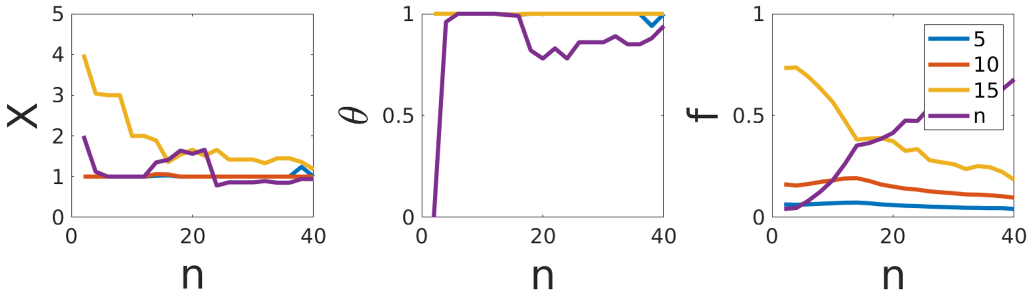

(a) $\varepsilon = 0.1$

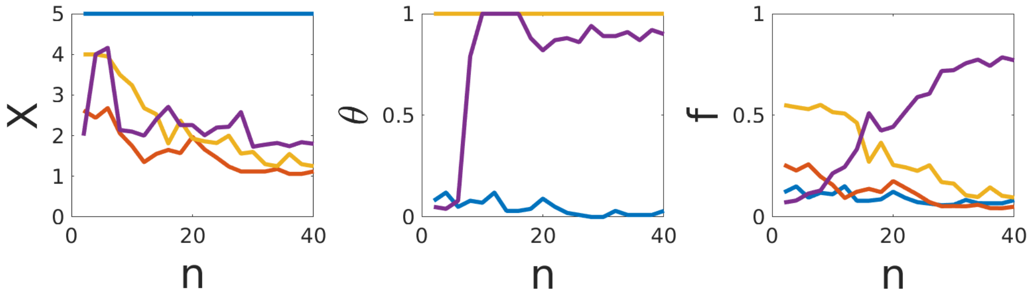

(b) $\varepsilon = 0.3$

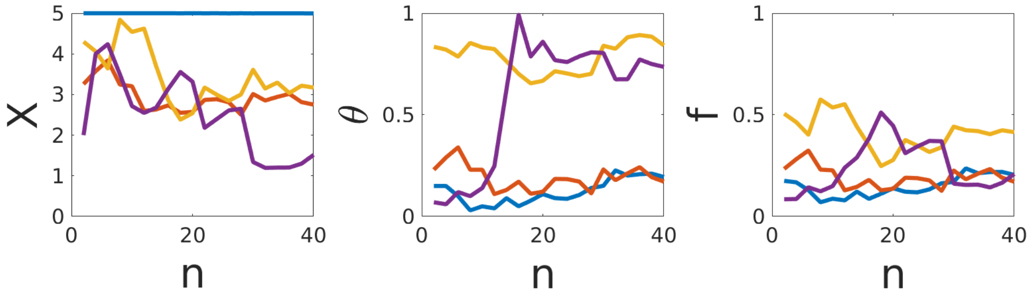

(c) $\varepsilon = 0.5$

Figure S33: $B_2 = 1000$, $Z_0 = 50$

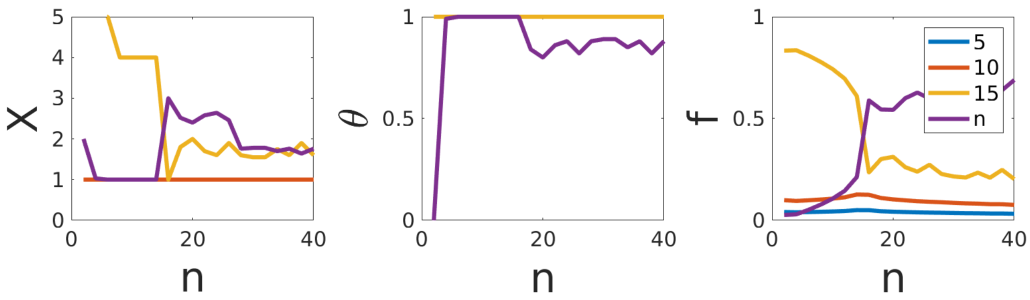

(a) $\varepsilon = 0.1$

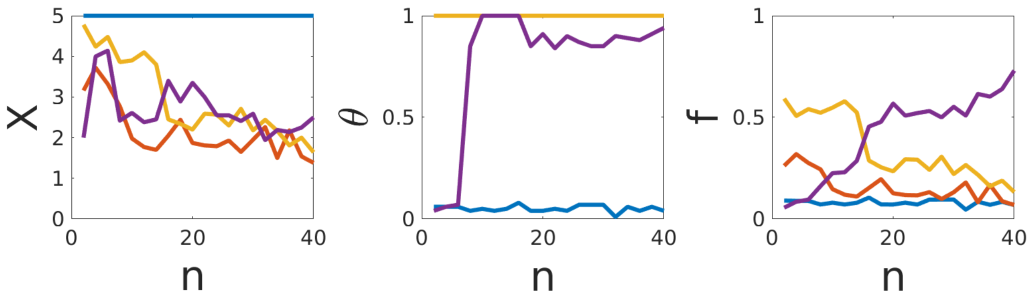

(b) $\varepsilon = 0.3$

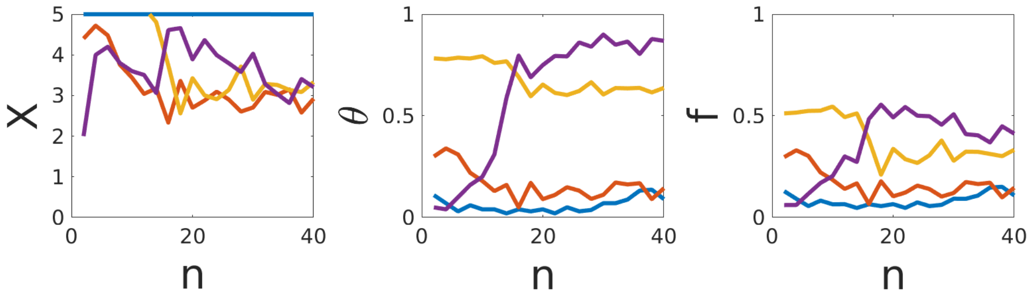

(c) $\varepsilon = 0.5$

Figure S34: $B_2 = 1500,\ Z_0 = 50$

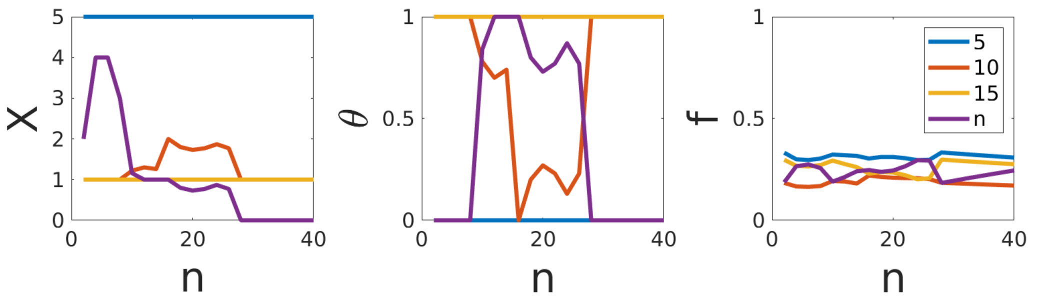

(a) $\varepsilon = 0.1$

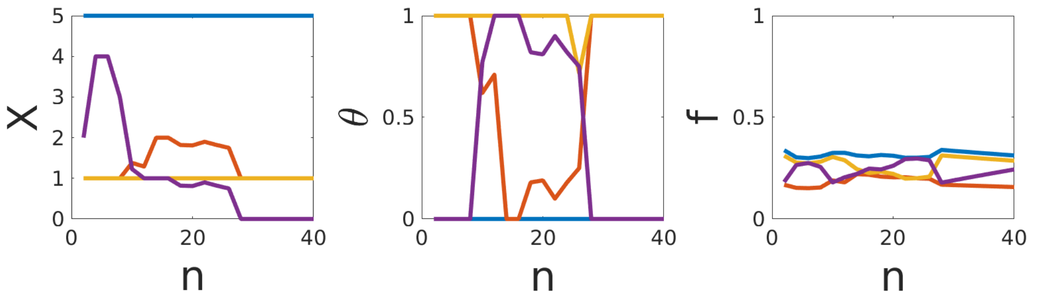

(b) $\varepsilon = 0.3$

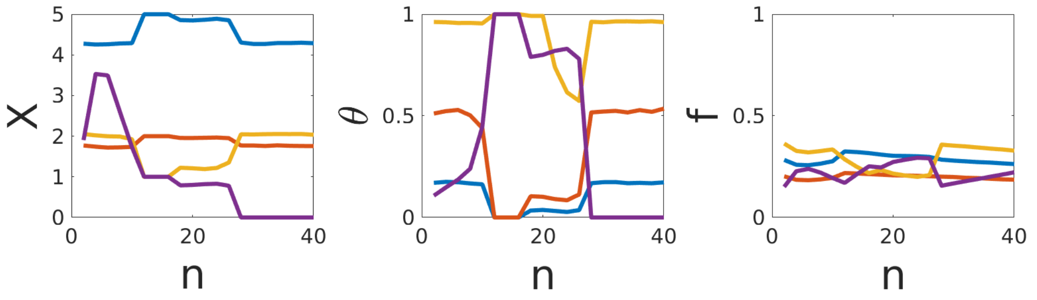

(c) $\varepsilon = 0.5$

Figure S35: $B_2 = 500$, $Z_0 = 300$

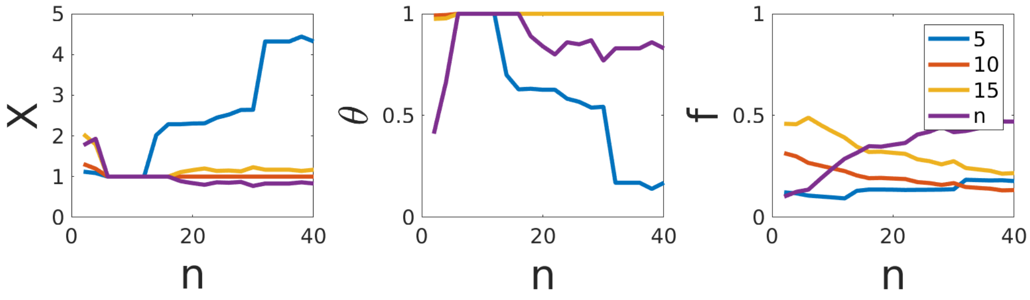

(a) $\varepsilon = 0.1$

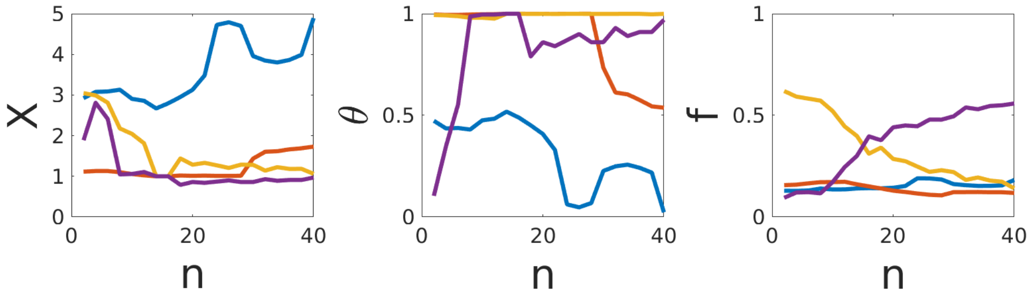

(b) $\varepsilon = 0.3$

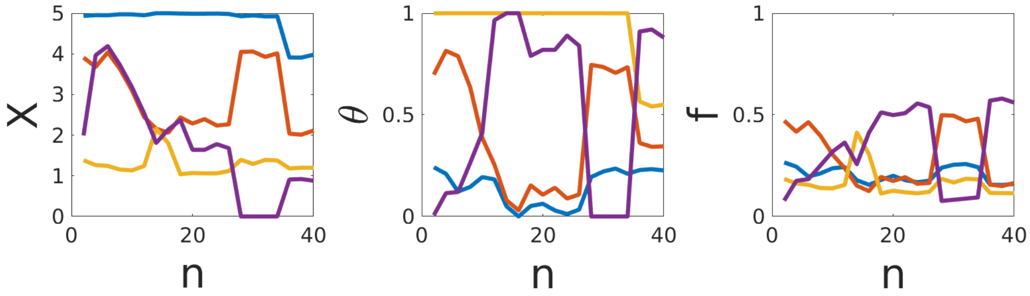

(c) $\varepsilon = 0.5$

Figure S36: $B_2 = 1000,\ Z_0 = 300$

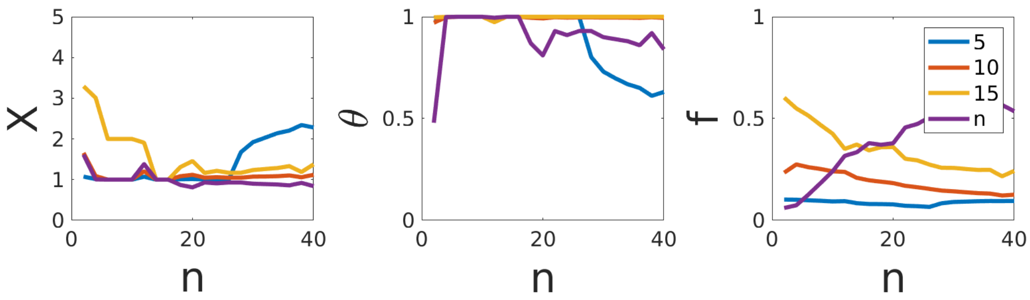

(a) $\varepsilon = 0.1$

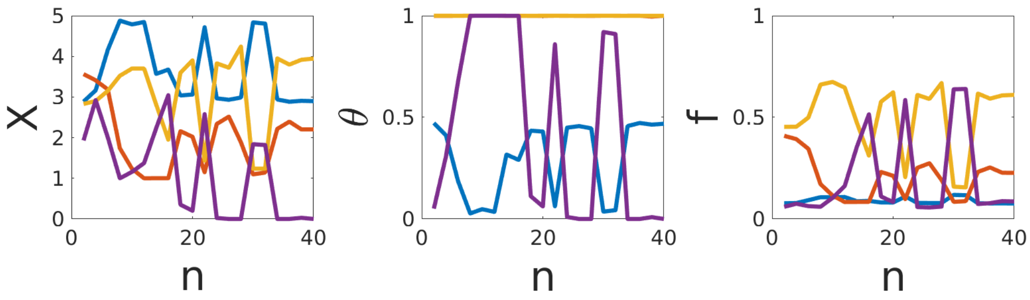

(b) $\varepsilon = 0.3$

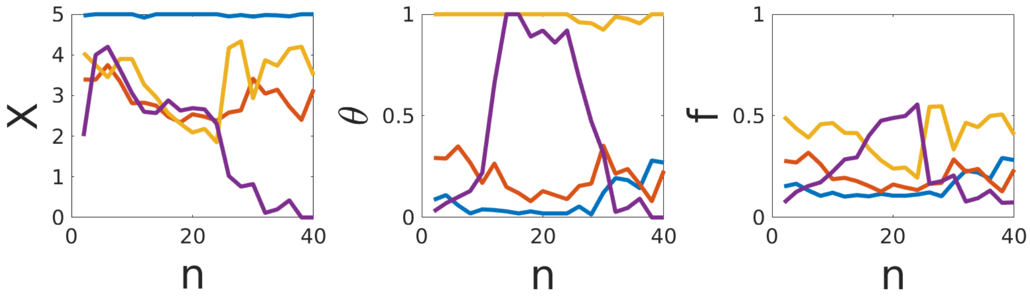

(c) $\varepsilon = 0.5$

Figure S37: $B_2 = 1500$, $Z_0 = 300$

Figures S38-S39 show effects of the group size n on the number of contributing individuals in a group X (a), the cooperation status of a group θ (b) and group power f (c) for different values of the incumbency parameter. Each point corresponds to an outcome of a particular run. Characteristics were calculated as average values among last 1000 of time steps. Characteristics of groups with 5, 10, 15 and n individuals are marked by blue, red, yellow and violet colors respectively. Curves show the average values of corresponding characteristics among all runs. Baseline parameters: $B_1 = 100$, $B_2 = 1500$, $\alpha = 1$, $c = 1$, $\pi^0 = 1$, $X_0 = 5$, $Z_0 = 300$, $\mu_1 = \mu_2 = 0.25$. Initially, each individual and group cooperate randomly with probability 0.5, and equal power of groups. These results are based on 100 runs with 4000 time steps for each parameter combination. The outcomes of each run are averages of the last 1000 time steps. Values of $\theta \notin \{0, 1\}$ indicate runs with non-equilibrium dynamics.

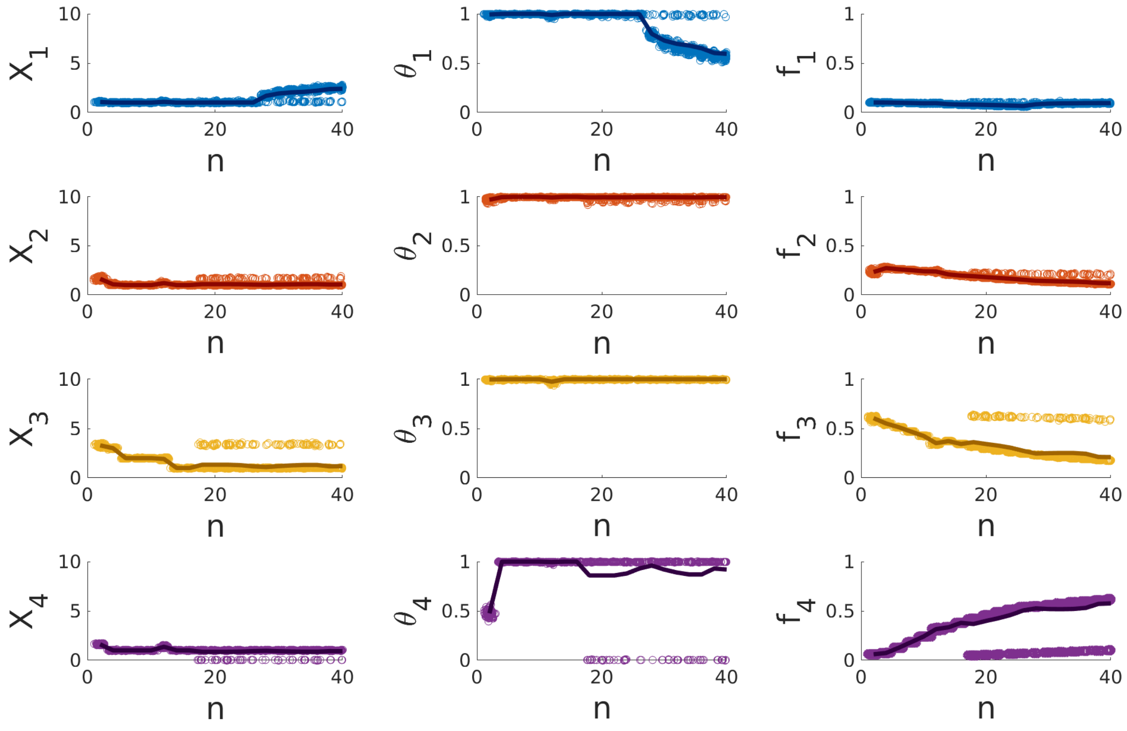

(a) (b) (c)

Figure S38: $\varepsilon = 0.1$

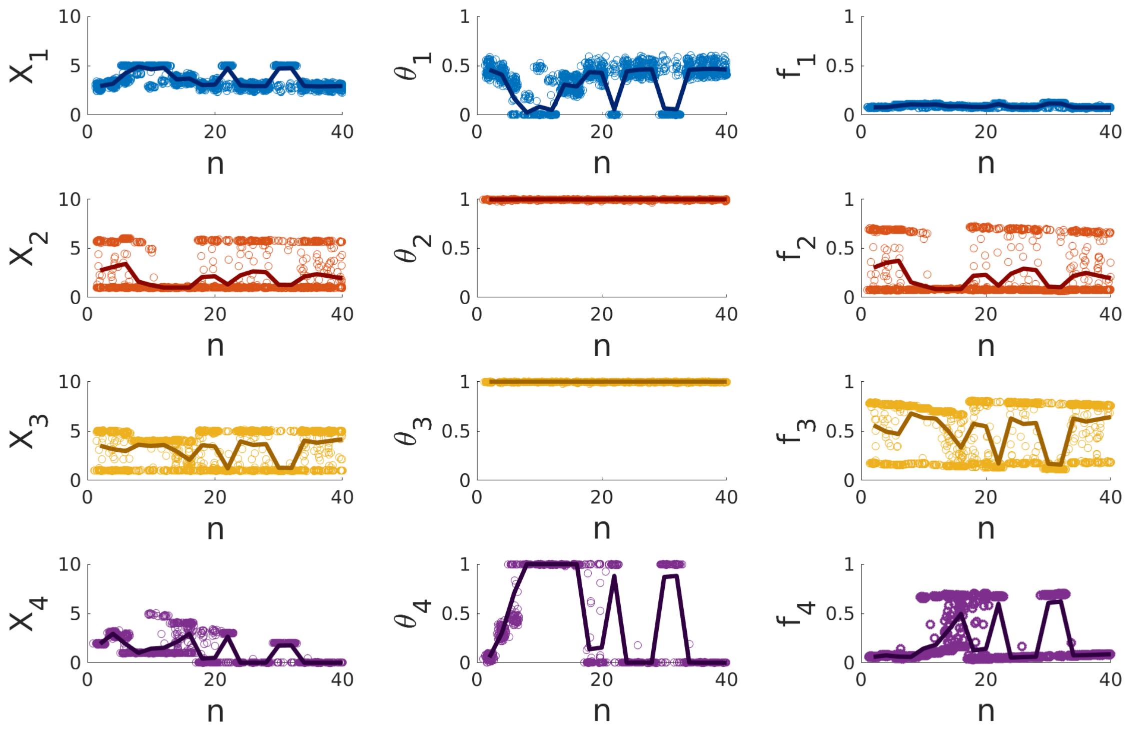

(a) (b) (c)

Figure S39: $\varepsilon = 0.3$

2.2.4 Effects of group size, non-rivalrous goods

Figures S40-S45 show effects of the group size n on the number of contributing individuals in a group X (a), the cooperation status of a group θ (b) and group power f (c) for different values of the incumbency parameter ε and the benefit parameter B_2. Each point corresponds to an outcome of a particular run. Characteristics were calculated as average values among last 1000 of time steps. Characteristics of groups with 5, 10, 15 and n individuals are marked by blue, red, yellow and violet colors respectively. Curves show the average values of corresponding characteristics among all runs. Baseline parameters: $B_1 = 20$, $\alpha = 0$, $c = 1$, $\pi^0 = 1$, $X_0 = 5$, $Z_0 = 20$, $\mu_1 = \mu_2 = 0.25$. Initially, each individual and group cooperate randomly with probability 0.5, and equal power of groups. These results are based on 100 runs with 4000 time steps for each parameter combination. The outcomes of each run are averages of the last 1000 time steps. Values of $\theta \notin \{0, 1\}$ indicate runs with non-equilibrium dynamics.

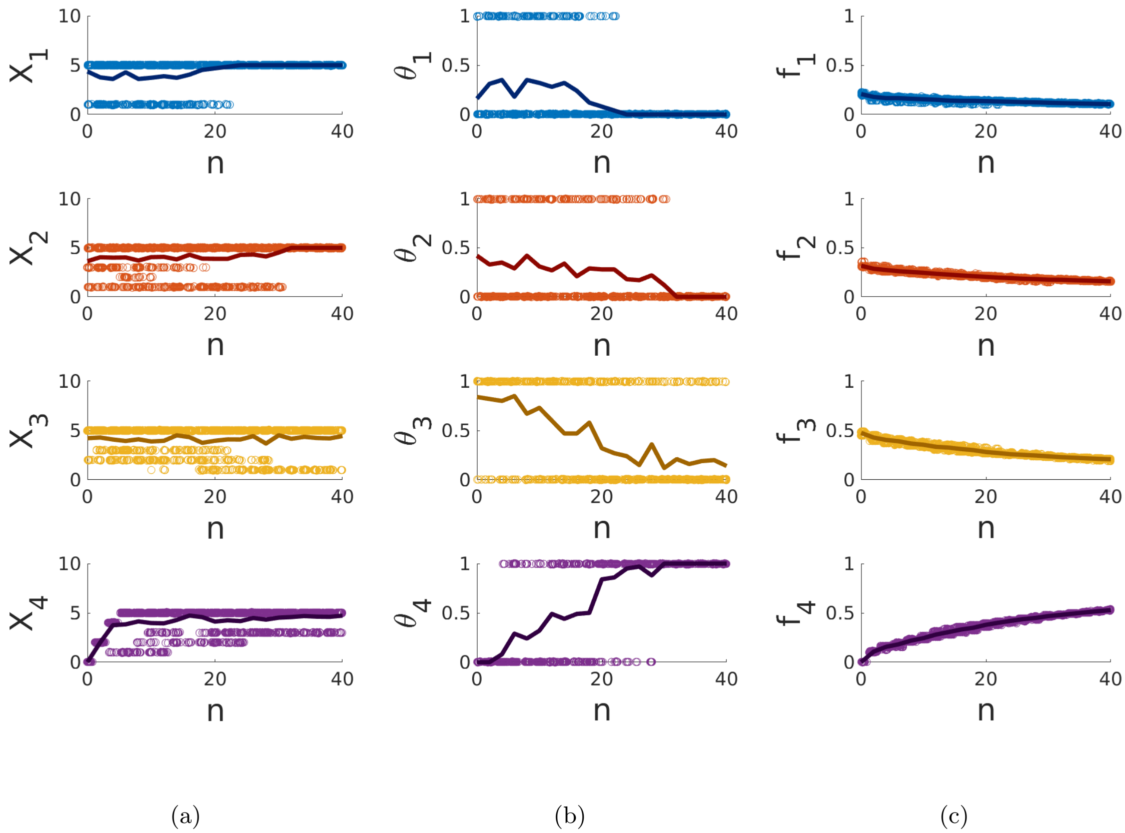

Figure S40: $B_2 = 40, \varepsilon = 0.1$

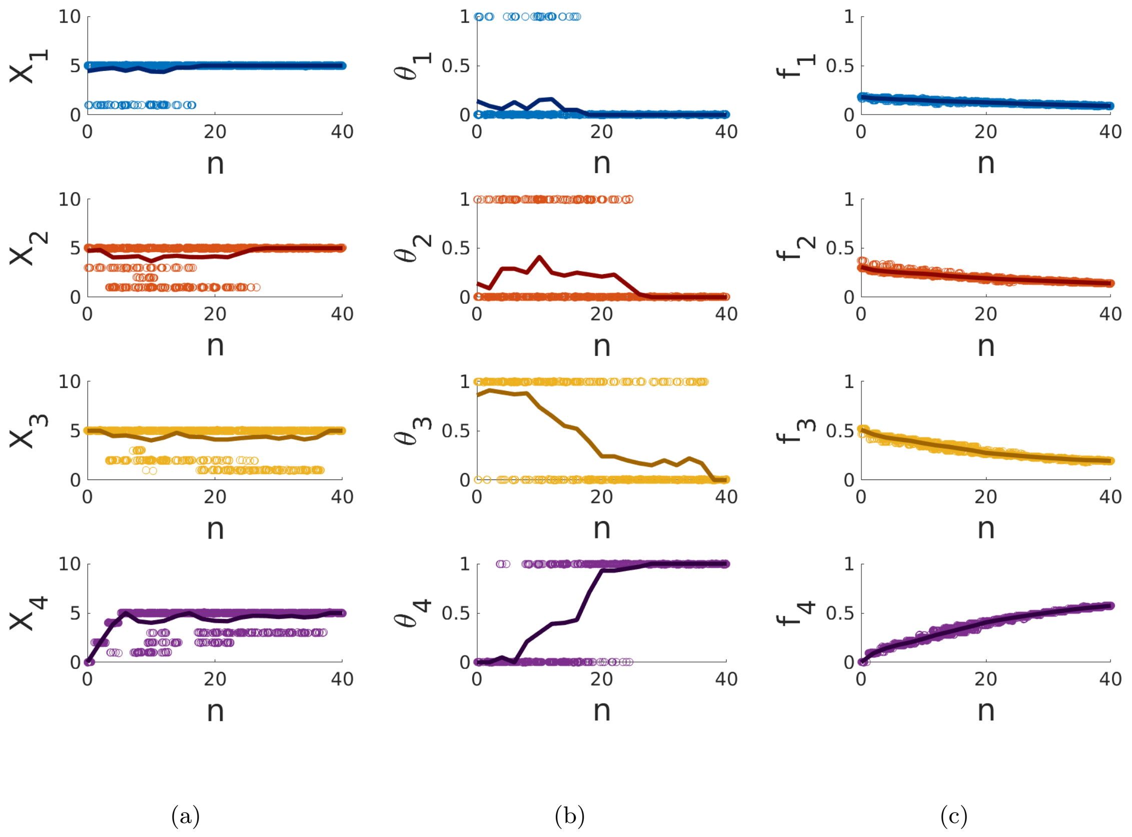

Figure S41: $B_2 = 40, \varepsilon = 0.4$

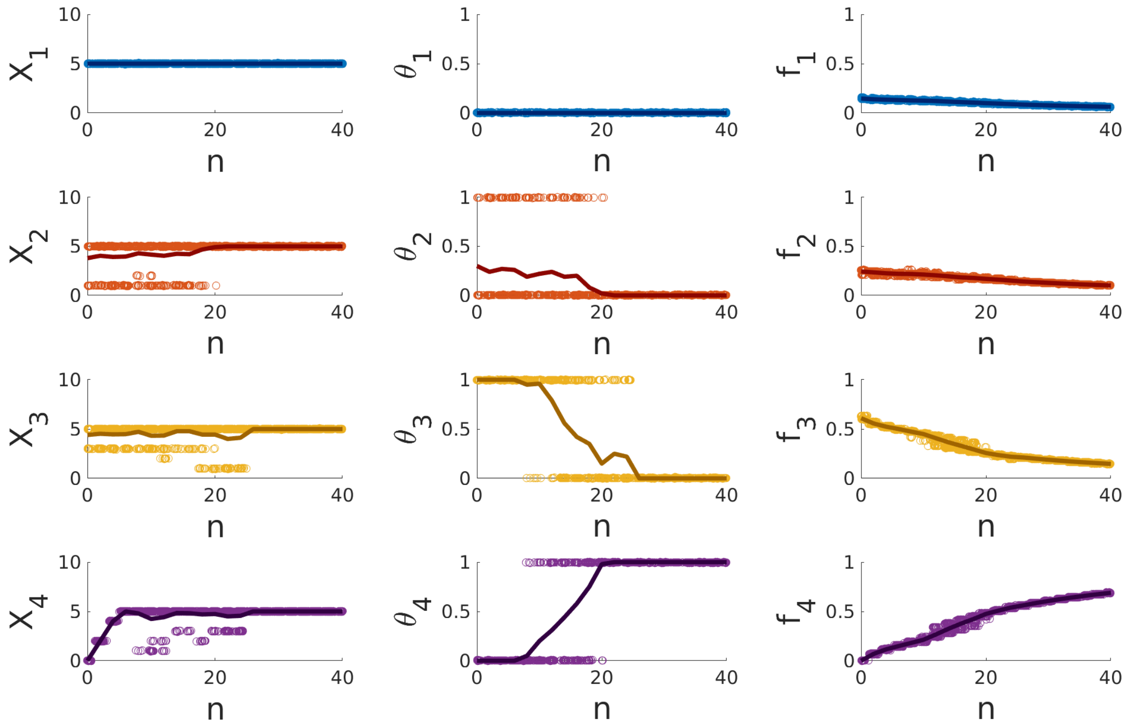

(a) (b) (c)

Figure S42: $B_2 = 40, \varepsilon = 0.7$

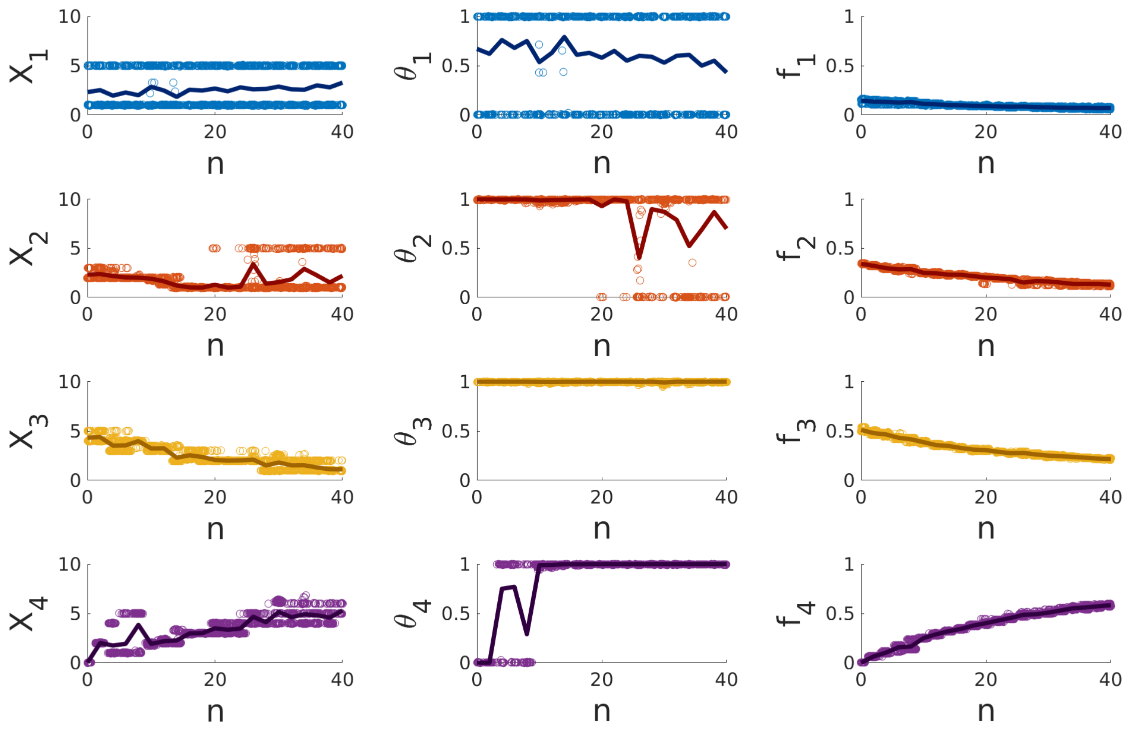

(a) (b) (c)

Figure S43: $B_2 = 120, \varepsilon = 0.1$

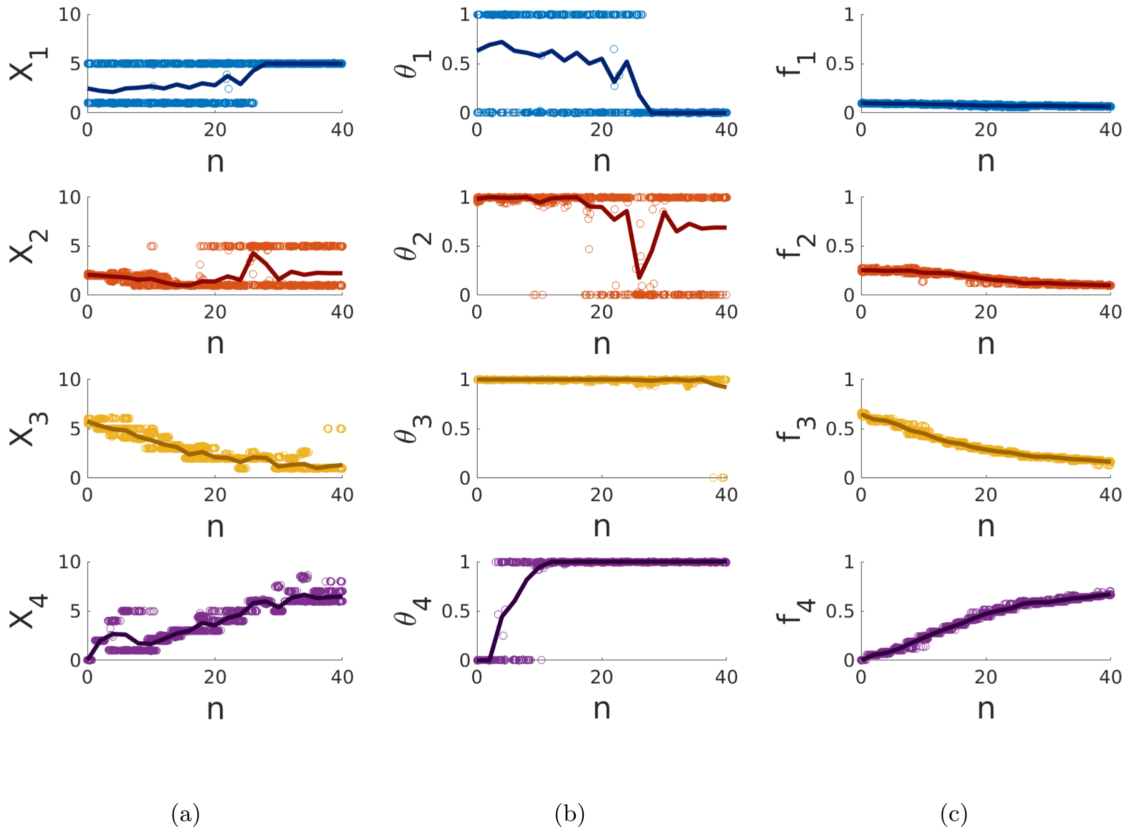

(a) (b) (c)

Figure S44: $B_2 = 120, \varepsilon = 0.4$

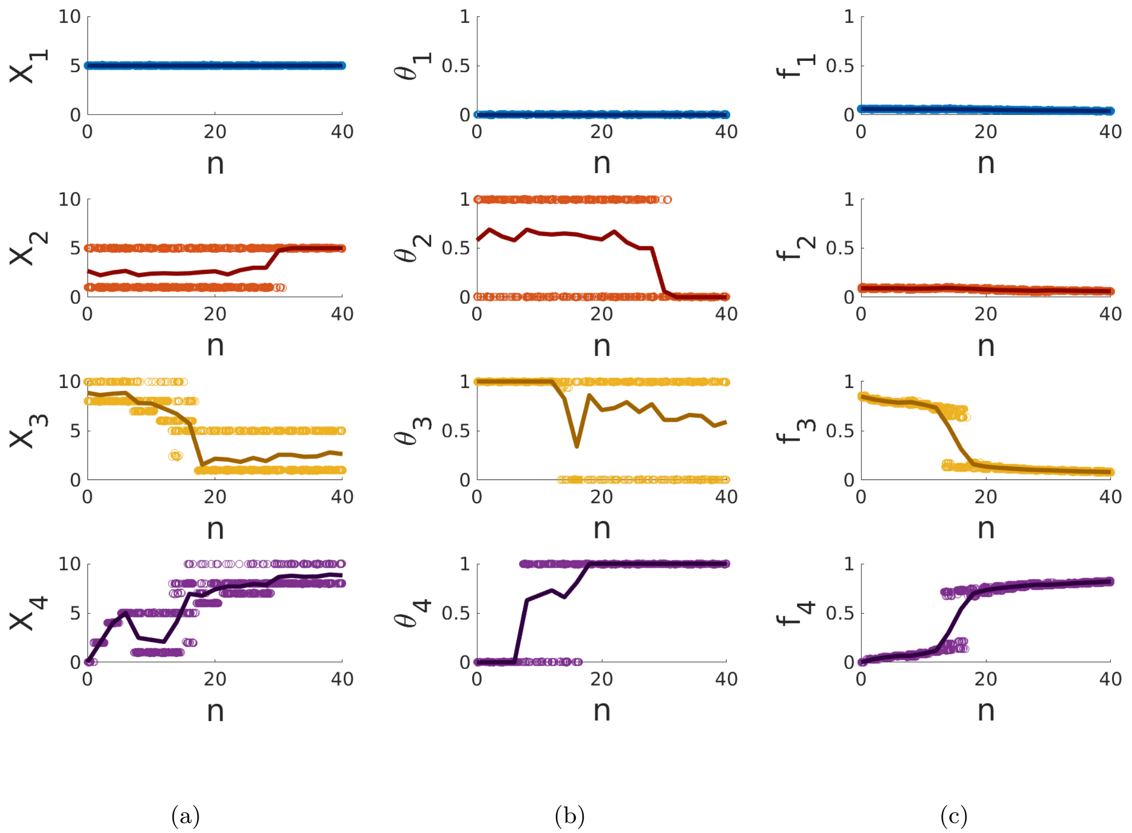

Figure S45: $B_2 = 120, \varepsilon = 0.7$

2.2.5 Non-equilibrium dynamics

Figures S46-S49 show effects of the benefit parameter b_2 on the number of non-equilibrium runs k_{NE}, average group power f and the average cooperation status of a group θ for different values of the benefit parameter B_1 and the costs parameter Z_0. Each point shows the average value of a characteristic among all runs characterized by non-equilibrium dynamics. Characteristics of groups with 5, 10, 15 and 20 individuals are marked by blue, red, yellow and violet colors respectively. Baseline parameters: $\alpha = 1$, $c = 1$, $\pi^0 = 1$, $X_0 = 5$, $\mu_1 = \mu_2 = 0.25$. Initially, each individual and group cooperate randomly with probability 0.5, and equal power of groups. These results are based on 100 runs with 4000 time steps for each parameter combination. The outcomes of each run are averages of the last 1000 time steps.

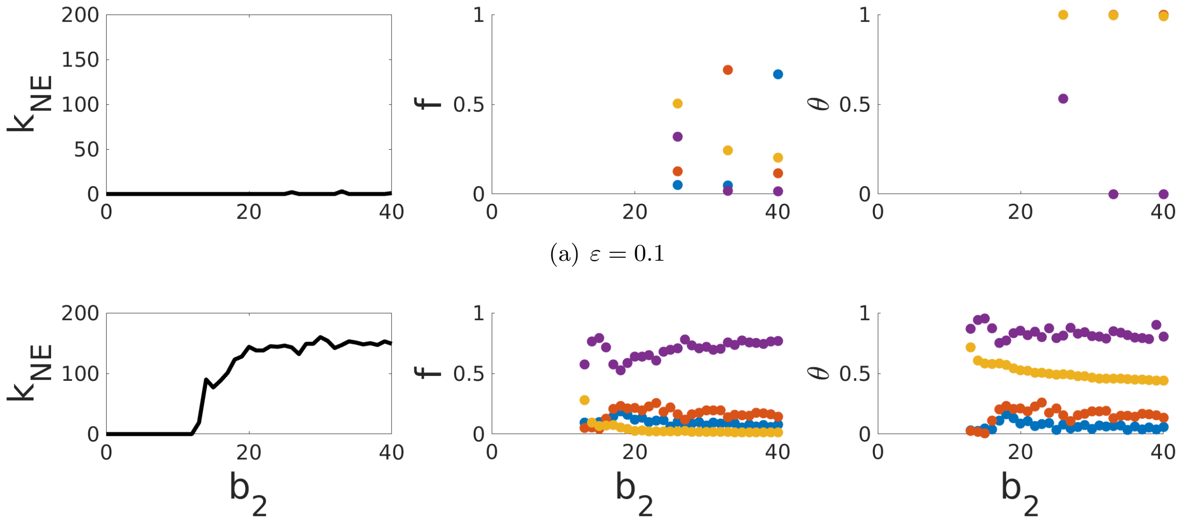

(a) $\varepsilon = 0.1$

(b) $\varepsilon = 0.5$

Figure S46: $B_1 = 100$, $Z_0 = 50$. Since non-equilibrium dynamics does not emerge for $\varepsilon = 0.3$, the case with $\varepsilon = 0.3$ is not shown in the figure.

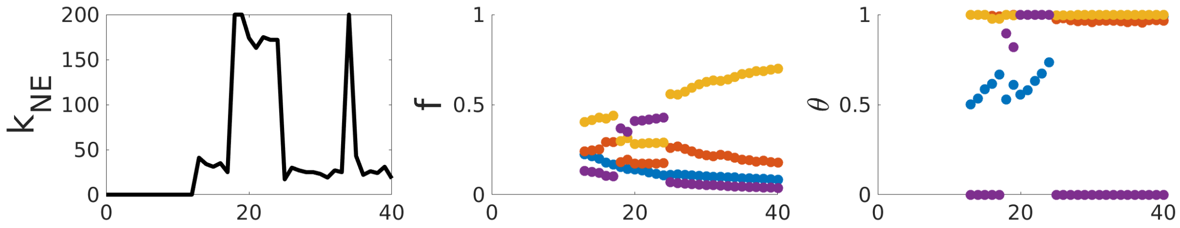

(a) $\varepsilon = 0.1$

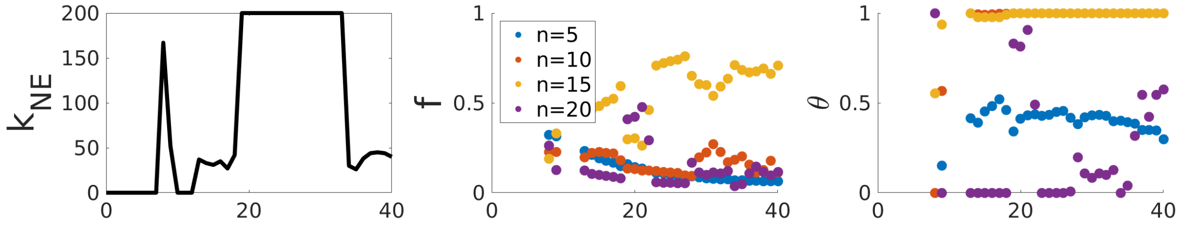

(b) $\varepsilon = 0.3$

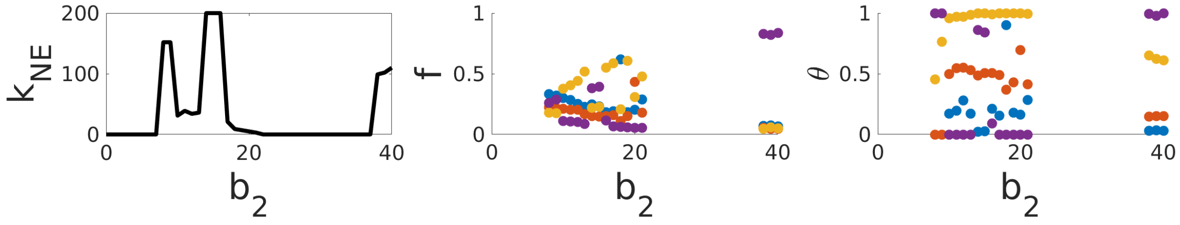

(c) $\varepsilon = 0.5$

Figure S47: $B_1 = 100$, $Z_0 = 300$

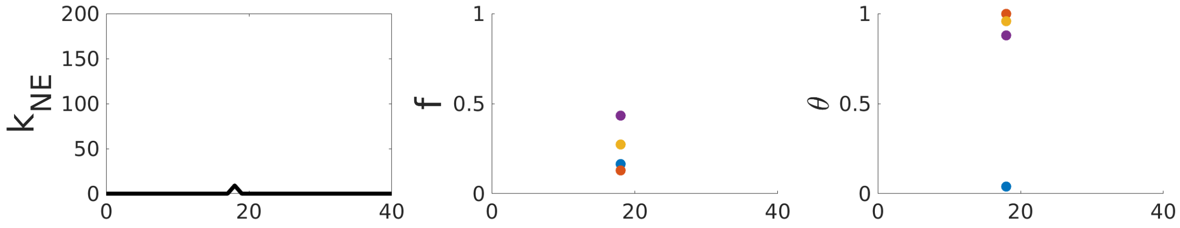

(a) $\varepsilon = 0.1$

Figure S48: $B_1 = 300$, $Z_0 = 50$. Since non-equilibrium dynamics does not emerge for $\varepsilon = 0.3$ and $\varepsilon = 0.5$, cases with $\varepsilon = 0.3$ and $\varepsilon = 0.5$ are not shown in the figure.

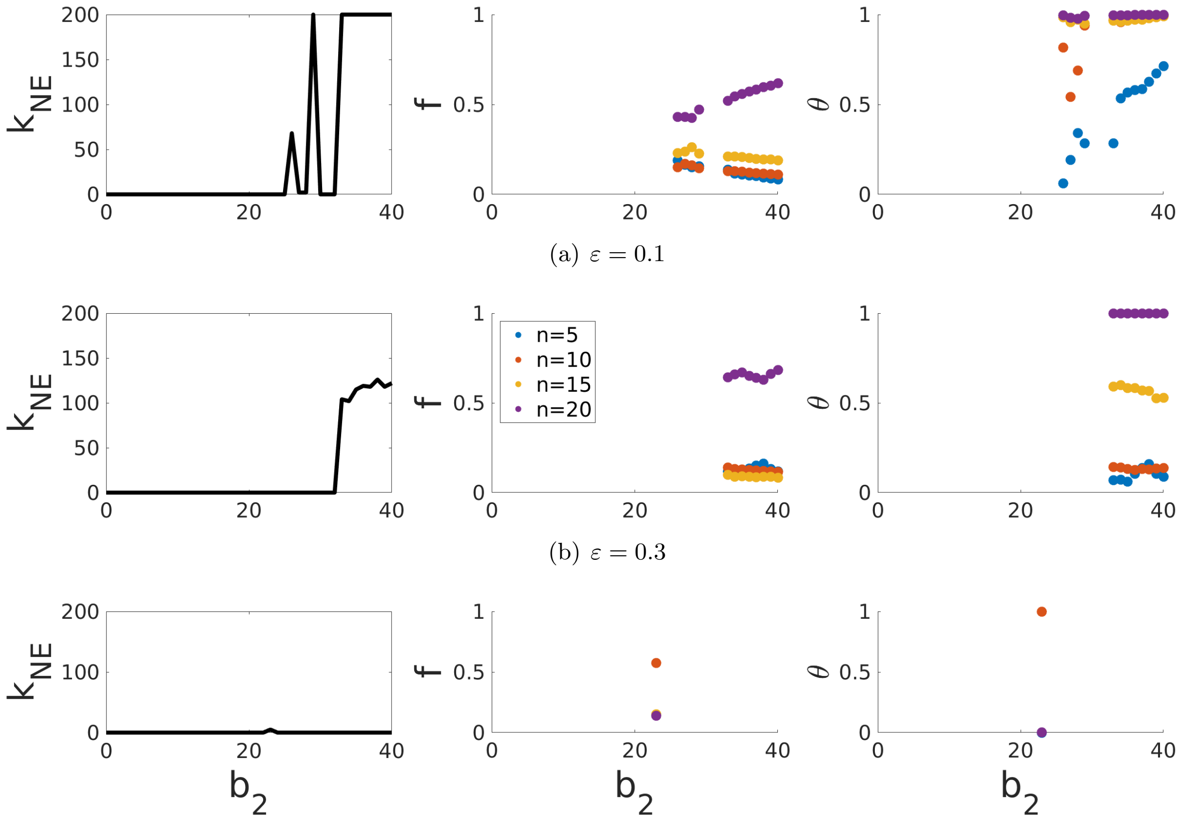

(a) $\varepsilon = 0.1$

(b) $\varepsilon = 0.3$

(c) $\varepsilon = 0.5$

Figure S49: $B_1 = 300$, $Z_0 = 300$
